# Chemical control over the energy-level alignment in a two-terminal junction

**DOI:** 10.1038/ncomms12066

**Published:** 2016-07-26

**Authors:** Li Yuan, Carlos Franco, Núria Crivillers, Marta Mas-Torrent, Liang Cao, C. S. Suchand Sangeeth, Concepció Rovira, Jaume Veciana, Christian A. Nijhuis

**Affiliations:** 1 Department of Chemistry, National University of Singapore, 3 Science Drive 3, Singapore 117543, Singapore; 2 Department of Molecular Nanoscience and Organic Materials, Institut de Ciència de Materials de Barcelona (ICMAB-CSIC) and Networking Research Center on Bioengineering, Biomaterials and Nanomedicine (CIBER-BBN), Campus de la UAB, Bellaterra 08193, Spain; 3 Centre for Advanced 2D Materials and Graphene Research Centre, National University of Singapore, 6 Science Drive 2, Singapore 117546, Singapore; 4 Solar Energy Research Institute of Singapore (SERIS), National University of Singapore, Singapore 117574, Singapore

## Abstract

The energy-level alignment of molecular transistors can be controlled by external gating to move molecular orbitals with respect to the Fermi levels of the source and drain electrodes. Two-terminal molecular tunnelling junctions, however, lack a gate electrode and suffer from Fermi-level pinning, making it difficult to control the energy-level alignment of the system. Here we report an enhancement of 2 orders of magnitude of the tunnelling current in a two-terminal junction via chemical molecular orbital control, changing chemically the molecular component between a stable radical and its non-radical form without altering the supramolecular structure of the junction. Our findings demonstrate that the energy-level alignment in self-assembled monolayer-based junctions can be regulated by purely chemical modifications, which seems an attractive alternative to control the electrical properties of two-terminal junctions.

## Abstract (web summary)

Energy alignment in molecular tunnelling junctions is desirable for altering their electrical properties, however controllability is still an issue. Here the authors report a 2 orders-of-magnitude increase in the tunnelling current via chemical control of the energy-level alignment at a two-terminal junction.

## Graphical abstract

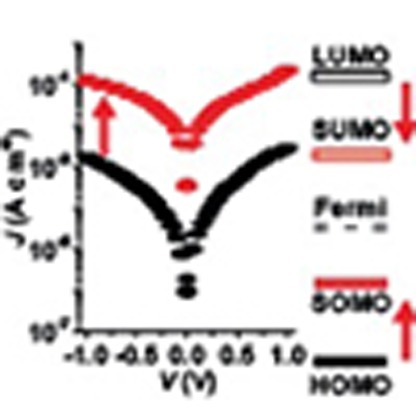

The possibility to integrate functional molecules into electronic devices is one of the promising approaches to miniaturize electronic circuits or to generate electronic function that is difficult to obtain using conventional semiconductors^1^,^2^,^3^. The advantage of molecular-based devices is, in principle, that the conductance can be tuned by designing molecules with the electronic and chemical structure tailored for the desired application. To achieve this ‘chemical control' over transport characteristics, a good control over the energy-level alignment of the molecular frontier orbitals with respect to the Fermi level (*E*_F_) of the electrodes is needed. For instance, control over the energy-level alignment has been used in thin film devices to lower charge injection barriers by either altering the work function of the metal-electrodes^4^, or controlling the HOMO (highest-occupied molecular orbital) and/or LUMO (lowest unoccupied molecular orbital) levels with respect to the Fermi levels by chemical modification^5^,^6^, or introducing charge injection layers^7^,^8^. In practical systems, however, chemical control over the energy-level alignment proves to be challenging because of the so-called ‘pillow effect' or Fermi-level pinning^7^. In molecular electronics, especially in the case of two-terminal devices, it remains difficult to predict how, if at all, certain chemical functionalities alter the junction characteristics^9^,^10^,^11^,^12^,^13^,^14^,^15^. Here we show how the tunnelling rates across molecular junctions can be increased by 2 orders of magnitude by tuning the energy levels of the system within the conduction window without the need for altering the molecular structure of the molecule–electrode interfaces by modifying the electronic structure of the molecules between the open- and closed-shell forms. Since the nature of the molecule–electrode interface is kept the same for both molecular forms, this approach is not limited by Fermi-level pinning.

The molecule bridging the two electrodes provides the molecular energy levels (*E*) in the solid-state device for conduction channels. The sum of the channels (*M*(*E*)) within the electrochemical potential window between *μ*_L_ and *μ*_R_, will result in an effective current, as described by the Landauer formalism^16^,^17^,^18^:


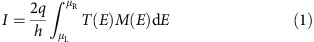


where *h* is Planck's constant, *q* is the electron charge and *T*(*E*) is the transmission probability, which is inversely proportional to the square of zero-bias energy off-set between the molecular orbital and the Fermi level of the electrodes (δ*E*_ME_), equation (2),


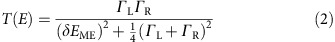


where *Γ*_L_ and *Γ*_R_ are the degree of the coupling strength between the molecular frontier orbital and the Fermi level of the left (L) and the right (R) electrodes, respectively. Thus, the current (*I*) is proportional to the *M*(*E*) and inversely proportional to (δ*E*_ME_) (ref. 2). Although in three-terminal devices the energy-level alignment of the systems, that is, the value of δ*E*_ME_, can be controlled via a gate electrode (or in a wet electrochemical environment gating via the electrolyte is possible)^19^,^20^,^21^,^22^, in two-terminal devices, however, the molecular orbitals cannot be gated and therefore alternative approaches are needed.

One of these approaches is to control the energy-level alignment of the system by modifying the chemical structure of the molecules inside the junctions. In reality, it is difficult to predict how changes to the chemical structure of the junctions affect the electrical properties of the junctions and seemingly contradicting results have been reported. In SAM-based junctions, the difficulty of controlling the energy-level alignment due to Fermi-level pinning has been well-recognized.^7^,^23^ For example, Frisbie and co-workers^24^ showed that changing the work function of the metal had a noticeable effect on the contact resistance (due to a large surface dipole at the metal–thiolate interface) but not on the energy-level alignment. Similarly, Blom and co-workers^25^ found that introducing additional dipoles in the SAM structure results in large work function shifts. In contrast, Whitesides and co-workers^13^,^14^,^15^ observed that the tunnelling rates across aliphatic SAMs in EGaIn junctions were independent of dipoles or acidity of small end groups of alkanethiolate SAMs. We confirmed these findings by Whitesides and co-workers^15^ for a small subset of SAM structures, but Whitesides and co-workers and we also showed that other functionalities, such ferrocene (Fc) and redox-active aromatic groups^26^,^27^,^28^, or polarizable groups (halogens)^29^, did change the junction characteristics and induced rectification or changed the tunnelling rates, respectively. These studies show, as a group, that it is indeed difficult to predict which kind of chemical functionalities result in a noticeable change in the electrical characteristics of the junctions. One of the reasons is that changes in the molecular structure also result in changes of the molecule–electrode interactions and the supramolecular structure of the SAM making it difficult to isolate the factors that dominate the charge transport rates.

In this study, the charge transport rate across SAM-based tunnelling junctions with the same electrode–molecule interfaces and supramolecular structure but with different electronic structure modified by chemically switching the molecule between the open- and closed-shell forms is investigated. In particular, we incorporate SAMs of polychlorotriphenylmethyl radical (PTM^R^) and of non-radical polychlorotriphenylmethane (PTM^NR^) molecules tethered to the Au bottom electrode via an alkanethiolate linker into EGaIn junctions. The open-shell form contains a SOMO (single occupied molecular orbital), with one electron with an *α* spin configuration, and a SUMO (single unoccupied molecular orbital), and this results in a smaller molecular energy SOMO–SUMO gap than the HOMO–LUMO gap of the closed-shell form (Δ*E* values in Fig. 1). Similar observations have been made in an organic thin film in a wet electrochemical environment^25^. Thus, we change the electronic structure of the PTM while keeping the PTM—electrode interactions the same as the PTM is separated from the top-electrode via a van der Waals interface and the bottom-electrode via the alkyl group. Moreover, we show that not only the energy gap is lowered but also the value of δ*E*_ME_ (or in other words, the energy-level alignment changed) which results in a low tunnelling barrier height and thereby a high-tunnelling current.

We investigated the mechanism of charge transport through a SAM of a free organic radical based on PTM radical because it is stable and can be readily grafted on gold^30^,^31^, glass^32^ and ITO^33^, or as a single molecule linked between gold electrodes showing a Kondo effect at low temperatures^34^. The PTM radical has one unpaired electron located on the central carbon atom with a *sp*^2^ hybridization, which is structurally shielded by the bulky *o*-chlorine atoms, leading to a high chemical and thermal stability^35^. This radical can be readily converted to the alpha-H non-radical derivative via reduction to the anion and protonation of the central carbon. Previously, the charge transport rates across PTM^R/NR^ SAMs on gold were investigated by conductive probe atomic force microscopy (cpAFM), showing a higher tunnelling rate across junctions with PTM^R^-based SAMs than those junctions with PTM^NR^-based SAMs^36^,^37^. Theoretical calculations of the electronic structure of the gas phase molecules suggested SUMO-assisted transport in the case of the radical-based SAM. Based on these calculations, it was assumed that the mechanism of charge transport was coherent tunnelling without direct experimental evidence regarding the electronic structure of the junction- or temperature-dependent charge transport data. This assumption, however, may have been not correct because in these studies the PTM moiety was grafted to the bottom-electrode via a short-conjugated tether. Likely significant hybridization of the molecular frontier orbital with the gold electrode occurs and resonant transport cannot be excluded.

Here we demonstrate that the mechanism of charge transport is coherent tunnelling in both types of PTM^R/NR^ SAMs bearing long (6 or more CH_2_ units) non-conjugated alkyl tether, to ensure the molecular frontier orbitals remain localized on the PTM and that tunnelling rates across the junction can be increased by 2 orders of magnitude in the open-shell based SAMs with respect to the closed-shell ones without changing the tunnelling distance *d* or the nature of the molecule–electrode contacts. This increase in the tunnelling rates is because in junctions with the PTM^R^ SAMs, the SUMO participates in the transport and effectively lowers the tunnelling barrier height (see Fig. 1). Importantly, contrary to what we hypothesized in previous works^36^,^37^, the findings reported here unambiguously demonstrate that the enhancement of the tunnelling rate transport across the radical-based SAMs is not due to a resonant tunnelling mechanism but instead due to a SUMO-assisted coherent non-resonant tunnelling. We base our conclusions on statistically large numbers of *J*(V) data and *J*(V,T) measurements combined with a detailed physical-organic study of the electronic and supramolecular SAM structures using six newly designed PTM derivatives with different chain lengths. All SAMs are anchored on gold via a thiolate binding group and the PTM^R/NR^ moieties are effectively decoupled from the electrode via the long alkyl chains. Hence, all types of SAMs yield similar work functions of gold. Here we show intramolecular control over the electronic structure of the junction, resulting in a large change in the value of δ*E*_ME_ and, consequently, the observed tunnelling rates. In other words, we show a large modulation of the tunnelling rate of 2 orders of magnitude across junctions with the same supramolecular structure and work function of the electrodes.

## Results

### Molecular synthesis

Three PTM-CH=CH-(CH_2_)_*n*−2_SH radicals and three αH-PTM-CH=CH-(CH_2_)_*n*−2_SH non-radicals, with *n*=8, 10 and 12, have been synthesized. We use the abbreviations HSC_*n*_PTM^R/NR^ for simplicity to follow the discussions. Figure 2 shows the synthetic route to the PTM-thiolated derivatives HSC_*n*_PTM^R/NR^. We aimed to couple the alkyl chain with the thiol-anchoring group to the PTM unit using a C=C bond, because the double bond only causes a small modification to the electronic structure of the PTM (unlike electron donating/withdrawing groups such as amides or carbonyls) and it can be readily formed by a Wittig–Horner reaction between the PTM-phosphonate and an aldehyde bearing the corresponding alkyl chain and the thiol-precursor^38^. To overcome the instability of the thiol groups under the Wittig–Horner conditions, as well as under the oxidative conditions needed to generate the radical from the corresponding carbanion, we used a triphenylmethyl (trityl) as a protecting group. This protecting group was easily deprotected in acidic media in the last step of the synthesis to obtain the final thiolated compounds (Fig. 2). High-pressure liquid chromatography (HPLC) confirmed that, despite the reducing character of the thiol groups and the low-reduction potential of the PTM radicals^21^, PTM-thiolated derivatives are stable for several weeks in ambient conditions. We note that storing for extended periods of time disulfide derivatives formed as a result of the oxidation of the thiol groups. The same was observed for the non-radical counterparts and, for this reason, all SAMs reported here were prepared using freshly deprotected thiolate derivatives.

#### SAM structural characterization

The SAMs were prepared using freshly template-stripped Au surfaces with ultra-flat topography^39^,^40^,^41^, which were immersed in 0.5 mM solutions of the target compound in toluene. Before the fabrication of the top-electrode, the SAMs were characterized by cyclic voltammetry (CV) and angle-resolved X-ray photoelectron spectroscopy (ARXPS) to ensure that we used good-quality SAMs (See Supplementary Fig. 10 and Supplementary Figs 13–16 for the complete data sets). The ARXPS and element ratios (see more details in Method section ‘Photoelectron spectroscopy' and ‘Determination of the thickness of the SAMs from the S 2*p* spectra') analysis from XPS revealed that both radical and non-radical SAMs are in a standing-up phase rather than lying flat on the surface, the calculated layer thickness (*d*) scales with *n* and was similar for PTM^NR^ and PTM^R^ SAMs with the same number of *n*, and that all SAMs had very similar surface coverage (1.4–1.6 × 10^−9^ mol cm^−2^) within experimental error (5%) (see Supplementary Table 1 and Methods). From our results, we conclude that the supramolecular structure of the SAM does not change when the PTM units are in the open- or closed-shell forms.

#### SAM electronic structure characterization

The electronic structure of the SAMs on Au^TS^ was determined by ultraviolet photoelectron spectroscopy (UPS) and near edge X-ray adsorption fine structure spectroscopy (NEXAFS) to estimate the positions of the filled and empty states, respectively. The UPS spectra were recorded to determine their energy levels with respect to the Fermi level of a clean Au substrate for all PTM^R/NR^-Au^TS^ SAMs. Figure 3a shows the UPS spectra of the HSC_*n*_PTM^R^ SAMs. We found that the SOMO peak with a SOMO-onset value of ∼1.45 eV is clearly visible in contrast to the spectra obtained from HSC_*n*_PTM^NR^ SAM that do not show this peak (here only HSC_12_PTM^NR^ SAM is shown as representative of the PTM^NR^ SAMs; see Supplementary Fig. 17 for *n*=8 and 10) and only reveal the HOMO peak with the HOMO-onset value at ∼2.45 eV. This is a clear evidence of the persistence of the unpaired electron once the molecules are covalently grafted on the surface.

The C K-edge NEXAFS spectra (Fig. 3b) show peaks at ∼285.7 and ∼286.5 eV, which are assigned to the C(Ph)→π* transitions in the perchlorinated phenyl rings of the PTM molecules^42^. These peaks are present in the spectra of PTM^R^ and PTM^NR^ SAMs, but in the case of the PTM^R^ SAMs there is an additional peak at ∼282.9 eV. This peak indicates the presence of an empty state with an energy just above the Fermi level and it is attributed to the transition to the SUMO orbital^42^,^43^. We have calculated the SUMO (for the PTM^R^ SAMs) and LUMO (for the PTM^NR^ SAMs) energy position from the NEXAFS spectra (Table 1, see details in Methods). Thus, the molecular energy gap of the PTM^R/NR^ SAMs could be calculated from UPS and NEXAFS spectra (Table 1) and the results are comparable with the optical band gap (∼1.9 eV for R and ∼3.9 eV for NR) determined by the ultraviolet–visible spectra (see Supplementary Figs 6 and 7).

**Figure 1:**
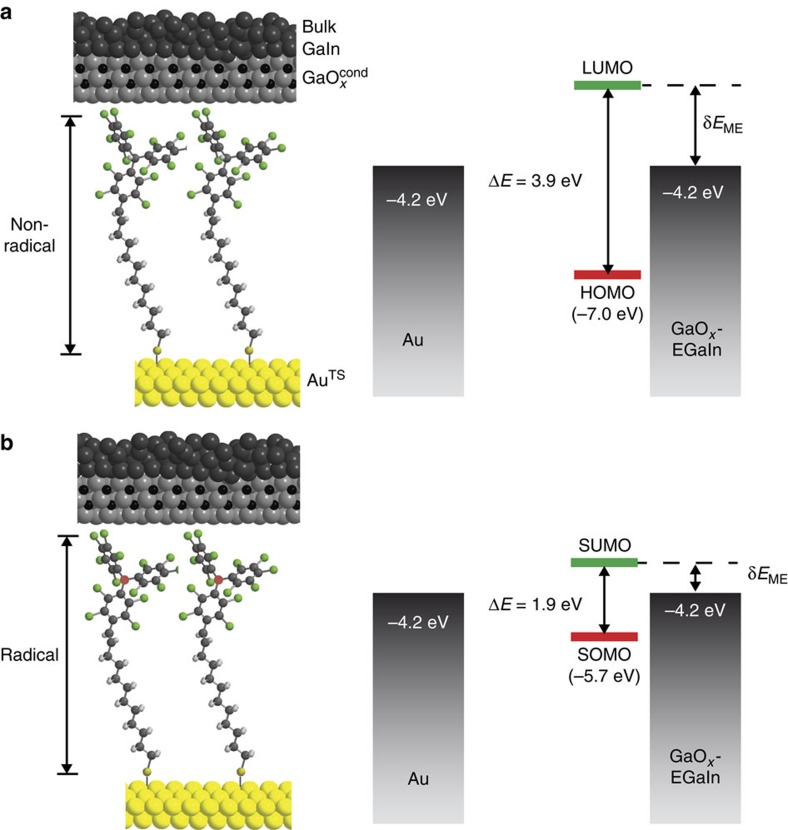
Schematic illustrations of the junctions. Schematic representations of the junctions of Au^TS^-SC_10_PTM^R/NR^//GaO_*x*_^cond^/EGaIn and the corresponding energy level diagrams. The top-electrodes (**a**) are liquid metal GaO_*x*_^cond^/EGaIn (where EGaIn is the eutectic alloy of Ga and In, and GaO_*x*_^cond^ is a 0.7-nm-thick self-limiting highly conductive oxide layer)^15^,^16^,^17^. The bottom electrodes (**b**) are template-stripped Au surfaces (∼300-nm-thick, see fabrication methods in ref. 11 and Methods for details). The Δ*E* and δ*E*_ME_ represent the molecular energy gap and zero-bias energy off-set between the LUMO (or SUMO) and the Fermi level of the electrodes, respectively. Red dots show the central C atoms of radical moieties. The distance (*d*) between two electrodes is about 2.1 nm.

**Figure 2:**
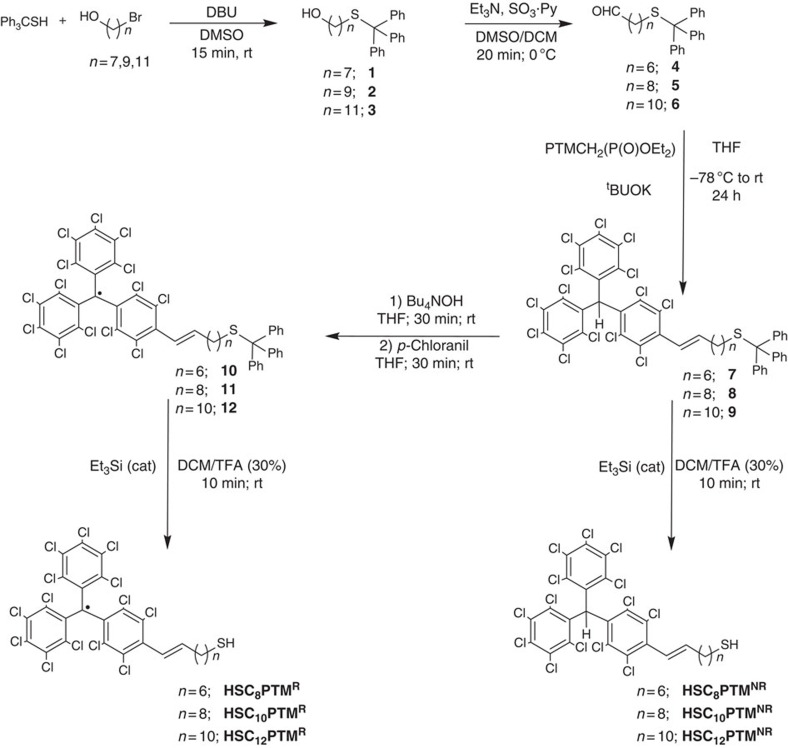
Synthetic route to radicals HSC_*n*_PTM^R^ and non-radicals HSC_*n*_PTM^NR^. DBU, 1, 8-diazabicycloundec-7-ene; ^t^BuOK, potasium tert-butoxide; DMSO, dimtehylsulfoxide; DCM, dichloromethane; ET_3_N, triethylamine; SO_3_Py, sulfur trioxide pyridine; Bu_4_NOH, tetrabutylammonium hydroxide; ET_3_Si, triethylsilane; THF, tetrahydrofurane; TFA, trifluoroacetic acid; rt, room temperature.

**Figure 3:**
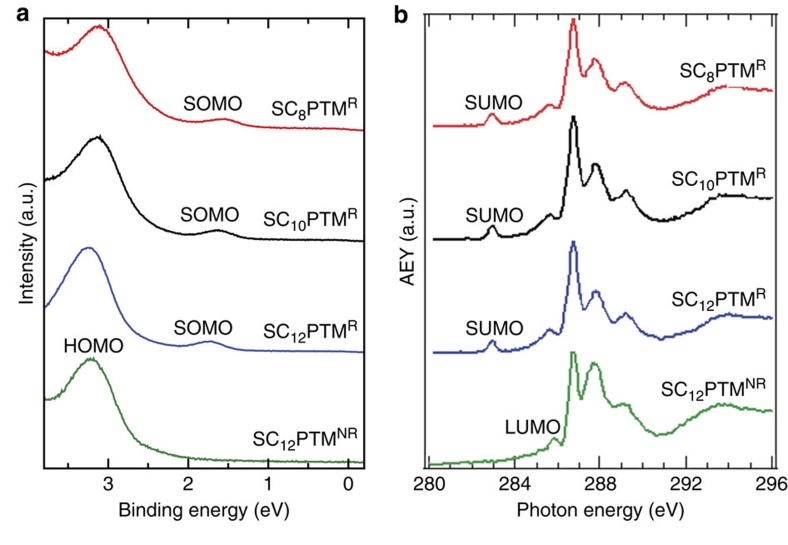
Electronic structure of the PTM^R/NR^ SAMs on Au^TS^. (**a**) Ultraviolet photoemission spectroscopy (UPS) and (**b**) C K-edge X-ray absorption fine structure (NEXAFS) spectra of SAMs derived from HSC_*n*_PTM^R^ with *n*=8, 10, 12 and HSC_12_PTM^NR^.

We note that the energy levels determined by these techniques only involve SAMs. We believe that the energy levels shift once the SAMs form a contact with the GaO_*x*_^cond^/EGaIn top-electrodes, though we believe these shifts are small due to the non-covalent nature of the SAM//GaO_*x*_^cond^/EGaIn contact. Thus, the energy levels depicted in Fig. 1 give a good qualitative indication of the energy levels. Moreover these experimental data indicate that the SUMOs of radical and LUMOs of non-radical SAMs, are closer to the *E*_F_ of GaO_*x*_/EGaIn (−4.2 eV)^44^ and SAM-modified Au^TS^ bottom electrodes (−4.1 to −4.2 eV) than the SOMO and HOMO. Therefore we believe the mechanism of charge transport involves tunnelling via the SUMO or LUMO orbitals. We note that the nature of the charge carrier cannot be directly measured in these two-terminal devices, but our results agree with the findings reported by Cahen and co-workers^45^ for *n*-alkanethiolate SAMs that showed that transport is dominated by the unoccupied molecular orbitals.

### Transport through the SAMs

**Figure 4:**
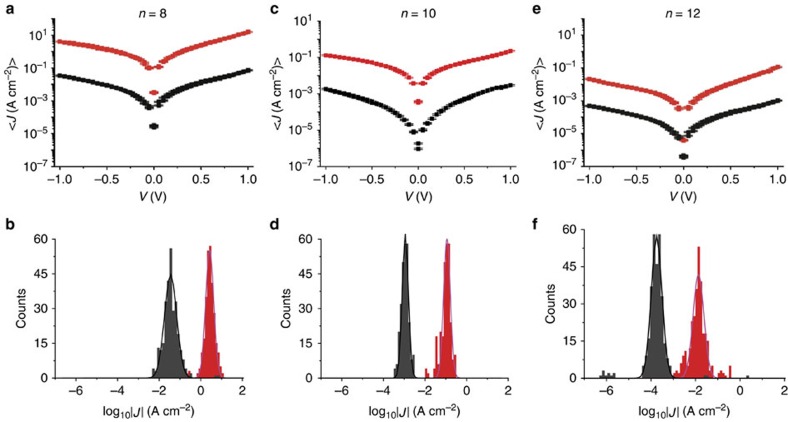
Electrical characteristics of the tunnelling junctions at room temperature. (**a**,**c**,**e**) Plots of the <log‖*J*‖>_G_ against the applied bias for radical (red) and non-radical (black) junctions and (**b**,**d**,**f**) the histograms of log‖*J*‖ measured at −1.0 V along with Gaussian fits to these histograms.

For this study, we used liquid metal GaO_*x*_^cond^/EGaIn top-electrodes since they form non-invasive soft top-contacts with the SAMs^11^,^46^,^47^. To fabricate the molecular junctions, the HSC_*n*_PTM^R/NR^ derivatives were first self-assembled on ultra-smooth Au^TS^ bottom electrodes and followed by the formation of the top-electrodes following previously reported methods (see Methods)^48^,^49^. The Au^TS^-SC_*n*_PTM^R/NR^//GaO_*x*_^cond^/EGaIn junctions were measured under the same experimental conditions and we biased the top-electrodes and grounded the bottom electrodes. We collected at least 21 traces on 20 different junctions for each type of junctions (see Table 1 for all statistics). The data were plotted in histograms of log_10_|*J*| at each applied bias (voltage steps of 0.05 V), and then we fitted Gaussians to the histograms to obtain the mean values of log_10_|*J*| (<log‖*J*‖>_G_), log-s.d. (*σ*_log_) and 95% confidence intervals. Figure 4 shows <log‖*J*‖>_G_ plotted against the applied bias with 95% confidence levels as error bars of the junctions and the histograms of log_10_|*J*| at –1.0 V for the PTM^R/NR^ SAMs (red corresponds to the radical and black to the alpha-H non-radical SAMs). In all cases, the values of <log‖*J*‖>_G_ measured for the PTM radical SAMs are consistently about 100 times higher than those values obtained for junctions with the non-radical SAMs in the whole measured bias range. The higher measured current across the radical SAMs is in agreement with our previous studies involving junctions based on cpAFM and electron transfer rate studies of different types of PTM-based SAMs^36^,^37^. Figure 4a also shows that <log‖*J*‖>_G_ values at +1.0 V are ∼1.5 times higher than at −1.0 V; this small asymmetry in the *J*(V) curves could be caused by the different electrode materials, different interfaces at top- and bottom-contact, or the negative dipole moment of the PTM molecules due to electron-withdrawing character of chlorinated phenyl rings^30^,^50^.

**Figure 5:**
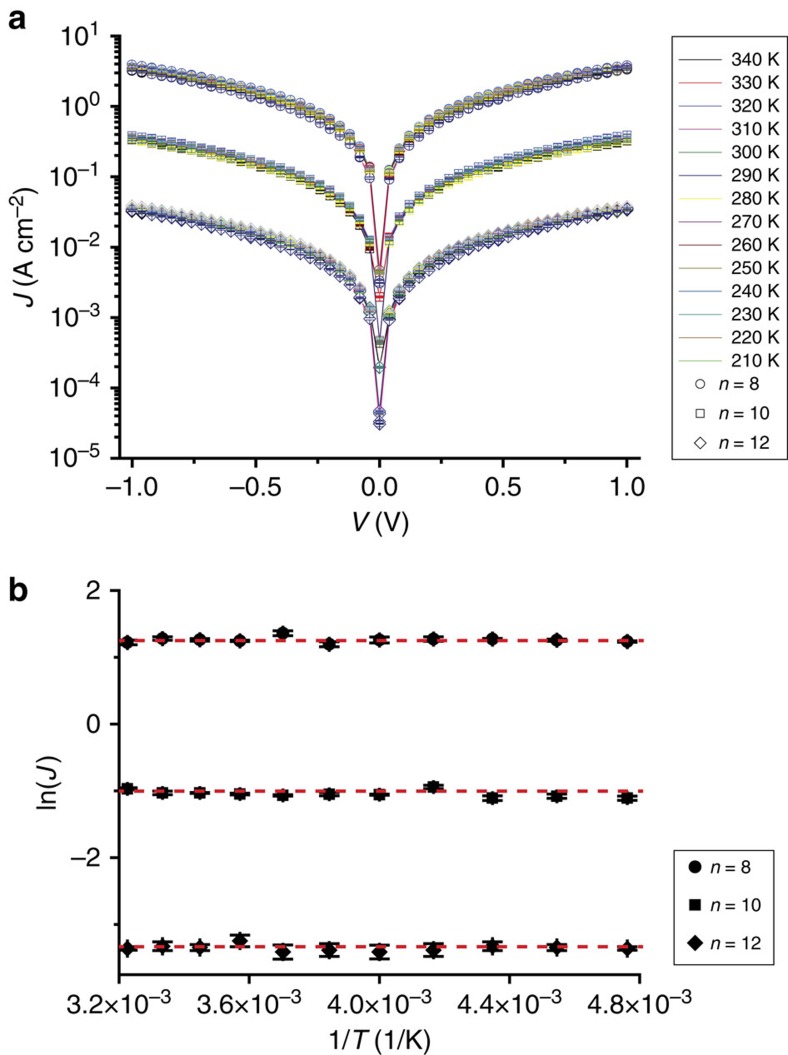
Temperature dependence measurements. (**a**) Semi-log plots of the average *J*(V) curves measured over the temperature range of 210–340 K at intervals of 10 K for Au^TS^-SC_*n*_PTM^R^ SAMs with *n*=8, 10 and 12 and (**b**) Arrhenius plots of the average *J* at −1.0 V. The error bars represent the standard deviations of 10 *J*(V) curves.

To investigate the charge transport mechanism in more detail, we carried out temperature-dependent charge transport measurements in the range of temperatures (*T*) from 340 to 210 K. We used a device made of a polymeric transparent polydimethylsiloxane (PDMS) mold having micro-channels that stabilize the GaO_*x*_^cond^/EGaIn (see ref. 51 and Methods for details). The GaO_*x*_^cond^/EGaIn formed contact with the SAM through small round-orifices present in the polymeric mold. These devices generate stable junctions over at least 1,000 traces (see Supplementary Fig. 12) and allow us to perform temperature-dependent measurements in the probestation. Figure 5a shows averaged *J*(V) curves from 10 *J*(V) traces at each temperature for the three radical SAM-based junctions, and Fig. 5b shows the Arrhenius plots for *J* at −1.0 V (see Supplementary Fig. 18 and Methods for temperature-dependent *J*(V) measurements of non-radical SAM-based junctions, that is, NRs). For the whole measured temperature range, the measured values of *J* (A cm^−2^) for the three radical SAMs are all temperature independent, which indicate the charge transport mechanism is in tunnelling regime.

**Figure 6:**
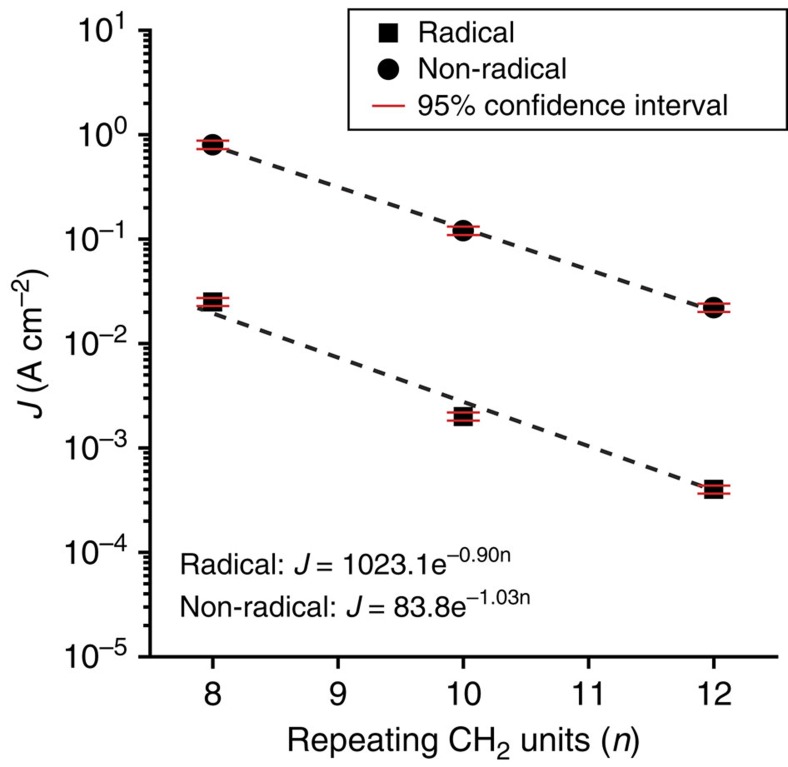
General tunnelling equation fit. The average values of *J* (A cm^−2^) at −1.0 V as a function of carbon number (*n*) for Au^TS^-SC_*n*_PTM^R/NR^//GaO_*x*_^cond^/EGaIn junctions. The dashed lines represented the fits to the general tunnelling equation. The red error bars represent the 95% confidence interval.

To investigate the dependence of *J* as a function of tunnelling distance (*d*), here we used carbon number *n* as the equivalent for *d* and plotted <log‖*J*‖>_G_ (determined at −1.0 V) versus *n* (Fig. 6). To this plot, we fitted the general tunnelling equation (equation (3)):


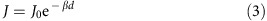


where *β* is the tunnelling decay coefficient (in *n*^−1^) and *J*_0_ (A cm^−2^) is a constant including the contact resistance and it measures the current density flowing through the electrode-SAM interface in the hypothetical case of zero separation between the electrodes^52^. The values of *β* only show a small difference for both types of junctions (*β*_R_=0.89±0.01 *n*^−1^ and a *β*_NR_=1.03±0.03 *n*^−1^) but the values of *J*_0_ differ strongly in the magnitude (log*J*_0,R_=3.0±0.2 and log*J*_0,NR_=1.9±1.2). Here, the error bar represents the 95% confidence level from least absolute deviation (LAD) fitting (see procedure of LAD fitting in ref. 26). These *β* values are similar in value to those reported for insulating organic molecules (*n*-alkanethiols), which have *β* values between 0.9 and 1.1 *n*^−1^ (refs 28, 39, 53). The magnitudes of the values of *J*_0_ are in line with other SAM-based molecular junctions but at least 1,000 times lower than single molecular junctions due to the differences in effective contact area between the techniques^47^,^54^. It was reported before that the values of *β* are proportional to the square root of the δ*E*_ME_ for coherent non-resonance tunnelling through a rectangular barrier, that is, 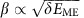 (refs 55, 56). The δ*E*_ME_ values of PTM^NR^ SAMs are consistently ∼2 times higher than PTM^R^ SAMs, which leads to about a 1.4 times increase in *β.* This back of the envelope calculation is in line with our measured data that the *β*_NR_ at −1.0 V is about 1.2 times higher than *β*_R_ (this estimation does not take into account the shape of the barrier and the renormalization of the energy levels when bias is applied). This result also suggests that the decay rate is dominated by the length of the alkyl chain and the PTM moiety is effectively decoupled from the Au electrode by the long alkyl tether. This observation is supported by temperature-dependent measurements, which demonstrate that for over the entire measured temperature range, the values of *J* are independent of the temperature for the PTM^R^ junctions. Along with the exponential decay of *J* with *d*, we believe that the mechanism of charge transport is direct tunnelling^53^,^57^. This interpretation also agrees with equation (2) and the large difference in *J*_0,R_ and *J*_0,NR_ values relates to the difference in δ*E*_ME_ values: the radical SAM-based junctions have smaller δ*E*_ME_ values and thus larger higher transmission probabilities (*T*) than the non-radical analogues.

In previous reports some of us claimed that resonant tunnelling is important where the SOMO energy determines the tunnelling barrier height. These assumptions were based on electronic structure calculations using molecules in the gas phase without accounting for Fermi-level pinning as a result of the metal–thiolate bond. The energy level diagram shown in Fig. 1 is based on experimental data (Table 1) from which we conclude that the empty levels define the tunnelling barrier height and not the filled levels. Although the exact shape of the tunnelling barrier inside the junctions during charge transport is not known, we believe that the energy-level diagram represents our system well considering the non-covalent nature of SAM//top contact. Although the junctions are asymmetric and the empty levels of the PTM^R^ moieties are energetically and spatially very close to the Fermi level of the top-electrode and separated from the bottom electrode by the alkyl, we do not observe significant current rectification. In contrast, similar junctions but with Fc or bipyridyl (BIPY) end groups do rectify^26^,^58^. These diodes rectify currents because hopping (thermally activated charge transport) involving the Fc or BIPY is important. However, redox-active SAMs with *p*-quinone end groups resulted in rectifying EGaIn junctions with low rectification ratios of 3.5 (ref. 28). Although molecular asymmetry is needed to obtain rectification, the magnitude of rectification depends on many factors including the shape of the electrostatic potential profile^48^, the terminal group coupling with the EGaIn top electrode^48^,^58^ or relaxation time of the charge carrier on the molecule (or activation energy for hopping)^16^,^59^. Currently, we do not know why certain end groups facilitate hopping while others do not.

## Discussion

In summary, a family of PTM radical (which have single-occupied molecular orbitals), and the corresponding non-radical (which have filled molecular orbitals), derivatives of different molecular lengths bearing a thiol group has been synthesized and successfully integrated in molecular junctions of the type of Au^TS^-SC_*n*_PTM^R/NR^/GaO_*x*_^cond^/EGaIn. Temperature- and chain length-dependent measurements indicate that the mechanism of charge transport across all the junctions is direct tunnelling. This work exemplifies that stable free organic radicals are interesting systems, in addition to others, in molecular junctions because they have small SOMO/SUMO or HOMO/LUMO gaps. In addition, the possibility of having the radical and non-radical junctions make it possible to re-align the molecular energy levels with respect to the Fermi-levels of the metal contacts without altering the nature of the molecule—metal contacts or the backbone of the molecule and, thus, avoiding issues such as Fermi-level pinning.

Our results demonstrate that intramolecular control over the energy-level alignment of molecular tunnelling junctions is a promising approach to control the electrical characteristics of two-terminal molecular junctions. We believe that in the future our findings may be extended to other types of junctions or to control, or perhaps induce, new electronic function.

## Methods

### Synthesis

The general procedure of the synthesis of PTM derivatives is outlined in Supplementary Fig. 1. The detailed synthesis and characterization of PTM derivatives are described in the Supplementary Methods. The representative infrared (IR), cyclic voltammetry (CV), ultraviolet–visible and electron paramagnetic resonance (EPR) spectra of radicals are shown in Supplementary Figs 2–9.

### Fabrication of template-stripped bottom-electrode

We have reported before the procedure for template stripping using epoxy (EpoTek 353ND)^44^,^49^,^60^. Briefly, we deposited A 200-nm-thick Au (with a purity of 99.999% both from Super Conductor Materials Inc) film on clean Si(100) wafers by thermal deposition (Shen Yang Ke Yi, China). The evaporation rate was about 0.3 Å s^−1^ for the first 50 nm and then increased to ∼5 Å s^−1^ to deposit the remaining 150 nm and the deposition vacuum was about 2 × 10^−6^ mbar. We cut the glass slides into pieces of 1 × 0.5 cm^2^ and then cleaned them in a solution of H_2_SO_4_:H_2_O_2_=1:5 (in volume) at 80 °C for 20 min, and then washed with H_2_O to pH 7 and drying in a stream of N_2_ gas. The glass slides were further cleaned by a plasma of air for 5 min at a pressure of 5 mbar. We dropped the epoxy on the Au surfaces and carefully added the glass slides on top of the epoxy. After the whole wafer was covered with glass slides, we cured the epoxy at 80 °C for 8 h in an oven (Epec). The final step was to lift off the glass slides with Au surfaces glued on them before immersion in the thiol solutions to minimize contamination of the Au^TS^ surfaces by air.

### Formation of the SAMs

Au^TS^ surfaces were immersed in a freshly prepared solution of 0.5 mM of each compound in toluene (HPLC grade) for 24 h at 40 °C and then for an additional 24 h at room temperature. Always, before immersing the substrates, the solution was degassed with argon. During the SAM formation, the solution was kept in dark and under argon atmosphere to avoid the decomposition of the radical species. After the time indicated above, the substrates were removed from the solution and were washed with toluene and dichloromethane to remove any physisorbed materials. The modified substrates were characterized immediately after removal from the solution. The electrochemical characterization was performed using 300 nm Au evaporated on mica from Georg Albert (Germany). Before their use, the substrates were rinsed with acetone, dichloromethane and ethanol and then exposed to ozone for 20 min. Immediately after that the substrates were immersed in ethanol (HPLC grade) for at least 30 min. Before immersing the substrates in the PTM derivative solution, they were rinsed with ethanol and dried under nitrogen stream.

### Electrochemical characterization of the SAMs

The three SAMs of Au-SC_*n*_PTM were electrochemically characterized by using CV performed with an AUTOLAB 204 with NOVA 1.9 software. We used a custom built electrochemical cell with a Pt-wire as counter electrode, Ag-wire as pseudo reference electrode and the modified Au(111) on mica as working electrode. The area exposed to the tetrabutylamonium hexafluorophosphate (TBAPF_6_) in dichloromethane electrolyte solution (0.1 M) was 0.26 cm^2^. The CVs were recorded in the range +0.1 V to −0.6 V. The electrochemical measurements were performed in a Faraday cage. CV depicted in Supplementary Fig. 10 shows the CV of the **R**_**10**_ SAM as representative for the different SAMs.

### Fabrication of PDMS top-electrodes

We have reported the fabrication of the top-electrodes of Ga_2_O_3_/EGaIn stabilized in PDMS in detail elsewhere^51^. Since the fabrication methods are essentially the same, we only provide Supplementary Fig. 11, which shows the microscope image of the through-hole filled with Ga_2_O_3_/EGaIn. A small gap of roughly 10 μm is present between the Ga_2_O_3_/EGaIn and the wall of the PDMS. In all the measurements reported here, we used electrodes with a geometrical contact area of about 7.1 × 10^2^ μm.

### Fabrication of Au^TS^-SC_*n*_PTM^R/NR^//GaO_*x*_^cond^/EGaIn junctions

The fabrication of the SAM-based junctions with cone-shaped tips of GaO_*x*_^cond^/EGaIn were reported previously^11^. Briefly, in our experiments we grounded the bottom electrode with a gold probe penetrating the SAMs and the top-electrode of GaO_*x*_^cond^/EGaIn was biased from 0 V→1.0 V→0 V→−1.0 V→0 V, with a step size of 50 mV and a delay of 0.1 s, to record the *J*(V) curves. The statistical analysis follows previous reported methods^11^ and the results are listed in Table 1.

### Stability measurement of Au^TS^-SC_8_PTM^R/NR^//GaO_*x*_^cond^/EGaIn junctions

To investigate whether the junctions are stable and do not change during voltage cycling, we prepared junctions with the PDMS top-electrodes described in Methods section `Fabrication of PDMS top-electrodes' and measured 1,000 *J*(V) traces in the bias range of ±1.0 V. All the *J*(V) traces for both types of junctions (that is, with SAMs of R_8_ (red) and NR_8_ (black)) are plotted in Supplementary Fig. 12. The shape of the *J*(V) traces did not change significantly and the values of *J* remained constant over 1,000 traces (plotted in Supplementary Fig. 12B for +1.0 V).

### Photoelectron spectroscopy

We have reported the measurement procedures and analysis of synchrotron-based photoemission spectroscopy (PES) measurements (XPS and UPS) and NEXAFS spectroscopy at the SINS (Surface, Interface and Nanostructure Science) beamline of the Singapore Synchrotron Light Source (SSLS) elsewhere^54^. Briefly, the base pressure was kept at 1 × 10^−10^ mbar. We used the Au 4*f*_7/2_ core level peak at 84.0 eV measured from a sputter-cleaned gold foil in electrical contact with the sample to calibrate the photon energy. We chose 350 eV for the Cl 2*p*, S 2*p* and C 1*s* for the XPS measurements, and 60 eV for the valence band measurements. To measure the work function, we applied –10 V bias to the sample to overcome the work function of the analyser. All UPS spectra were referenced to the Fermi edge of Au and all PES spectra were normalized by the photon current. For NEXAFS, we measured the photon energy from 270 eV to 330 eV. Two take-off angles (90° and 40°) were used to probe the angle-dependence. We performed the least-square peak fit analysis with Voigt functions (Lorentzian (30%) and Gaussian (70%)) using XPSpeak software, and the sloping background was modelled using Shirley plus linear background correction^61^,^62^. Supplementary Figs 13–16 show the high-resolution PES spectra with fits of Cl 2*p*, C 1*s* and S 2*p* of the SAMs of Au^TS^-SC_*n*_PTM^R/NR^ at two different take-off angles 90° and 40°, respectively. Supplementary Fig. 17 shows the UPS and NEXAFS spectra of Au^TS^-SC_*n*_PTM^NR^ (*n*=8 and 10).

The C 1*s* high-resolution spectra show three components: (1) C–C single bond at ∼284.2 eV labelled C_1_, (2) the central C (αC) of the PTM moiety and the C=C group at ∼285.2 to ∼285.3 eV labelled C_2_, and (3) aromatic C–Cl at ∼286.4 eV labelled C_3_ (ref. 62). The Cl 2*p* spectra show typical doublet peaks. The doublet peak of the S 2*p* spectra are solely (or mainly) contributed by the typical S–Au bond at the binding energy of 161.8 eV. The SAMs of R_8_ and NR_8_ show a small portion of low binding energy at ∼160.8 eV, which has been reported before and is assigned to chemical absorption of the SAMs at grain boundaries^62^. We summarized the fitting results of the XPS spectra of C 1*s* and Cl 2*p* in the form of the elemental ratio in Supplementary Table 2. From Supplementary Table 2, we can make three conclusions: (1) the decrease of C_1_/C_3_ ratios from carbon number 8 to 12 indicate the SAMs are standing up; (2) the C_2_/C_3_ ratios are constant at 90° emission but slightly decrease at 40° due to the attenuation of the first C connected to the PTM group and (3) the Cl/C_3_ ratios are constant for different SAMs and the difference between 90° and 40° emission angle is caused by the large attention of the central C signal compared with the Cl atom.

### Determination of the surface coverage of the SAMs from the S 2*p* spectra

To calculate the surface coverage of the PTM^R/NR^ SAMs, we determined the integrated intensity of Cl 2*p* spectra (*I*_Cl_) of the PTM^R/NR^ and S(CH_2_)_10_CH_2_Cl (SC_11_Cl) SAMs (listed in the Supplementary Table 1). Since the Cl atoms were connected to the terminal groups of the SAMs, the *I*_Cl_ can be related to the surface coverage of the SAMs. The relative surface coverage was calculated by comparing the *I*_Cl_ of the PTM^R/NR^ SAMs, which divided by 14 (PTM^R/NR^ SAMs contains 14 Cl atoms), against that of SC_11_Cl SAMs. The surface coverage of SC_11_Cl SAMs on Ag has been reported before and is 1.1 × 10^−9^ mol cm^−2^ (ref. 29). Thus, we compared the values of *I*_Cl_ of the PTM^R/NR^ SAMs against those of the SC_11_Cl SAMs to calculate the relative surface coverage of the PTM^R/NR^ SAMs (Supplementary Table 1). We estimated the uncertainties to be about 5% from the fitting errors of Cl 2*p* spectra. However, we note that not all of the 14 Cl atoms on the PTM moiety are located at the top of the SAMs, and the signal of at least 4 Cl atoms (connected to the phenyl ring with alkyl chain) are attenuated by half of the length (∼6.3 Å) of the PTM moiety. Thus, the surface coverage calculated by XPS here was underestimated by at least 13% (=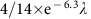). Nevertheless, the relative surface coverages are the same for all the PTM SAMs which support our conclusion that we did not change the supramolecular structure of the PTM^R/NR^ SAMs.

We also estimated the theoretical surface coverage of these SAMs formed on the Au(111) surface, and the dimension of the PTM moiety was calculated from CPK model. Since the PTM moiety is a planer-triangle, its projection on the *x–y* plane changes between rectangles (or a parallelogram dependent on the molecular tilt angle which we do not know). We estimated a surface coverage of 1.8 × 10^−10^ mol cm^−2^ from the simple case of rectangle projection (side lengths: *a*=12.6 Å and *b*=7.4 Å). The surface coverage estimated from XPS is similar to the theoretical estimation.

### Determination of the thickness of the SAMs from the S 2*p* spectra

We have reported the methods to fit the spectra and to calculate the SAM thickness from angle-dependent XPS measurements in ref. 48. We only give a short description here. The SAM thickness (*d*, nm) can be expressed as the sum of the Au–S bond (*d*_Au–S_=1.8 Å)^63^ and the over layer thickness (the distance from the middle of the S atom to vacuum) *d*_2_ in equation (4)


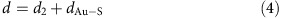


The different angles of the incident light (*γ*) result in different emission area on the samples. To normalize the footprint of incident light, we calculate the effective intensity (*I*_*θ*_)


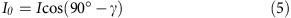


where *I* is the integrated intensity of the peak. The values of *I*_*θ*_ are exponentially dependent on *d*_2_ and on the take-off angle


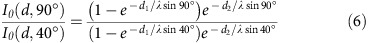


where the *d*_1_ (=1.5 Å) is estimated from the radius of S atoms and the S–C bond, and *λ* (=8 Å) is the inelastic mean free path^62^. We can rewrite the equation (6) to calculate the value of *d*_2_


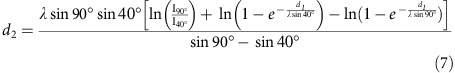


The S 2*p* spectra along with fits are shown in Supplementary Figs 12–15, and the results are listed in Supplementary Table 3. The uncertainty of ±2 Å takes into account the fitting errors and the angular misalignment due to sample mounting.

### Calculation of LUMO energy level from NEXAFS spectra

We determined the SUMO or LUMO level with respect to the Fermi level of the electrode by the binding energy of the C 1*s* core level of the PTM from the XPS spectra (Supplementary Figs 13–18) and the SUMO or LUMO peak from the NEXAFS spectra with a correction of 0.5 eV for core–hole exciton-binding energy to take into account the core–hole attraction effect. Others have reported correction factors in the range of 0.1–2.0 eV (ref. 64), but we have used 0.5 eV also for other types of SAMs as described in the supporting information of ref. 60. Since we used for both types of junctions with NR and R SAMs the same correction factor, the energy levels for both types of junctions would shift equally in case one would chose different correction factors and hence will not affect our conclusions.

### Temperature-dependent measurement of a junction with a SAM of NR_8_

We used EGaIn top-electrodes stabilized in a through-hole in PDMS to form stable junctions, and measured the temperature-dependent *J*(V) in a probe station as mentioned in the main text. Supplementary Fig. 18 shows the *J*(V) traces recorded at 250–330 K at intervals of 10 K. Both the Arrhenius plots and the *J*(V) traces show that the junction characteristics are independent of temperature as expected, since the non-radical SAMs have larger molecular energy gaps than their radical counterparts and behave as tunnelling barrier.

### LAD fitting

As mentioned in the manuscript, we performed LAD fitting of the tunnelling equation (equation (3) in the main text) to the full data set of log_10_|*J*| at different applied bias (*V*) from −1.0 V to −0.1 V. The details of the LAD fitting have been reported in ref. 65. Supplementary Fig. 19 shows the plot of the tunnelling decay coefficient (*β*) against *V*. We found that the value of *β* of the junctions with SAMs of PTM^NR^ is consistently ∼1.2 times higher than that of PTM^R^.

### Data availability

The data that support the findings of this study are available from the corresponding author upon request.

## Additional information

**How to cite this article:** Yuan, L. *et al*. Chemical control over the energy-level alignment in a two-terminal junction. *Nat. Commun.* 7:12066 doi: 10.1038/ncomms12066 (2016).

## Supplementary Material

Supplementary Information Supplementary Figures 1-19, Supplementary Tables 1-3 and Supplementary Methods.

## Figures and Tables

**Table 1:** Statistics of electrical characterization of the PTM-based junctions and energy level determination by UPS and NEXAFS spectroscopy at room temperature.

SAM	Number of junctions	Traces	Short*	Yield (%)†	*d* (nm)‡	Work function (eV)§	SOMO/HOMO (eV)§	SUMO/LUMO (eV)||	Energy gap (eV)¶	δ*E*_ME_ (eV)#
R_8_	20	424	2	90	1.83	4.12±0.05	−5.65±0.02	−3.72±0.10	1.93±0.12	0.40±0.15
R_10_	20	417	3	85	2.05	4.15±0.05	−5.69±0.02	−3.65±0.10	2.04±0.12	0.50±0.15
R_12_	20	424	2	90	2.20	4.20±0.05	−5.72±0.02	−3.51±0.10	2.21±0.12	0.69±0.15
NR_8_	20	420	2	90	1.79	4.28±0.05	−6.95±0.02	−3.08±0.10	3.87±0.12	1.20±0.15
NR_10_	20	420	2	90	2.08	4.26±0.05	−7.02±0.02	−3.00±0.10	4.02±0.12	1.26±0.15
NR_12_	20	424	1	95	2.22	4.26±0.05	−6.95±0.02	−2.86±0.10	4.09±0.12	1.40±0.15

ARXPS, angle-resolved X-ray photoelectron spectroscopy; HOMO, highest-occupied molecular orbital; LUMO, lowest occupied molecular orbital; NEXAFS, near edge X-ray adsorption fine structure spectroscopy; NR, non-radical; SAM, self-assembled monolayer; SOMO, single occupied molecular orbital; SUMO, single unoccupied molecular orbital.

^*^A junction short was defined when the value of *J* exceeded 10^2^ A cm^−2^ (the upper limit of *J* measurable by our instrument) while recording 20 *J*(V) scans.

^†^The yield of non-shorting junctions is defined as the percentage number of non-shorting junctions divided by the total number of junctions.

^‡^The thickness *d* was determined by ARXPS. The error bars were 0.2 nm (see Methods for details).

^§^The work function and the energy of the SOMO/HOMO were determined by UPS (see Methods for details).

^||^The energy level of SUMO/LUMO was determined by NEXAFS spectroscopy (see Methods for details).

^¶^The energy gap was calculated by the difference between the HOMO and LUMO for the NR SAM and SOMO and SUMO for the R SAM.

^#^The value of δ*E*_ME_ was calculated by the difference between the work function and SUMO/LUMO.

## References

[b1] Akkerman H. B. & de Boer B. Electrical conduction through single molecules and self-assembled monolayers. J. Phys. Condens. Matter 20, 013001 (2008).

[b2] Aviram A. & Ratner M. Molecular rectifiers. Chem. Phys. Lett. 29, 277–283 (1974).

[b3] Cui X. D. . Making electrical contacts to molecular monolayers. Nanotechnology 13, 5–14 (2002).

[b4] Heimel G., Romaner L., Zojer E. & Bredas J.-L. The interface energetics of self-assembled monolayers on metals. Acc. Chem. Res. 41, 721–729 (2008). 18507404 10.1021/ar700284q

[b5] Malen J. A. . Identifying the length dependence of orbital alignment and contact coupling in molecular heterojunctions. Nano Lett. 9, 1164–1169 (2009). 19239204 10.1021/nl803814f

[b6] Coughlin J. E., Henson Z. B., Welch G. C. & Bazan G. C. Design and synthesis of molecular donors for solution-processed high-efficiency organic solar cells. Acc. Chem. Res. 47, 257–270 (2014). 23984626 10.1021/ar400136b

[b7] Hung L. S. & Chen C. H. Recent progress of molecular organic electroluminescent materials and devices. Mater. Sci. Eng. R 39, 143–222 (2002).

[b8] Scott J. C. Metal-organic interface and charge injection in organic electronic devices. J. Vac. Sci. Technol. A 21, 521–531 (2003).

[b9] Mativetsky J. M. . Azobenzenes as light-controlled molecular electronic switches in nanoscale metal-molecule-metal junctions. J. Am. Chem. Soc. 130, 9192–9193 (2008). 18576645 10.1021/ja8018093

[b10] Liu Y. . Giant enhancement in vertical conductivity of stacked CVD graphene sheets by self-assembled molecular layers. Nat. Commun. 5, 5461 (2014). 25410480 10.1038/ncomms6461

[b11] Nerngchamnong N. . The role of van der Waals forces in the performance of molecular diodes. Nat. Nanotechnol. 8, 113–118 (2013). 23292010 10.1038/nnano.2012.238

[b12] Sayed S. Y., Fereiro J. A., Yan H., McCreery R. L. & Bergren A. J. Charge transport in molecular electronic junctions: Compression of the molecular tunnel barrier in the strong coupling regime. Proc. Natl Acad. Sci. USA 109, 11498–11503 (2012). 22660930 10.1073/pnas.1201557109 PMC3406855

[b13] Yoon H. J., Bowers C. M., Baghbanzadeh M. & Whitesides G. M. The rate of charge tunnelling is insensitive to polar terminal groups in self-assembled monolayers in Ag^TS^S(CH_2_)_n_M(CH_2_)_m_T//Ga_2_O_3_/EGaIn Junctions. J. Am. Chem. Soc. 136, 16–19 (2014). 24350722 10.1021/ja409771u

[b14] Liao K.-C., Yoon H. J., Bowers C. M., Simeone F. C. & Whitesides G. M. Replacing Ag^TS^SCH_2_-R with Ag^TS^O_2_C-R in EGaIn-based tunnelling junctions does not significantly change rates of charge transport. Angew. Chem. Int. Ed. 53, 3889–3893 (2014). 10.1002/anie.201308472 24596177

[b15] Yoon H. J. . The rate of charge tunnelling through self-assembled monolayers is insensitive to many functional group substitutions. Angew. Chem. Int. Ed. 51, 4658–4661 (2012). 10.1002/anie.201201448 PMC3518389 22504880

[b16] Joachim C. & Ratner M. A. Molecular electronics: some views on transport junctions and beyond. Proc. Natl Acad. Sci. USA 102, 8801–8808 (2005). 15956192 10.1073/pnas.0500075102 PMC1157019

[b17] Landauer R. Spatial variation of currents and fields due to localized scatterers in metallic conduction (and comment). J. Math. Phys. 37, 5259–5268 (1996).

[b18] Datta S. Lessons from Nanoelectronics: A New Perspective on Transport Vol. 1, World Scientific Publishing Co. Pte. Ltd. (2012).

[b19] Díez-Pérez I. . Rectification and stability of a single molecular diode with controlled orientation. Nat. Chem. 1, 635–641 (2009). 21378955 10.1038/nchem.392

[b20] Frisenda R. . Kondo effect in a neutral and stable all organic radical single molecule break junction. Nano Lett. 15, 3109–3114 (2015). 25897770 10.1021/acs.nanolett.5b00155

[b21] Fave C. . Tunable electrochemical switches based on ultrathin organic films. J. Am. Chem. Soc. 129, 1890–1891 (2007). 17253692 10.1021/ja068143u

[b22] Capozzi B. . Tunable charge transport in single-molecule junctions via electrolytic gating. Nano Lett. 14, 1400–1404 (2014). 24490721 10.1021/nl404459q

[b23] Lenfant S. . Electron transport through rectifying self-assembled monolayer diodes on silicon: Fermi-level pinning at the molecule−metal interface. J. Phys. Chem. B 110, 13947–13958 (2006). 16836346 10.1021/jp053510u

[b24] Kim B., Choi S. H., Zhu X. Y. & Frisbie C. D. Molecular tunnel junctions based on pi-conjugated oligoacene thiols and dithiols between Ag, Au, and Pt contacts: Effect of surface linking group and metal work function. J. Am. Chem. Soc. 133, 19864–19877 (2011). 22017173 10.1021/ja207751w

[b25] de Boer B., Hadipour A., Mandoc M. M., van Woudenbergh T. & Blom P. W. M. Tuning of metal work functions with self-assembled monolayers. Adv. Mater. 17, 621–625 (2005).

[b26] Yuan L., Thompson D., Cao L., Nerngchamnong N. & Nijhuis C. A. One carbon matters: The origin and reversal of odd–even effects in molecular diodes with self-assembled monolayers of ferrocenyl-alkanethiolates. J. Phys. Chem. C 119, 17910–17919 (2015).

[b27] Nijhuis C. A., Reus W. F. & Whitesides G. M. Molecular rectification in metal-SAM-metal oxide-metal junctions. J. Am. Chem. Soc. 131, 17814–17827 (2009). 19928851 10.1021/ja9048898

[b28] Reus W. F., Thuo M. M., Shapiro N. D., Nijhuis C. A. & Whitesides G. M. The SAM, Not the electrodes, dominates charge transport in metal-monolayer//Ga_2_O_3_/gallium-indium eutectic junctions. ACS Nano 6, 4806–4822 (2012). 22548354 10.1021/nn205089u

[b29] Wang D. . Tuning the tunnelling rate and dielectric response of sam-based junctions via a single polarizable atom. Adv. Mater. 27, 6689–6695 (2015). 26414779 10.1002/adma.201502968

[b30] Crivillers N., Mas-Torrent M., Vidal-Gancedo J., Veciana J. & Rovira C. Self-assembled monolayers of electroactive polychlorotriphenylmethyl radicals on Au(111). J. Am. Chem. Soc. 130, 5499–5506 (2008). 18376836 10.1021/ja710845v

[b31] Simão C., Mas-Torrent M., Veciana J. & Rovira C. Multichannel molecular switch with a surface-confined electroactive radical exhibiting tunable wetting properties. Nano Lett. 11, 4382–4385 (2011). 21875097 10.1021/nl2025097

[b32] Crivillers N. . Self-assembled monolayers of a multifunctional organic radical. Angew. Chem. Int. Ed. 46, 2215–2219 (2007). 10.1002/anie.200603599 17310480

[b33] Simão C. . A robust molecular platform for non-volatile memory devices with optical and magnetic responses. Nat. Chem. 3, 359–364 (2011). 21505493 10.1038/nchem.1013

[b34] Frisneda R. . Kondo effect in a neutral and stable all organic radical single molecule break junction. Nano Lett. 15, 3109–3114 (2015). 25897770 10.1021/acs.nanolett.5b00155

[b35] Ballester M., Riera-Figueras J., Castañer J., Badfa C. & Monso J. M. Inert carbon free radicals. I. Perchlorodiphenylmethyl and perchlorotriphenylmethyl radical series. J. Am. Chem. Soc. 93, 2215–2225 (1971).

[b36] Crivillers N. . Dramatic influence of the electronic structure on the conductivity through open- and closed-shell molecules. Adv. Mater. 21, 1177–1181 (2009).

[b37] Crivillers N. . Negative differential resistance (NDR) in similar molecules with distinct redox behaviour. Chem. Commun. 47, 4664–4666 (2011). 10.1039/c1cc10677e 21409270

[b38] Rovira C. . Influence of topology on the long-range electron-transfer phenomenon. Chem. Eur. J. 7, 240–250 (2001). 11205016 10.1002/1521-3765(20010105)7:1<240::aid-chem240>3.0.co;2-h

[b39] Yuan L., Jiang L., Zhang B. & Nijhuis C. A. Dependency of the tunnelling decay coefficient in molecular tunnelling junctions on the topography of the bottom electrodes. Angew. Chem. Int. Ed. 53, 3377–3381 (2014). 10.1002/anie.201309506 24615875

[b40] Weiss E. A. . Influence of defects on the electrical characteristics of mercury-drop junctions: Self-assembled monolayers of n-alkanethiolates on rough and smooth silver. J. Am. Chem. Soc. 129, 4336–4349 (2007). 17358061 10.1021/ja0677261

[b41] Weiss E. A. . Si/SiO_2_-Templated formation of ultraflat metal surfaces on glass, polymer, and solder supports: Their use as substrates for self-assembled monolayers. Langmuir 23, 9686–9694 (2007). 17696377 10.1021/la701919r

[b42] Shekhah O. . Grafting of monocarboxylic substituted polychlorotriphenylmethyl radicals onto a COOH-functionalized self-assembled monolayer through copper (II) metal ions. Langmuir 24, 6640–6648 (2008). 18522443 10.1021/la800771q

[b43] Mugnaini V. . Looking inside the perchlorinated trityl radical/metal spinterface through spectroscopy. J. Phys. Chem. Lett. 6, 2101–2106 (2015). 26266509 10.1021/acs.jpclett.5b00848

[b44] Wimbush K. S. . Bias induced transition from an ohmic to a non-ohmic interface in supramolecular tunnelling junctions with Ga_2_O_3_/EGaIn top electrodes. Nanoscale 6, 11246–11258 (2014). 25132523 10.1039/c4nr02933j

[b45] Yaffe O. . Charge transport across metal/molecular (alkyl) monolayer-Si junctions is dominated by the LUMO level. Phys. Rev. B 85, 045433 (2012).

[b46] Chiechi R. C., Weiss E. A., Dickey M. D. & Whitesides G. M. Eutectic gallium–indium (EGaIn): a moldable liquid metal for electrical characterization of self-assembled monolayers. Angew. Chem. Int. Ed. 47, 142–144 (2008). 10.1002/anie.200703642 18038438

[b47] Simeone F. C. . Defining the value of injection current and effective electrical contact area for egain-based molecular tunnelling junctions. J. Am. Chem. Soc. 135, 18131–18144 (2013). 24187999 10.1021/ja408652h

[b48] Yuan L. . Controlling the direction of rectification in a molecular diode. Nat. Commun. 6, 7324 (2015). 25727708 10.1038/ncomms7324

[b49] Yuan L., Jiang L., Thompson D. & Nijhuis C. A. On the remarkable role of surface topography of the bottom-electrodes in blocking leakage currents in molecular diodes. J. Am. Chem. Soc. 136, 6554–6557 (2014). 24738478 10.1021/ja5007417

[b50] Nijhuis C. A., Reus W. F., Barber J. R. & Whitesides G. M. Comparison of SAM-based junctions with Ga_2_O_3_/EGaln top electrodes to other large-area tunnelling junctions. J. Phys. Chem. C 116, 14139–14150 (2012).

[b51] Wan A., Jiang L., Sangeeth C. S. S. & Nijhuis C. A. Reversible soft top-contacts to yield molecular junctions with precise and reproducible electrical characteristics. Adv. Funct. Mater. 24, 4442–4456 (2014).

[b52] Nijhuis C. A., Reus W. F. & Whitesides G. M. Mechanism of rectification in tunnelling junctions based on molecules with asymmetric potential drops. J. Am. Chem. Soc. 132, 18386–18401 (2010). 21126089 10.1021/ja108311j

[b53] Akkerman H. B. . Electron tunnelling through alkanedithiol self-assembled monolayers in large-area molecular junctions. Proc. Natl Acad. Sci. USA 104, 11161–11166 (2007). 17592120 10.1073/pnas.0701472104 PMC1899190

[b54] Jiang L., Sangeeth C. S. S., Wan A., Vilan A. & Nijhuis C. A. Defect scaling with contact area in egain-based junctions: impact on quality, joule heating, and apparent injection current. J. Phys. Chem. C 119, 960–969 (2015).

[b55] Simmons J. G. Generalized formula for electric tunnel effect between similar electrodes separated by a thin insulating film. J. Appl. Phys. 34, 1793–1803 (1963).

[b56] Salomon A. . Comparison of electronic transport measurements on organic molecules. Adv. Mater. 15, 1881–1890 (2003).

[b57] Choi S. H., Kim B. S. & Frisbie C. D. Electrical resistance of long conjugated molecular wires. Science 320, 1482–1486 (2008). 18556556 10.1126/science.1156538

[b58] Yoon H. J. . Rectification in tunnelling junctions: 2,2'-bipyridyl-terminated n-alkanethiolates. J. Am. Chem. Soc. 136, 17155–17162 (2014). 25389953 10.1021/ja509110a

[b59] Moth-Poulsen K. & Bjørnholm T. Molecular electronics with single molecules in solid-state devices. Nat. Nanotechnol. 4, 551–556 (2009). 19734925 10.1038/nnano.2009.176

[b60] Yuan L., Breuer R., Jiang L., Schmittel M. & Nijhuis C. A. A molecular diode with a statistically robust rectification ratio of three orders of magnitude. Nano Lett. 15, 5506–5512 (2015). 26196854 10.1021/acs.nanolett.5b02014

[b61] Tour J. M. . Self-assembled monolayers and multilayers of conjugated thiols, alpha,omega-dithiols, and thioacetyl-containing adsorbates - understanding attachments between potential molecular wires and gold surfaces. J. Am. Chem. Soc. 117, 9529–9534 (1995).

[b62] Ishida T. . High-resolution X-ray photoelectron spectra of organosulfur monolayers on Au(111): S(2p) spectral dependence on molecular species. Langmuir 15, 6799–6806 (1999).

[b63] Watcharinyanon S. . Molecular orientation of thiol-derivatized tetraphenylporphyrin on gold studied by XPS and NEXAFS. Surf. Sci. 603, 1026–1033 (2009).

[b64] Stohr J. *NEXAFS Spectroscopy* (Springer–Verlag, (1992).

[b65] Reus W. F. . Statistical tools for analyzing measurements of charge transport. J. Phys. Chem. C 116, 6714–6733 (2010).

