# Supplementary material for: Chemical control over the energy-level alignment in a two-terminal junction
Source: Nat Commun. 2016 Jul 26;7:12066. doi: 10.1038/ncomms12066 (PMC4963472; doi:10.1038/ncomms12066)
Supplement: Supplementary Information — Supplementary Figures 1-19, Supplementary Tables 1-3 and Supplementary Methods. [file ncomms12066-s1.pdf]

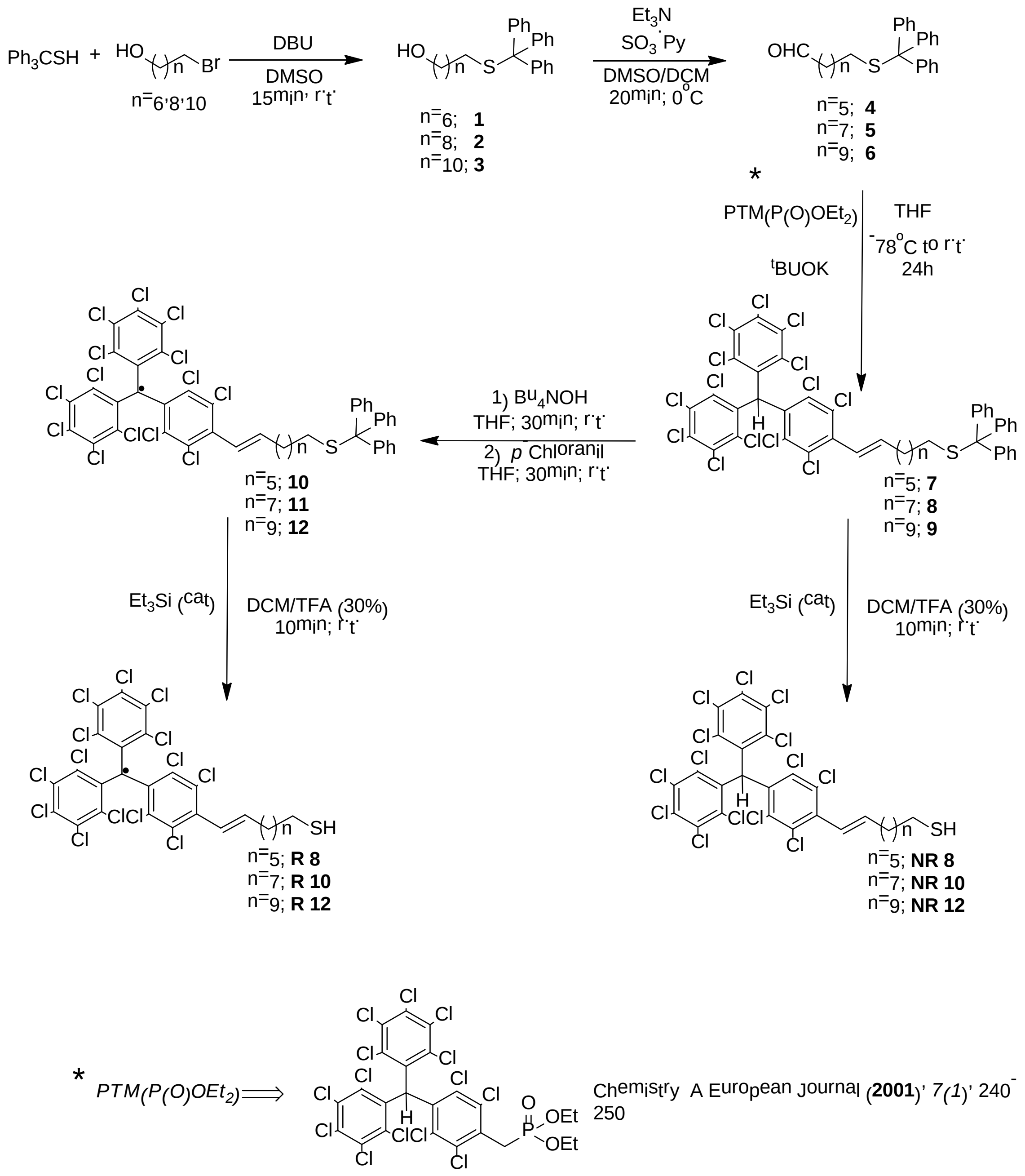

Supplementary Figure 1. General procedure for the synthesis of PTMC$_x$STrityl and PTMC$_x$SH.

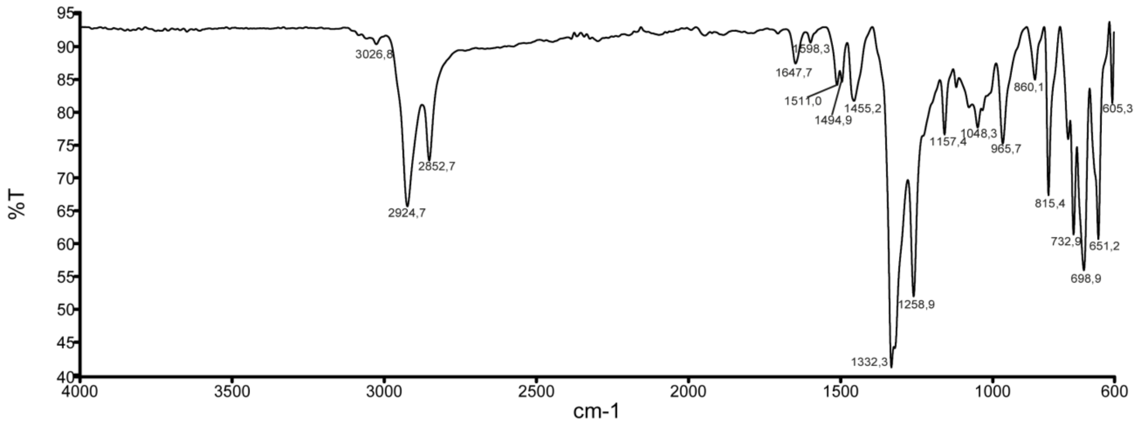

Supplementary Figure 2. IR-ATR spectrum of compound **R10**.

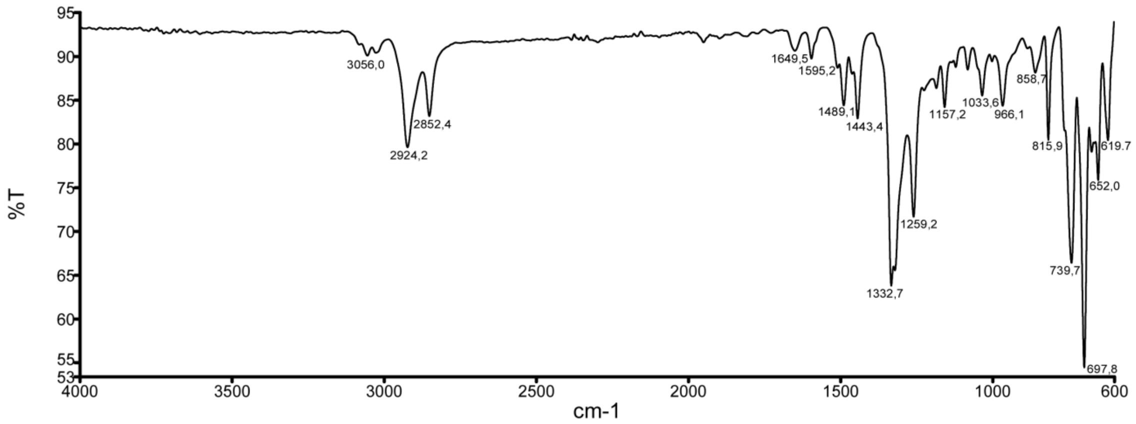

Supplementary Figure 3. IR-ATR spectrum of compound **11**.

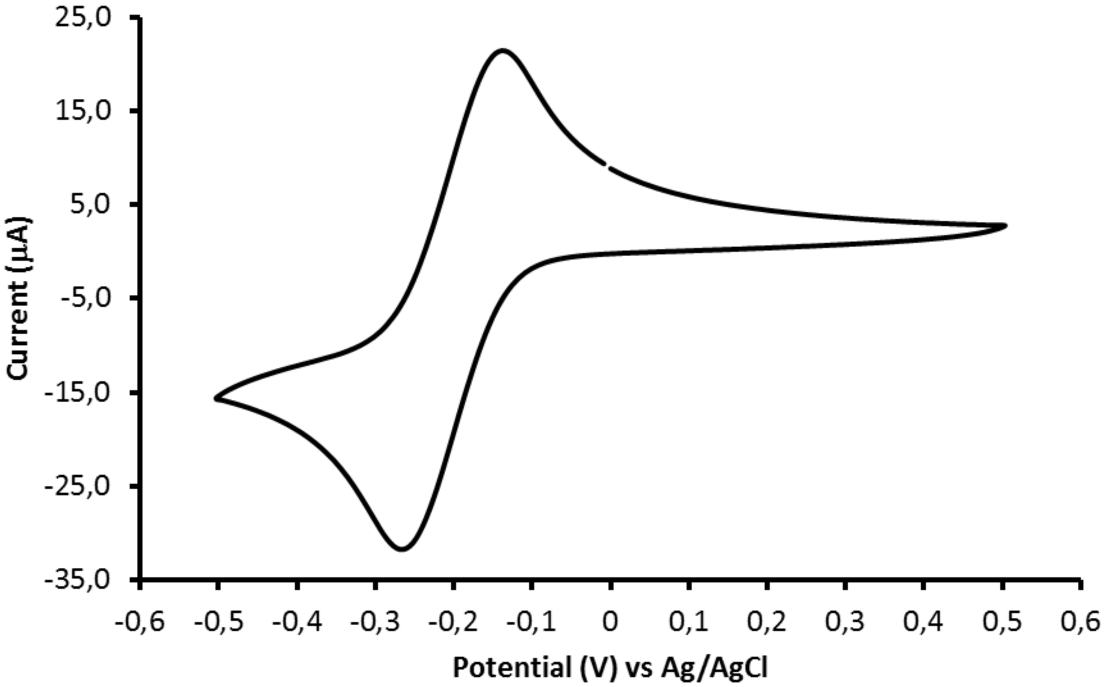

Supplementary Figure 4. Cyclic voltammogram of compound **R8.**

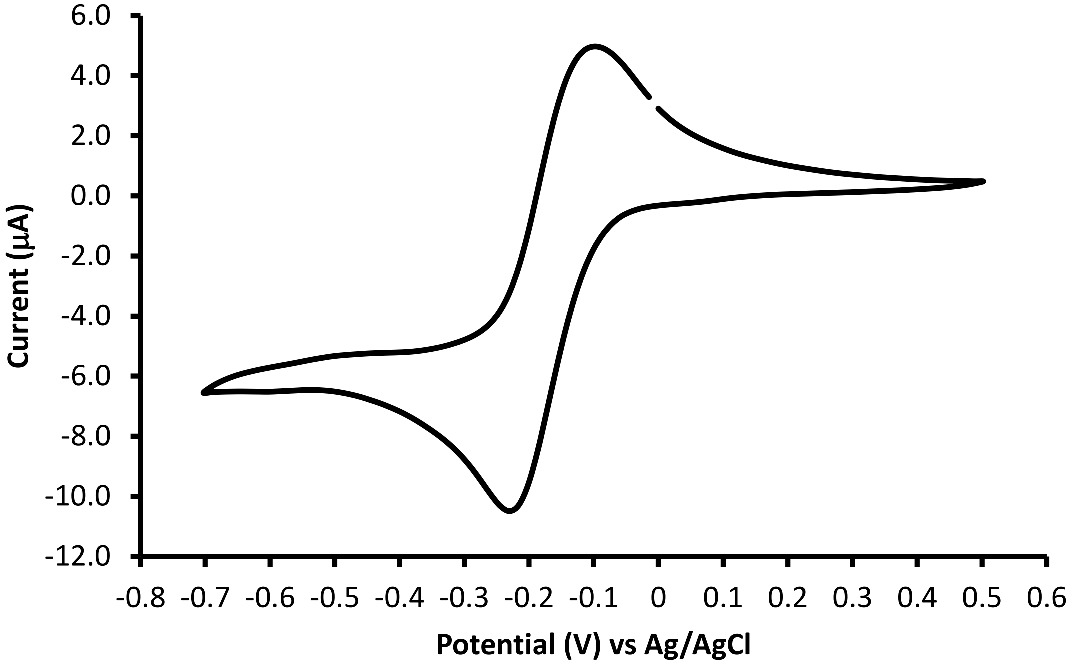

Supplementary Figure 5. Cyclic voltammogram of compound **11**.

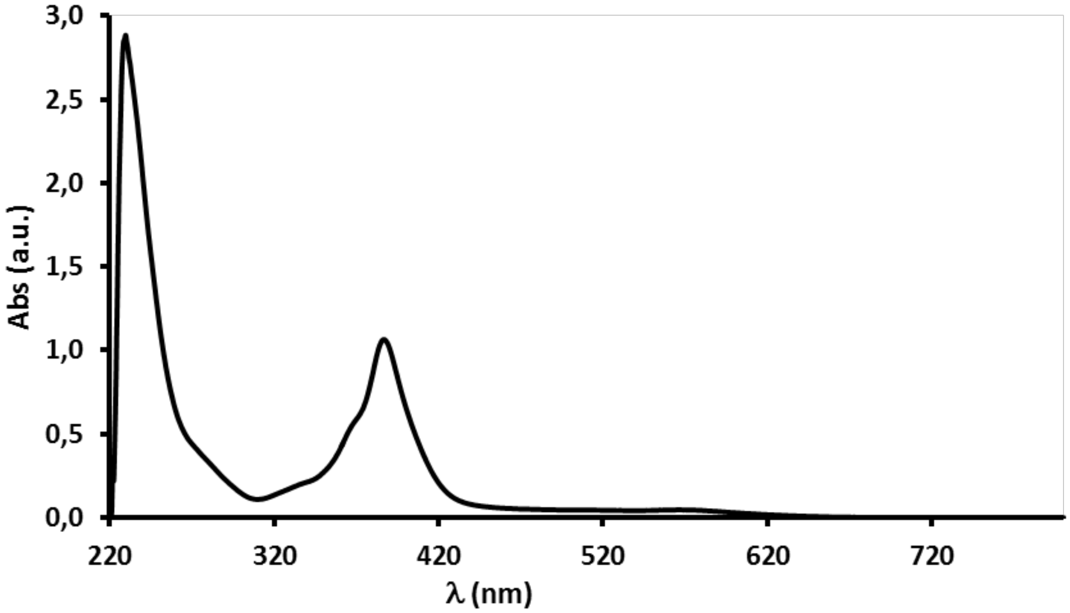

Supplementary Figure 6. UV-Vis spectrum of compound **10** in CH_2Cl_2 solution.

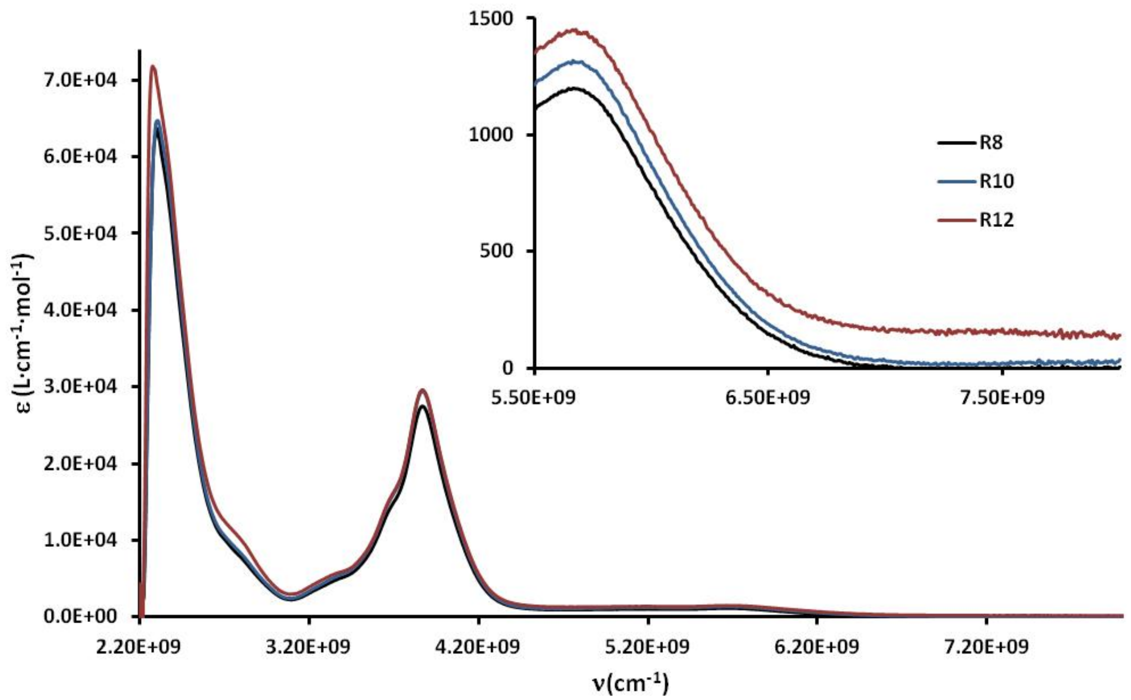

Supplementary Figure 7. UV-Vis spectra of radicals **R8**, **R10** and **R12** in CH_2Cl_2.

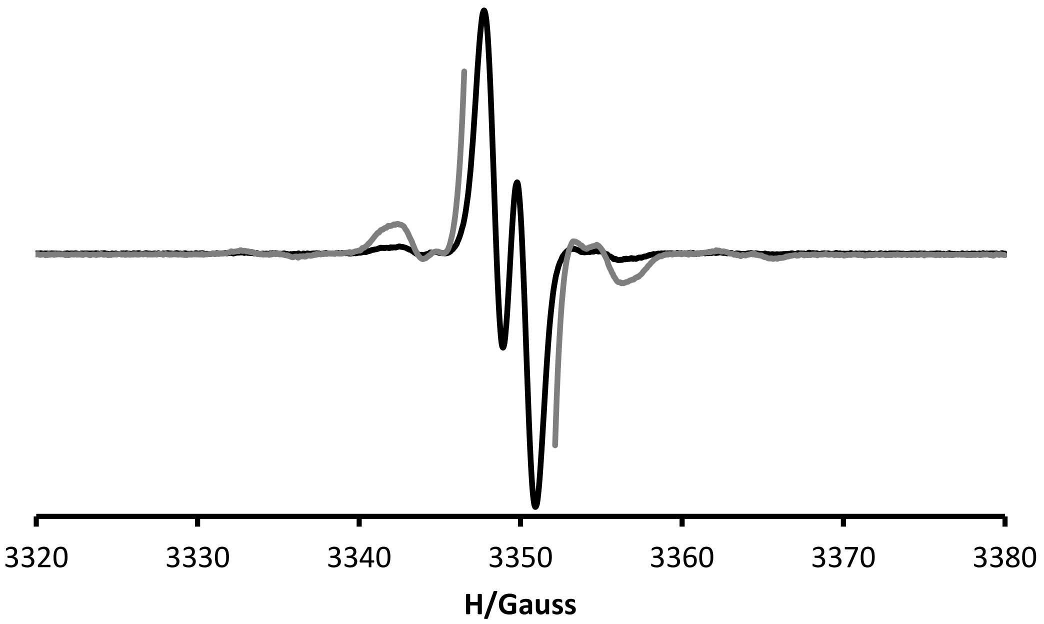

Supplementary Figure 8. EPR spectrum of compound **R10** in CH_2Cl_2 solution, at room temperature.

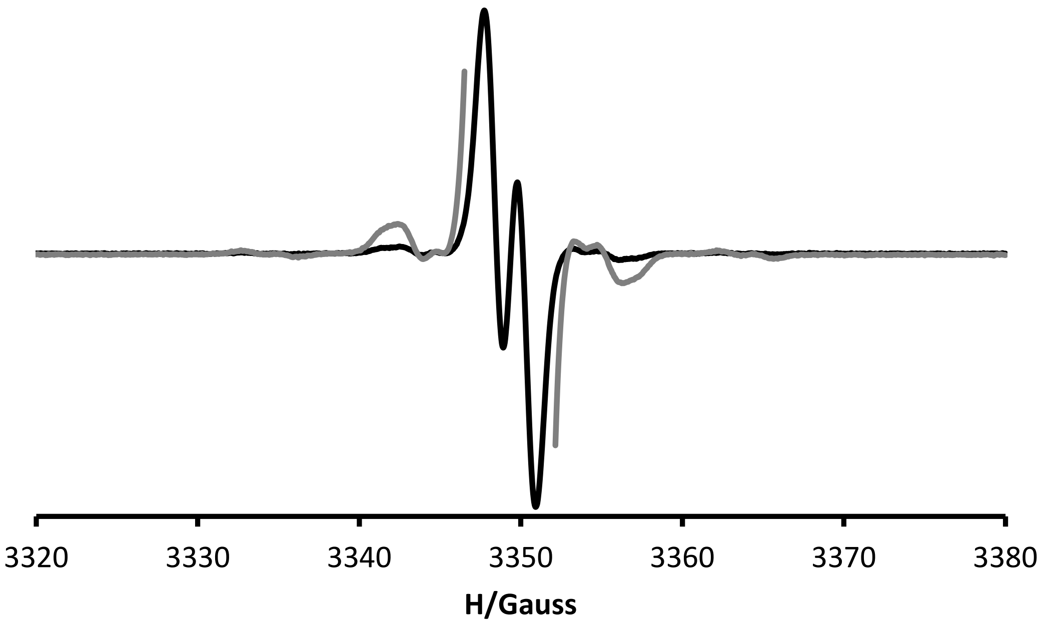

Supplementary Figure 9. EPR spectrum of compound **10** in CH_2Cl_2 solution, at room temperature.

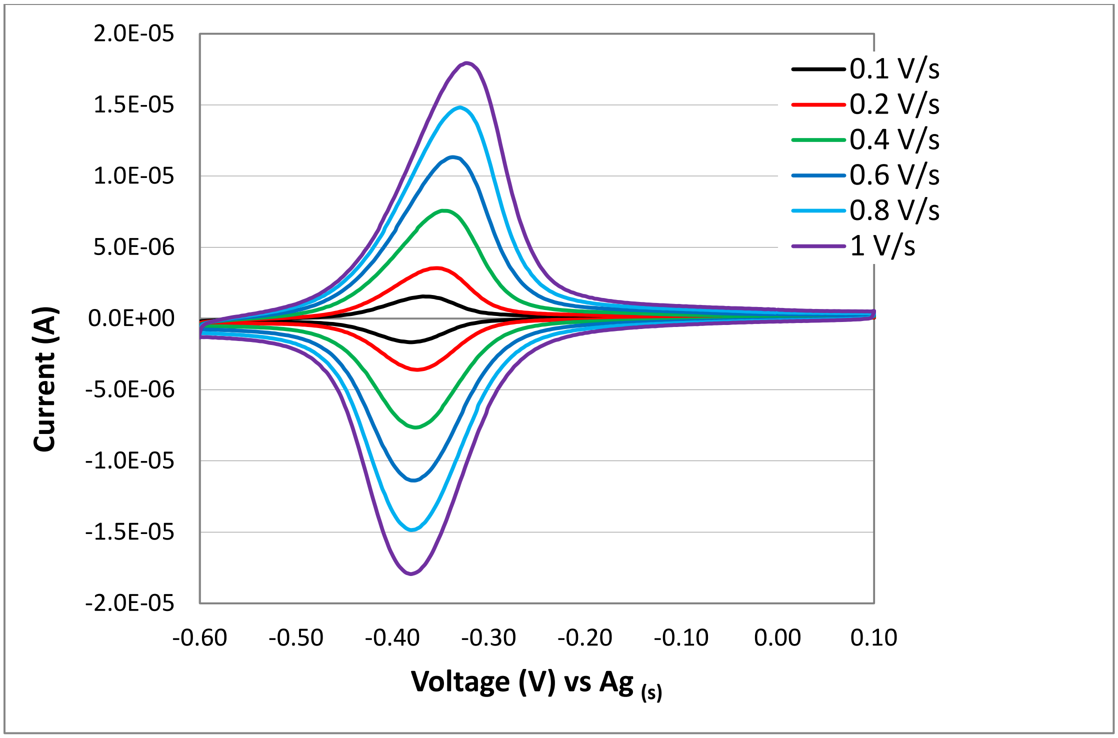

Supplementary Figure 10. Cyclic voltammogram of the $\mathbf{R_{10}}$ SAM on Au (111)/mica.

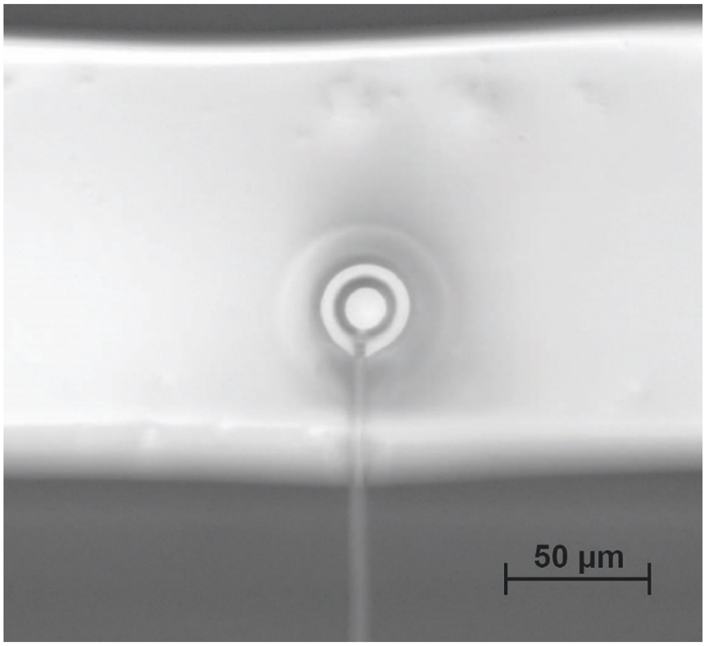

Supplementary Figure 11. Optical microscope image of the through-hole filled with Ga_2O_3/EGaIn.

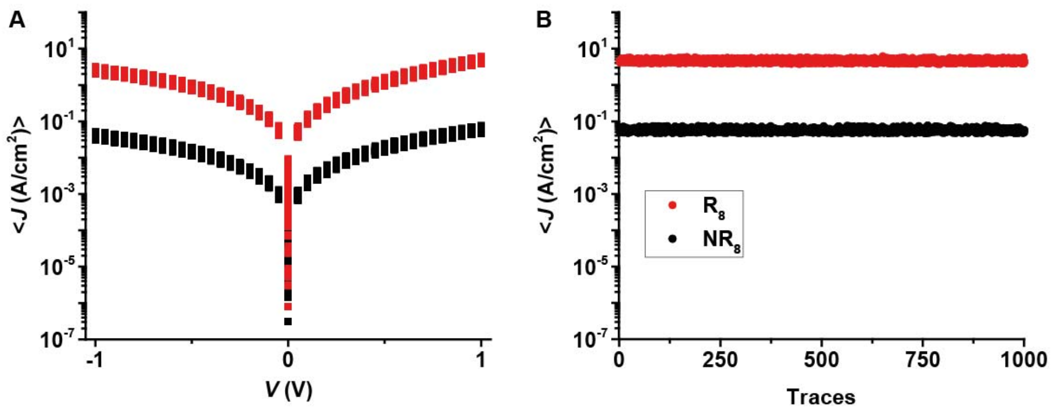

Supplementary Figure 12. A) The 1000 J(V) traces of Au^{TS}-$SC_8PTM^{R/NR}$//GaO_x^{cond}/EGaIn junctions. B) The values of J as a function of trace number with R_8 and NR_8 SAMs at +1.0 V.

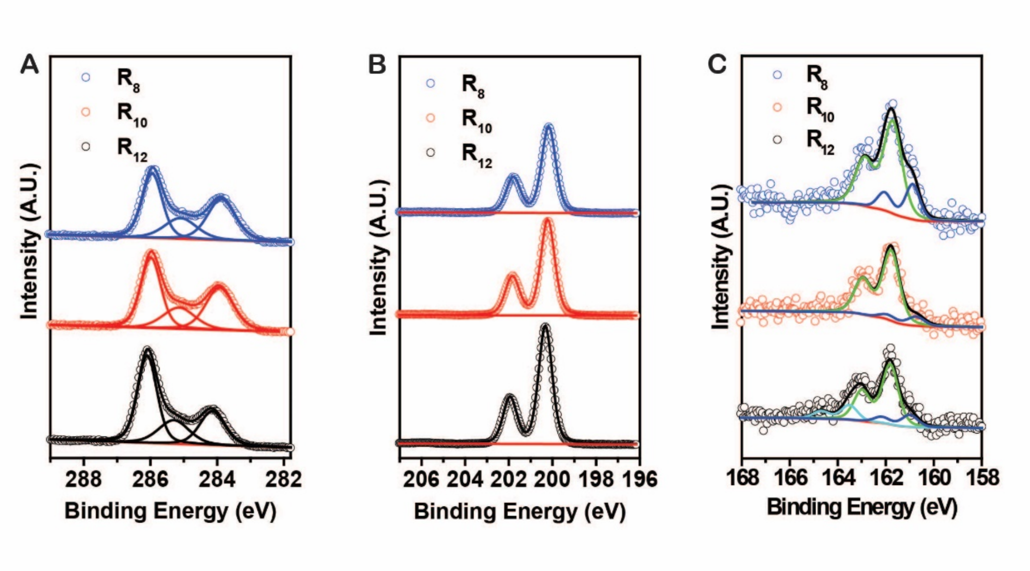

Supplementary Figure 13. The C 1*s* (A), Cl 2*p* (B), and S 2*p* (C) spectra of Au^{TS}-SC_nPTM^R (n= 8, 10, 12; R_8, R_{10} and R_{12} as indicated in the panels) with a take-off angle of 90°.

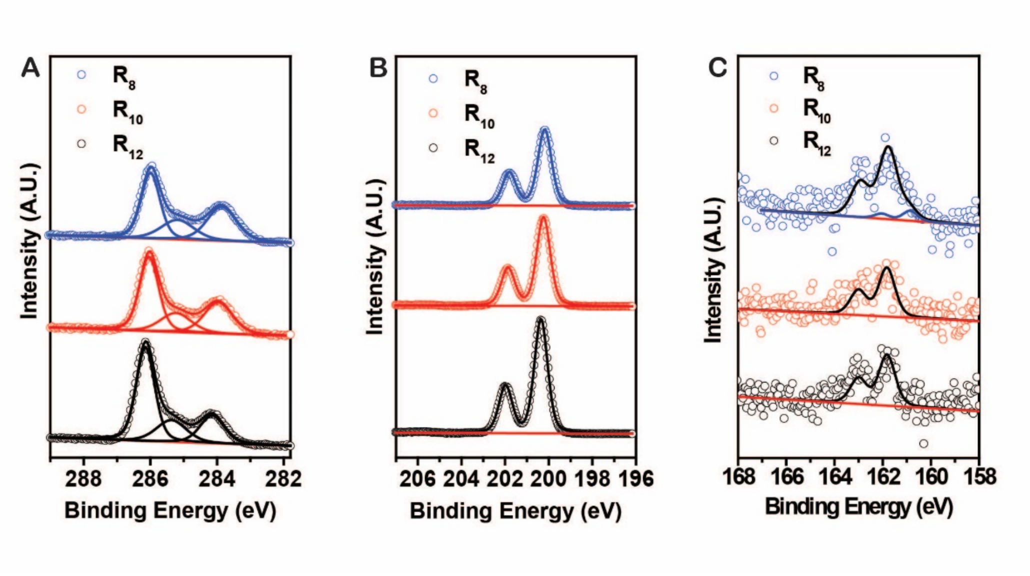

Supplementary Figure 14. The C 1*s* (A), Cl 2*p* (B), and S 2*p* (C) spectra of Au^{TS}-SC_nPTM^R (n= 8, 10, 12; R_8, R_{10} and R_{12} as indicated in the panels) with a take-off angle of 40°.

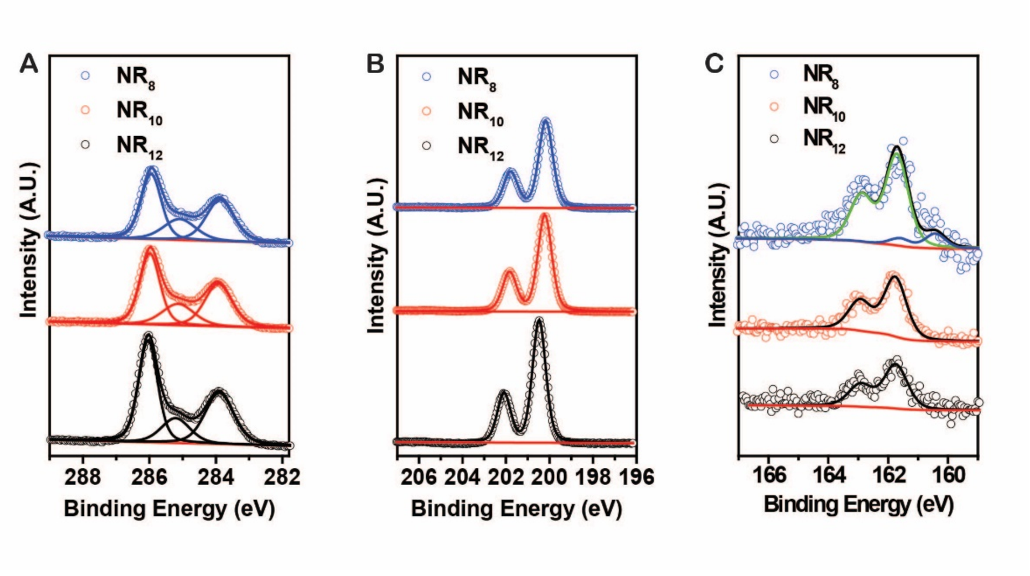

Supplementary Figure 15. The C 1*s* (A), Cl 2*p* (B), and S 2*p* (C) spectra of Au^{TS}-SC_nPTM^{NR} (n= 8, 10, 12; NR_8, NR_{10} and NR_{12} as indicated in the panels) with a take-off angle of 90°.

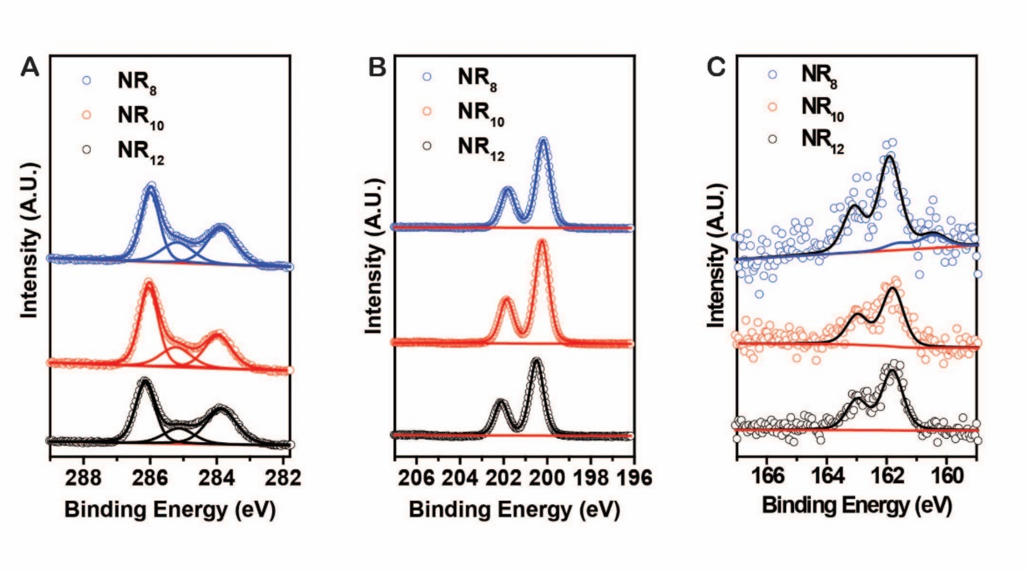

Supplementary Figure 16. The C 1*s* (A), Cl 2*p* (B), and S 2*p* (C) spectra of Au^{TS}-SC_nPTM^{NR} (n= 8, 10, 12; NR_8, NR_{10} and NR_{12} as indicated in the panels) with a take-off angle of 40°.

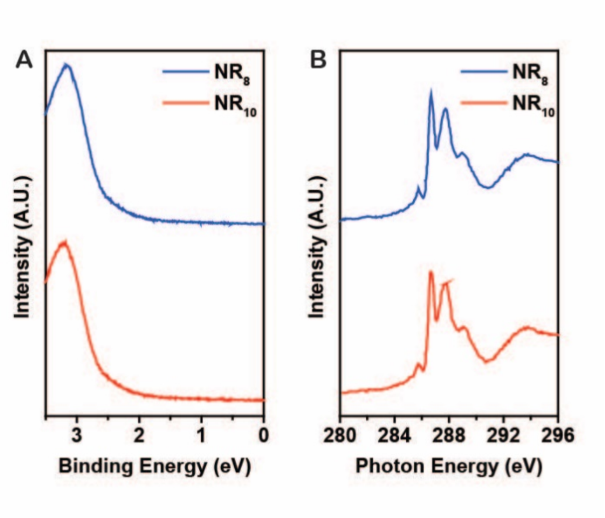

Supplementary Figure 17. UPS and NEXAFS spectra of Au^{TS}-SC_nPTM^{NR} (n= 8 and10; NR_8 and NR_{10} respectively in the figures).

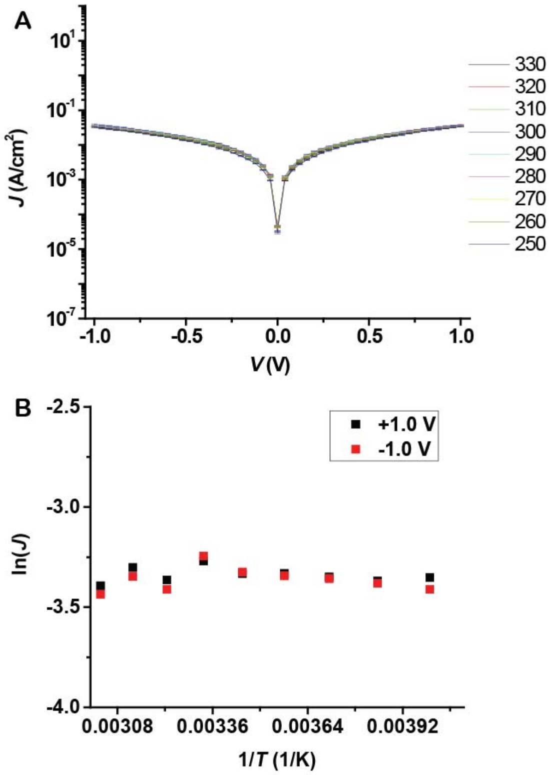

Supplementary Figure 18. Temperature dependent *J*(V) curves and the corresponding Arrhenius plot at *V* = ±1.0 V over the range of temperatures of 250 to 330 K of a junctions with a SAMs of NR_8.

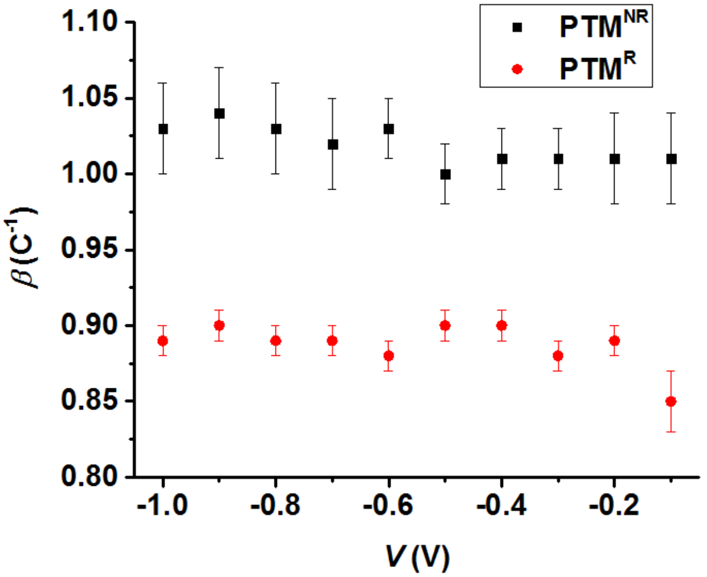

Supplementary Figure 19. The plot of tunneling decay coefficient (β) against V.

Supplementary Table 1. The values of I_{Cl}, relative surface coverage and absolute surface coverage.

SAMs	I_{Cl} at 90° take-off angle	Relative surface coverage[a]	Absolute surface coverage (× 10^{-9} mol/cm^2)
R_8	47997	0.13	0.14
R_{10}	49774	0.13	0.15
R_{12}	52305	0.14	0.16
NR_8	49225	0.13	0.15
NR_{10}	50556	0.14	0.15
NR_{12}	52907	0.14	0.16
$SC_{11}Cl$	26450	1	1.10

[a] The relative surface coverage is calculated from $I_{Cl}(SC_{11}Cl)$ / ($I_{Cl}(PTM^{R/NR})$ / 14).

Supplementary Table 2. The elemental ratios calculated from the high-resolution XPS spectra

SAM	C 1*s* at 90°		C 1*s* at 40°		Cl/C_3	
	C_1/C_3	C_2/C_3	C_1/C_3	C_2/C_3	At 90°	At 40°
R_8	0.87	0.52	0.80	0.52	3.69	4.03
R_{10}	0.73	0.49	0.62	0.47	3.70	4.09
R_{12}	0.54	0.49	0.40	0.44	3.69	4.12
NR_8	0.87	0.51	0.80	0.51	3.68	4.06
NR_{10}	0.72	0.48	0.60	0.47	3.70	4.11
NR_{12}	0.57	0.51	0.39	0.44	3.68	4.20

Supplementary Table 3. The ratio of (I_θ) of S 2p between take-off angles of 90º and 40º of R-based SAMs on Au^{TS}.

	I_θ(90º) (%)	I_θ(40º) (%)	d (nm)
Au^{TS}-SC_8PTM^R	69.6	30.4	1.83
Au^{TS}-$SC_{10}PTM^R$	73.2	26.8	2.05
Au^{TS}-$SC_{12}PTM^R$	75.1	24.9	2.20
Au^{TS}-SC_8PTM^{NR}	68.9	31.1	1.79
Au^{TS}-$SC_{10}PTM^{NR}$	73.5	26.5	2.08
Au^{TS}-$SC_{12}PTM^{NR}$	75.2	24.8	2.22

Supplementary Methods

Synthesis and characterization of PTM derivatives

General procedure

Elemental analyses were performed on the CID (CSIC) services. NMR spectra were recorded on a Bruker Avance 400 MHz. EPR spectra were recorded in a Bruker ELEXYS E500 X-band spectrometer. The simulation of the EPR spectra was realised with software Simfonia. Electrochemical experiments were performed with a potensiostat/galvanostat Autolab/PGSTAT204 from Metrohm Autolab B.V. in a standard three-electrode cell, by using a platinum wire as working and counter electrode and Ag/AgCl as reference electrode. Tetrabutylammonium hexafluorophospate (Fluka, 99%) was used as the supporting electrolyte. UV-Vis spectra were recorded on a Varian Carey 5000 in double-beam mode. Mass Spectra were recorded with a Bruker Ultraflex LDI-TOF mass spectrometer. The liquid chromatography were performed in an Agilent 1100 series HPLC (Agilent Technologies) connected to with two (in parallel) detectord, a photodiode array working into 200nm and 800nm and a mass spectrometer (Esquire 3000 MS Trap (Bruker Daltonik) equipped with Electrospray source ionisation), the methodology used was a binary gradient of acetonitrile (with 5% of formic acid) and chloroform, from 90/10 to 60/40. The IR spectra were recorded with an ATR-IR Perkin Elmer Spectrum One. The manipulation of the radicals in solution was performed under red light.

General procedure for the synthesis of compounds tritylthio-alcohols 1, 2 and 3.

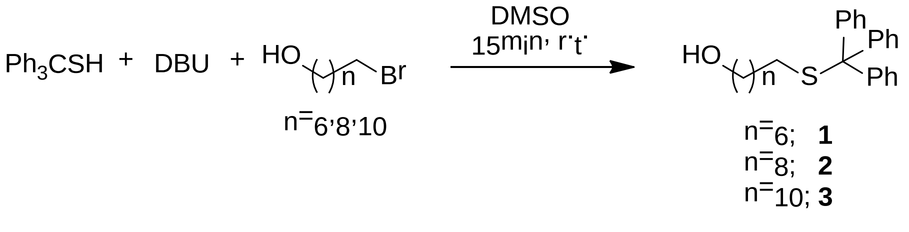

Triphenylmethane thiol (1Eq) was suspended in DMSO and DBU (1.14 Eq,) was added. After stirring at room temperature for 5 min, the corresponding bromo-alchol derivative (1.08 Eq, 0.96 mmol) was added, and the mixture was stirred for 10 min. The reaction mixture was diluted with ethyl acetate, quenched with 0.1M HCl (5 ml), extracted with ethyl acetate, dried over $MgSO_4$ and dried in vacum. The crude product was purified by flash chromatography (SiO_2, heptane/AcOEt 1/1).

General procedure for the synthesis of compounds tritylthio- aldehydes 4, 5 and 6.

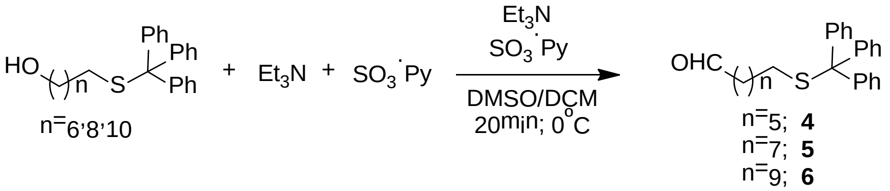

The corresponding Triphenylmercapto-n-ol (1 Eq,) and triethylamine (3.5 Eq,) were dissolved in dichloromethane and DMSO, cooled to 0°C and added a suspension of sulfur trioxide pyridine complex (2.07 Eq, 408 mg, 2.57 mmol) in DMSO (400 μL). At the same temperature, the mixture was then stirred for 20 min at 0ºC and after diluted with dichloromethane and quenched with 0.1M HCl (5ml) and extracted with dichloromethane. The combined organic phases were washed with brine and dried ($MgSO_4$) The crude product was purified by flash chromatography (heptane/AcOEt 1/1).

General procedure for the synthesis of PTM derivatives 7, 8 and 9.

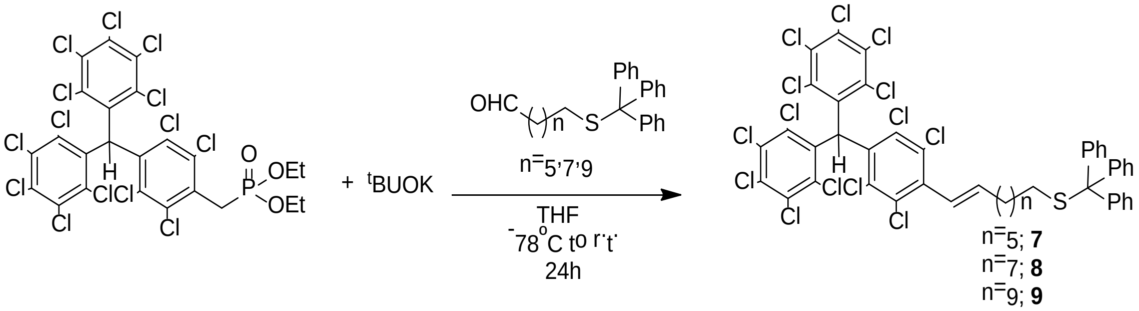

Under Argon, potassium tert-butoxide (1.5 Eq) was added to a stirred solution of PTM-$P(O)(OEt)_2$ (1 Eq) in dry tetrahydrofuran (THF) at -78ºC and the mixture was stirred at this temperature for 10min, then a solution of the corresponding triphenylmercapto-n-al (2 Eq) in dry THF was added and the mixture was allowed to warm to room temperature. The resulting mixture was stirred in the dark at room temperature for 24h. After evaporation of the solvent the crude was purified by column chromatography (SiO_2, Hexane/CH_2Cl_2 80/20).

General procedure for the synthesis of PTM radical derivatives 10, 11 and 12.

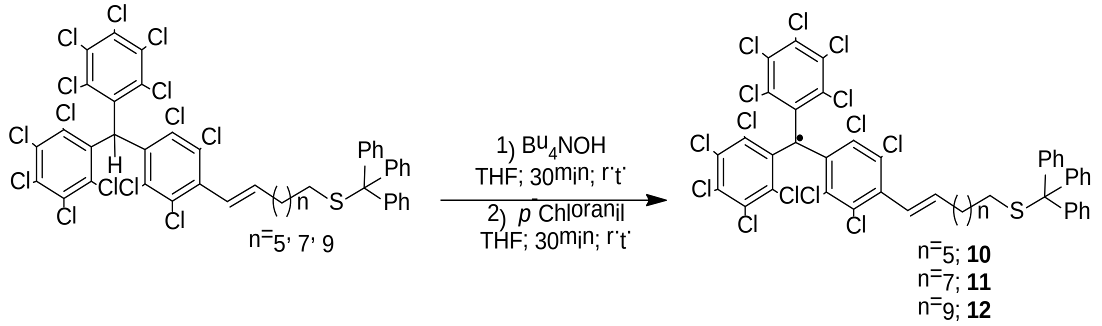

To a solution of the corresponding αH PTM triphenylmercapto derivative in THF, 1.3 Eq of Bu_4NOH (1M in methanol) was added. The initial colorless solution turned to intense violet and the reaction mixture was stirred at room temperature for 30 min. and then, 1.5 Eq of *p*-chloranil was added. The color of the reaction mixture changed to red. After 30 min the solvent was removed under vacuum and the crude product was purified by flash chromatography (SiO_2, Hexane/$CH_2Cl_2$80/20). The resulting waxy compounds were washed with methanol to give a red solid in almost quantitative yield (96-98%).

General procedure to generation of the free thiol groups

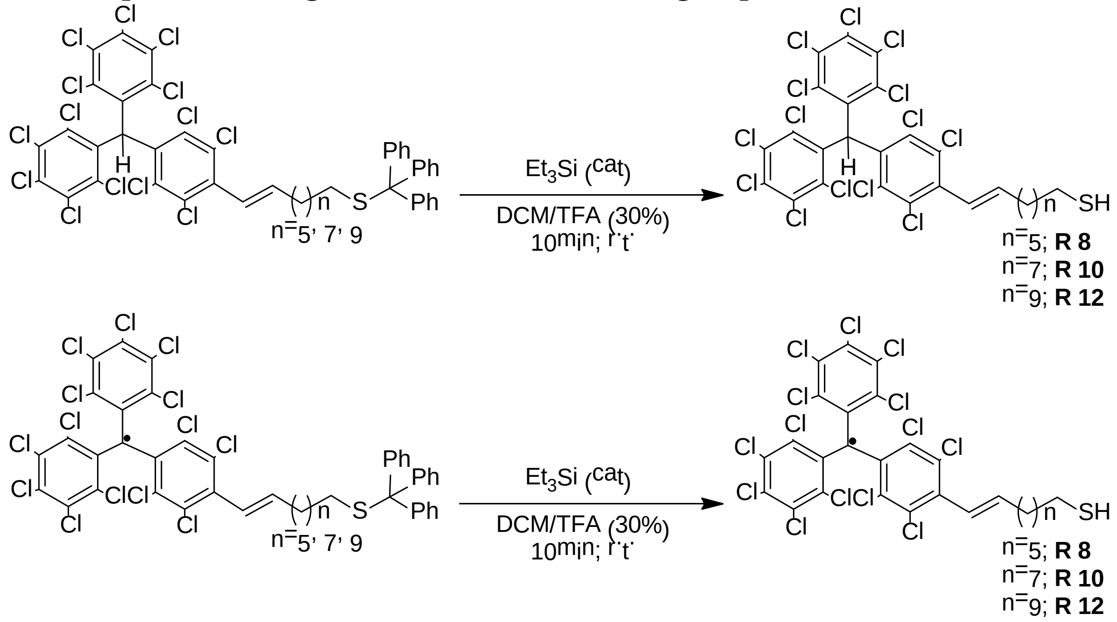

To a solution of trifluoroacetic acid (TFA) in DCM (30%) triethylsilyl (cat) was added under argon, and then the PTM triphenylmercapto derivative (either radical or αH) was added to the mixture. The solution was stirred at room temperature in the dark for 10 min. Then the solvent was evaporated in vacuum, and the crude product was purified by flash chromatography (silica gel, Hexane/CH_2Cl_2 80/20). The product was washed with methanol in a sonicated bath and filtered several time, to yield a solid (red for radicals and withe for αHs) in almost quantitative yield (95%-97%)

7-(tritylthio)heptan-1-ol (1)

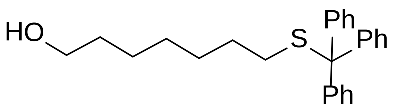

According to the general procedure, from triphenylmethane thiol (247 mg, 0.89 mmol), DBU (1.14 Eq, 152 μL, 1.02 mmol) and 7bromo-heptanol (187 mg, 0.96 mmol) in DMSO (1 mL), the compound **1** was obtained as a transparent oil (yield, 84%). **^{1}H-NMR** (400 MHz, CD_2Cl_2) *δ*/ppm: 7.49 (m, 6H), 7.35 (m, 6H), 7.28(m, 3H), 3.61 (t, *J* = 6.6 Hz, 2H), 2.21 (t, *J* = 7.4 Hz, 2H), 1.64 – 1.14 (m, 11H); **^{13}C- NMR** (101 MHz, CD_2Cl_2) δ/ppm: 145.17, 129.60, 127.79, 126.51, 70.74, 62.69, 32.75, 31.89, 28.97, 28.95, 28.54, 25.55. **FT-IR** *ν*/cm^{-1}: 3345.9, 3085.0, 3055.8, 3024.1, 2927.5, 2853.9, 1594.7, 1488.5, 1443.4, 1183.3, 1079.6, 1054.8, 1033.9, 1001.6, 884.2, 850.1, 764.7, 741.3, 697.8, 676.3, 617.1; **EM (m/z)** (ESI): calculated for $C_{26}H_{30}OS$: 390.2; found: 413.2 (M+Na), 276.2(·SCPh3+1H$^+$), 243.11 (·CPh3+1H$^+$).

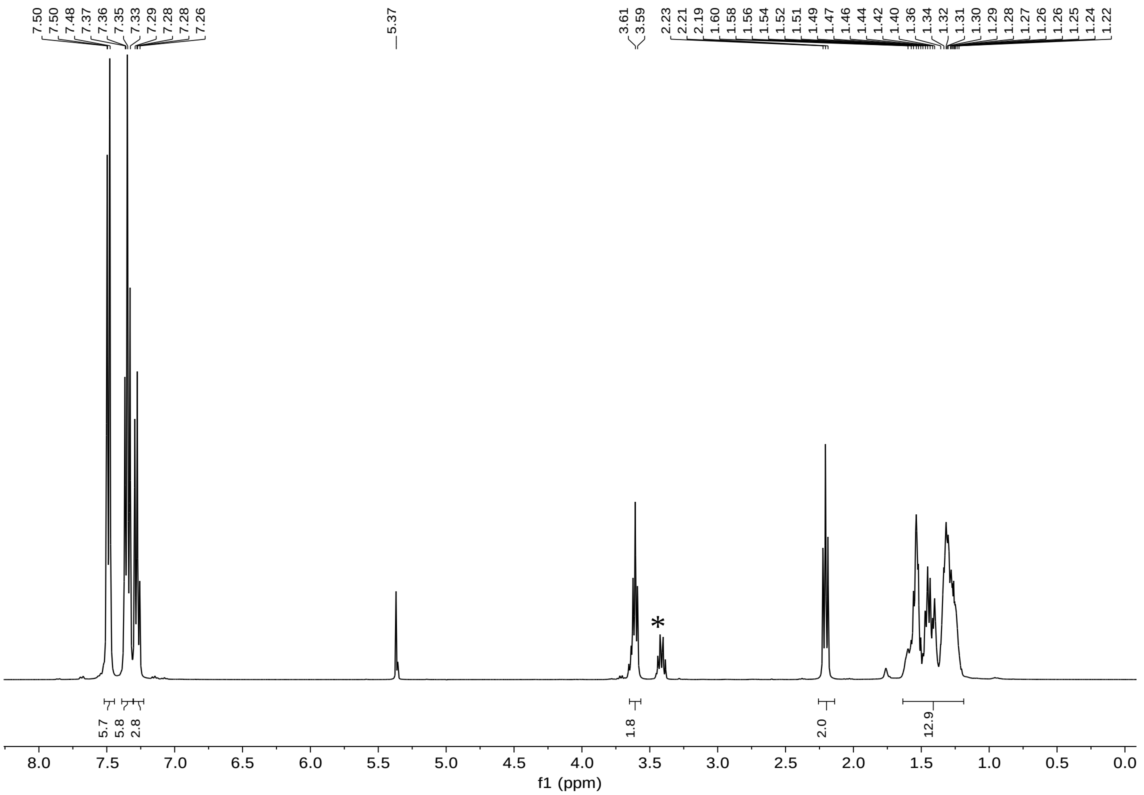

** Impurity that does not affect to the rest of the synthesis. Probably a fragment of the alkyl chain.*

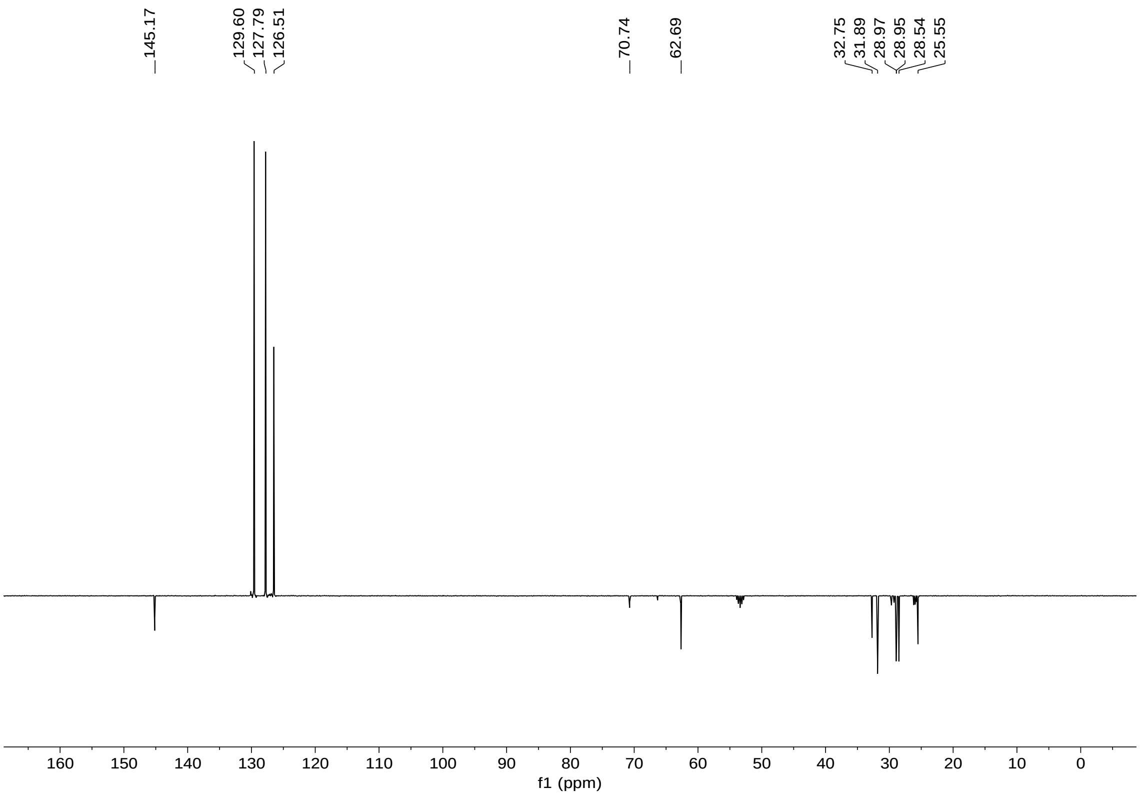

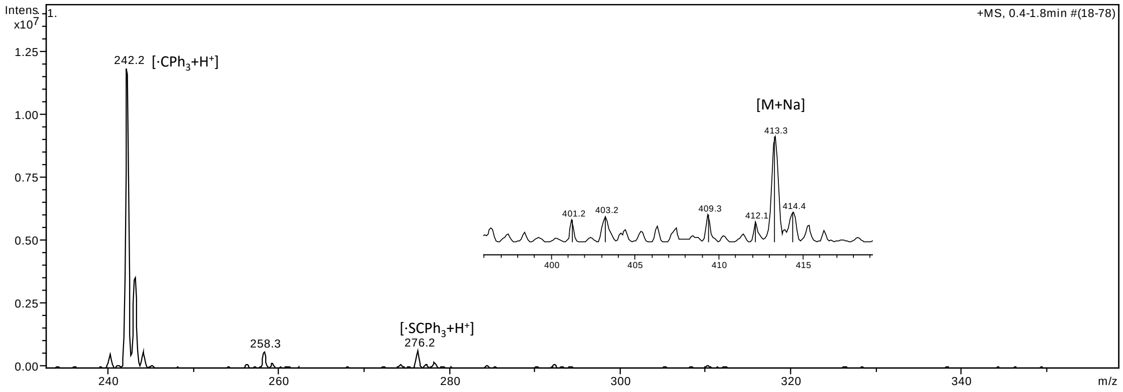

9-(tritylthio)nonan-1-ol (2)

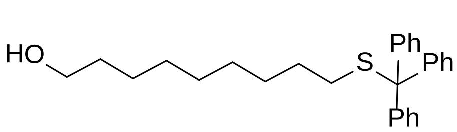

According to the general procedure, from triphenylmethane thiol (247 mg, 0.89 mmol), DBU (152 µL, 1.02 mmol) and 9-bromo-nonanol (214 mg, 0.96 mmol) in DMSO (1 mL) the compound **2** was obtained as a transparent oil (yield, 85%), **^{1}H-NMR** (400 MHz, $CDCl_3$) δ/ppm: 7.49 – 7.39 (m, 6H), 7.34 – 7.25 (m, 6H), 7.22 (t, *J* = 7.2 Hz, 3H), 3.63 (t, *J* = 6.6 Hz, 2H), 2.16 (t, *J* = 7.3 Hz, 2H), 1.56 (p, *J* = 6.6 Hz, 2H), 1.45 (s, 1H, OH), 1.40 (q, *J* = 7.5 Hz, 2H), 1.37 – 1.11 (m, 10H). **^{13}C-NMR** (101 MHz, $CDCl_3$) δ/ppm: 145.11, 129.64, 127.82, 126.53, 77.42, 77.10, 76.78, 66.40, 63.02, 32.79, 32.05, 29.38, 29.34, 29.13, 29.01, 28.62, 25.73; **FT-IR** ν/cm^{-1}: 338.0, 3083.3, 3057.8, 3026.7, 2927.7, 2851.3, 1595.0, 1488.6, 1443.1, 1179.7, 1076.3, 1054.1,

1034.1, 1001.9, 885.2, 850.8, 765.2, 740.8, 697.5, 677.4. **EM (m/z)** (MALDI-TOF): calculated for $C_{28}H_{34}OS$: 418.23; found: 417.23 (M-H), 275.08($\cdot SCPh_3$), 243.11 ($\cdot CPh_3$) and 175.13 (M-CPh_3).

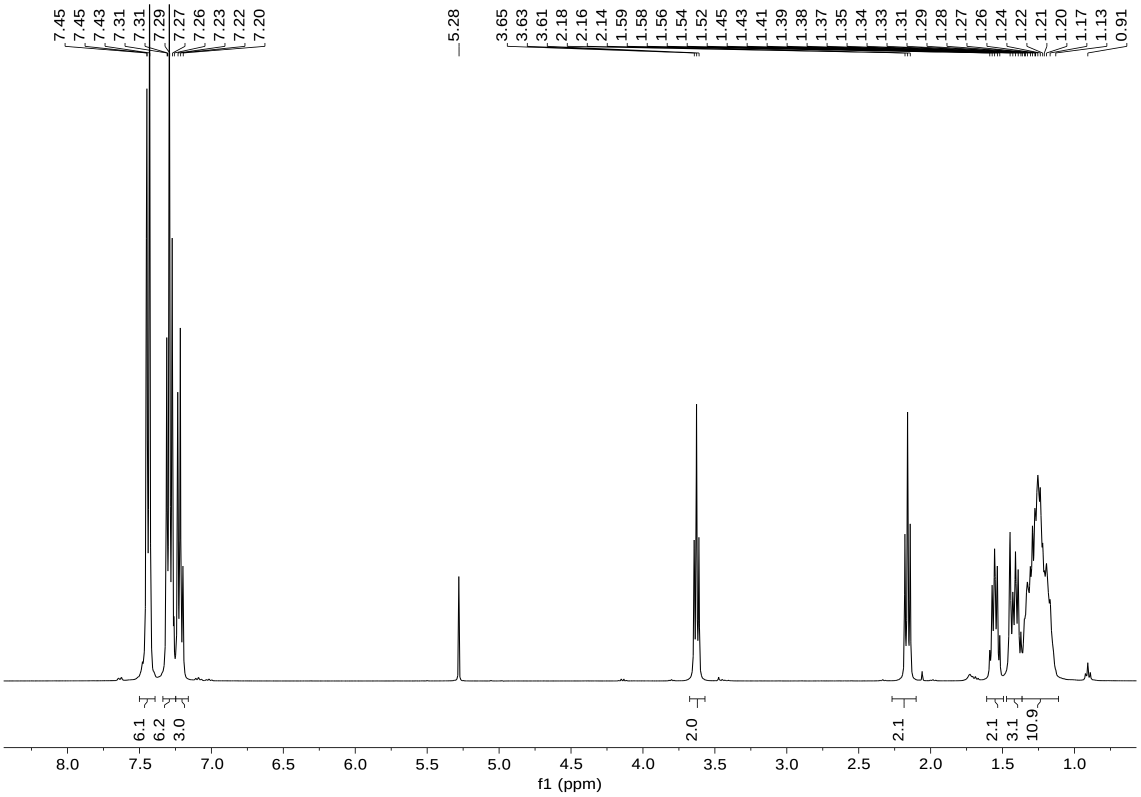

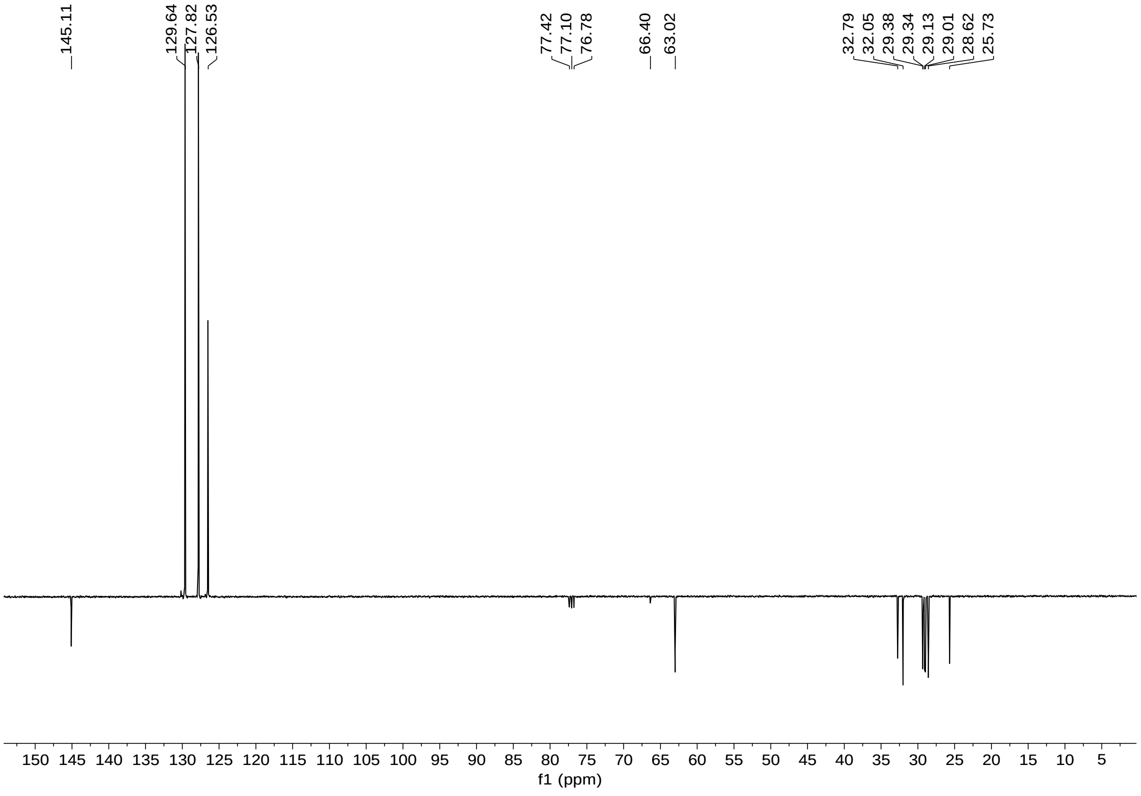

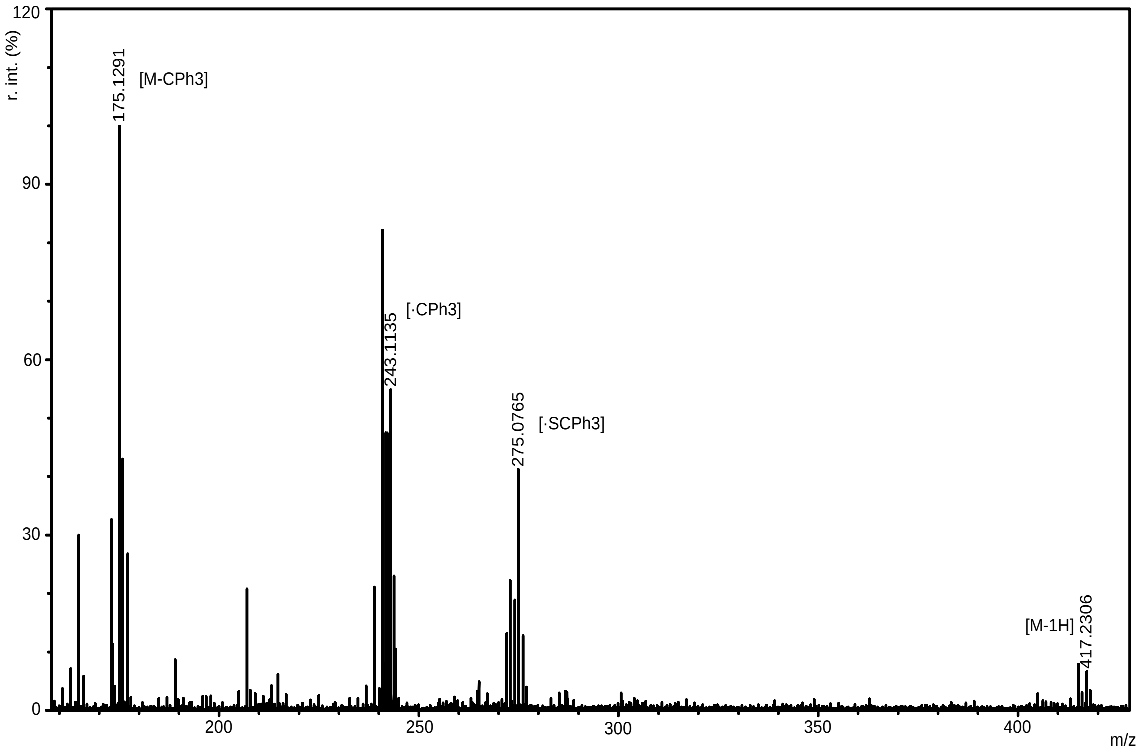

11-(tritylthio)undecan-1-ol (3)

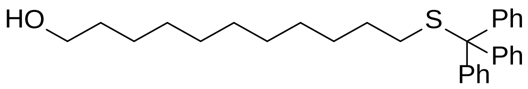

According to general procedure, from triphenylmethane thiol (247 mg, 0.89 mmol), DBU (152 μL, 1.02 mmol) and 11-bromo-undecanol (241 mg, 0.96 mmol) in DMSO (1 mL), the compound **3** was obtained as a transparent oil (yield, 79%). **^{1}H-NMR** (400 MHz, CD_2Cl_2) *δ*/ppm: 7.54 – 7.47 (m, 6H), 7.36 (t, *J* = 7.5 Hz, 6H), 7.29 (t, J = 7.2 Hz, 3H), 3.65 (t, *J* = 6.6 Hz, 2H), 2.22 (t, *J* = 7.4 Hz, 2H), 1.71 (s, 1H), 1.61 (q, *J* = 6.7 Hz, 2H), 1.53 – 1.16 (m, 16H); **^{13}C-NMR** (101 MHz, CD_2Cl2) δ/ppm: 145.20, 129.62, 127.81, 126.53, 66.35, 62.79, 53.46, 32.91, 31.95, 29.63, 29.54, 29.50, 29.45, 29.20, 29.06, 28.63, 25.83. **FT-IR** *ν*/cm^{-1}: 3327.5, 3085.2, 3058.0, 3023.0, 2923.9, 2852.6, 1595.1, 1488.9, 1443.8 1183.5, 1077.4, 1055.3, 1033.9, 1001.7, 884.7, 852.1, 741.4, 967.8, 676.2, 617.3; **EM (m/z)** (MALDI-TOF): calculated for $C_{30}H_{38}OS$: 446.22; found: 445.29 (M-1H) and 243.13 (·CPh_3).

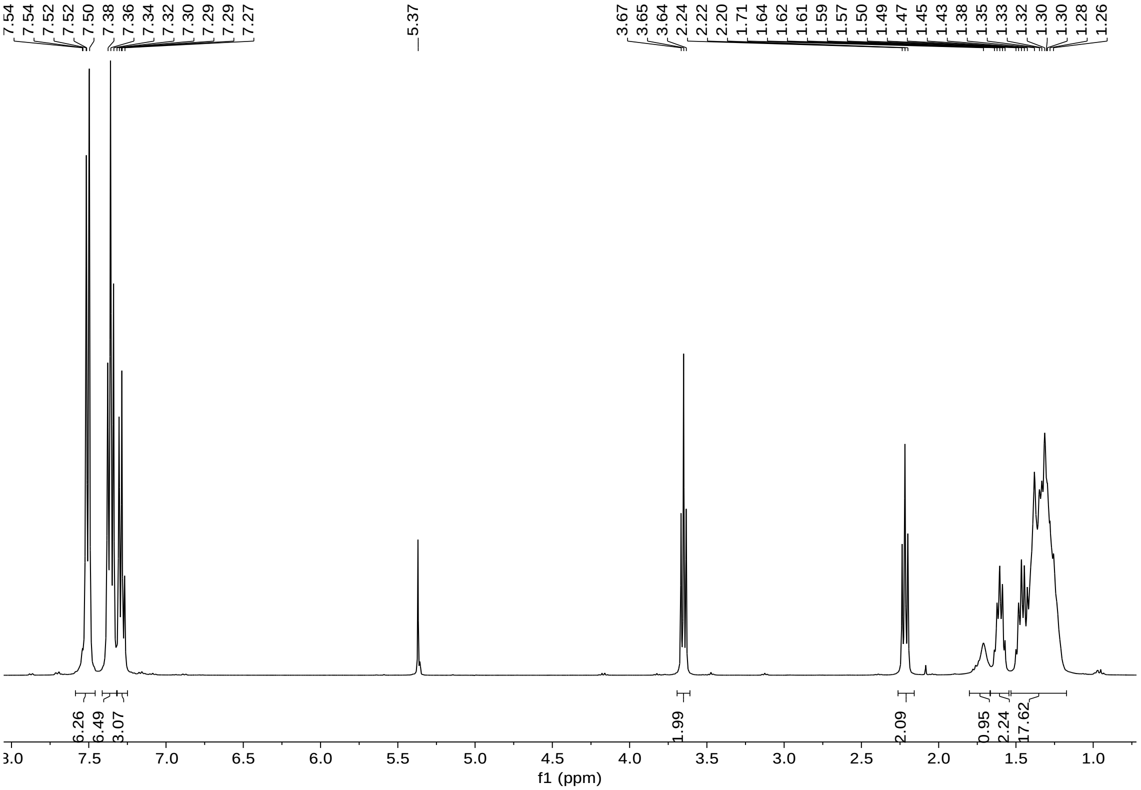
7.54
7.54
7.52
7.52
7.50
7.38
7.36
7.34
7.32
7.30
7.29
7.29
7.27
5.37
3.67
3.65
3.64
2.24
2.22
2.20
1.71
1.64
1.62
1.61
1.59
1.57
1.50
1.49
1.47
1.45
1.43
1.38
1.35
1.33
1.32
1.30
1.30
1.28
1.26
6.26
6.49
3.07
1.99
2.09
0.95
2.24
17.62
f1 (ppm)

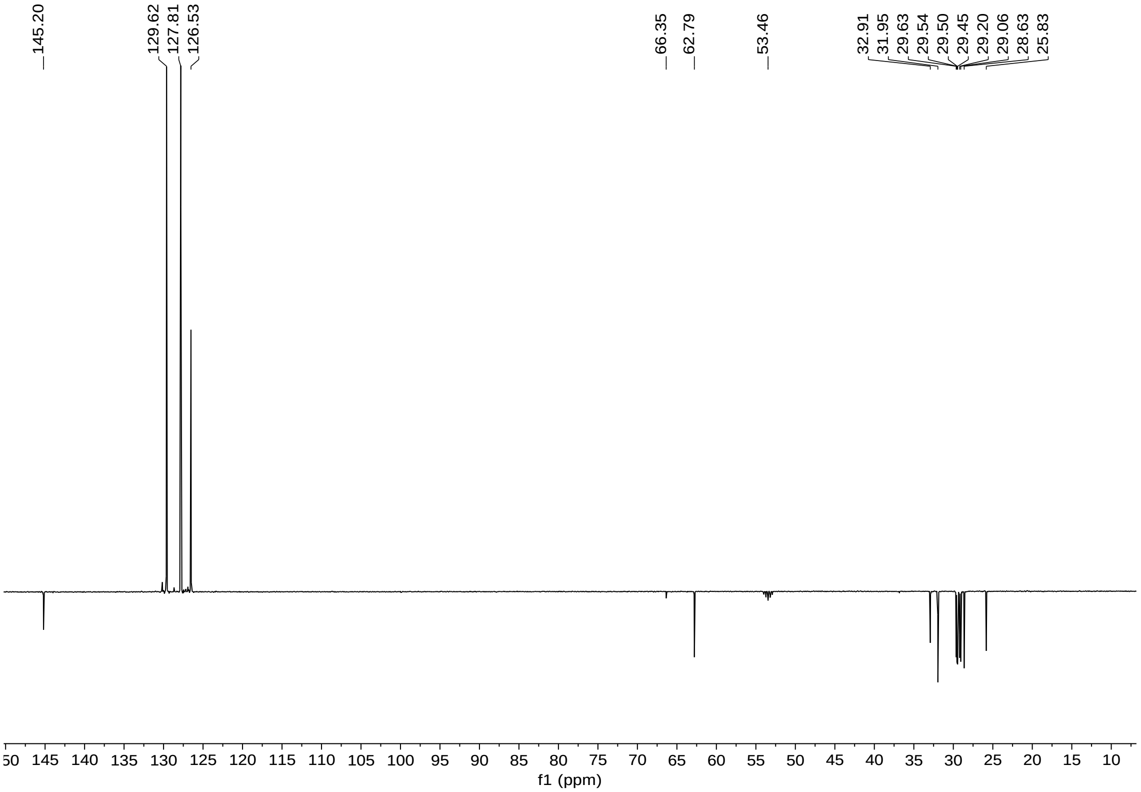
145.20
129.62
127.81
126.53
66.35
62.79
53.46
32.91
31.95
29.63
29.54
29.50
29.45
29.20
29.06
28.63
25.83
f1 (ppm)

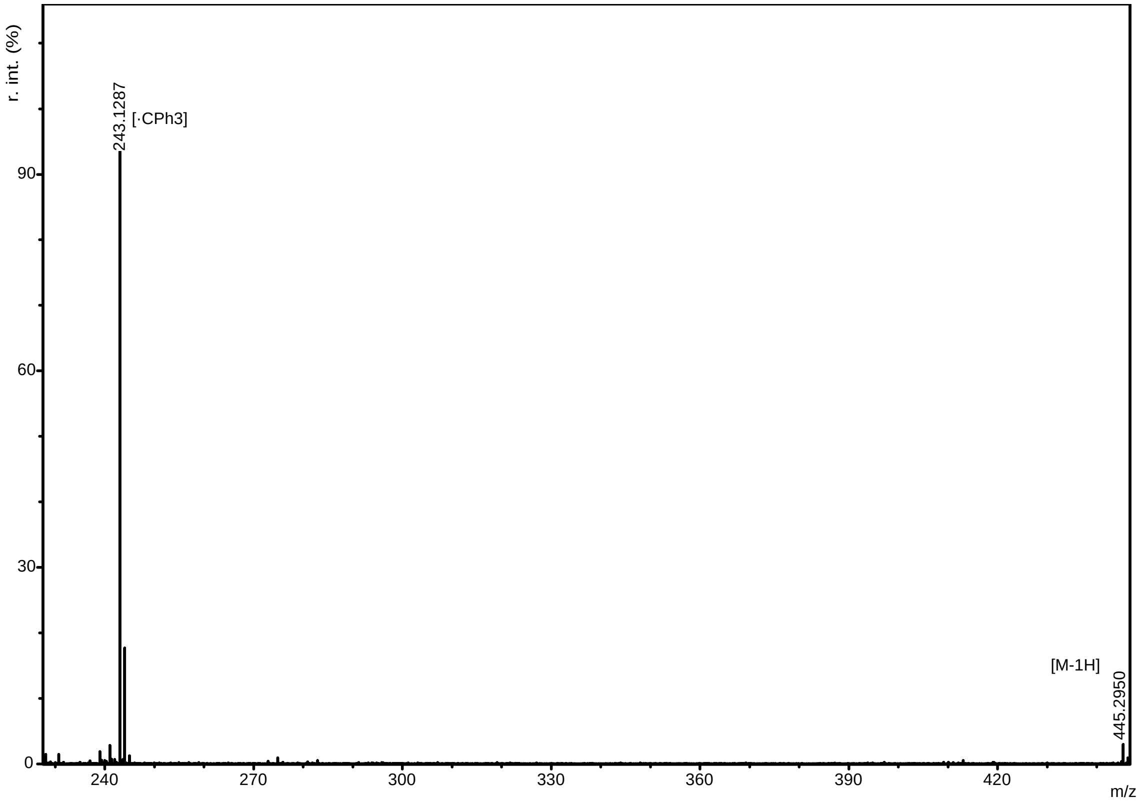

7-(tritylthio)heptanal (4)

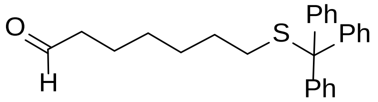

According to general procedure, from 7-triphenylmercapto-1-ol (**1**) (484 mg, 1.24 mmol) triethylamine (600 µL, 4.34 mmol) and sulfur trioxide pyridine complex (408 mg, 2.57 mmol) in mixture of DCM (3.5 mL) and DMSO (500 µL), the compound **4** was obtained as a transparent oil (yield, 66%). **^{1}H-NMR** (400 MHz, CD_2Cl_2) δ/ppm: 9.75 (t, J = 1.7 Hz, 1H), 7.56 – 7.43 (m, 6H), 7.35 (t, J = 7.5 Hz, 7H), 7.28 (t, J = 7.2 Hz, 3H), 2.40 (td, J = 7.4, 1.7 Hz, 2H), 2.21 (t, J = 7.3 Hz, 2H), 1.59 (p, J = 7.4 Hz, 2H), 1.51 – 1.38 (m, 2H), 1.38 – 1.19 (m, 4H). **^{13}C-NMR** (101 MHz, CD_2Cl_2) δ/ppm: 202.40, 145.14, 129.59, 127.80, 126.53, 70.75, 70.62, 43.71, 31.79, 28.68, 28.64, 28.40, 21.84; **FT-IR** ν/cm^{-1}: 3083.7, 3056.6, 3028.8, 2929.2, 2855.5, 2721.2, 1721.9, 1593.5, 1485.6, 1440.8, 1238.1, 1079.6, 1033.9, 742.2, 698.3, 674.5, 625.5. **EM (m/z)** (ESI): calculated for $C_{26}H_{28}OS$: 388.2; found: 411.2 (M+Na), 243.11 (·CPh3+1H+).

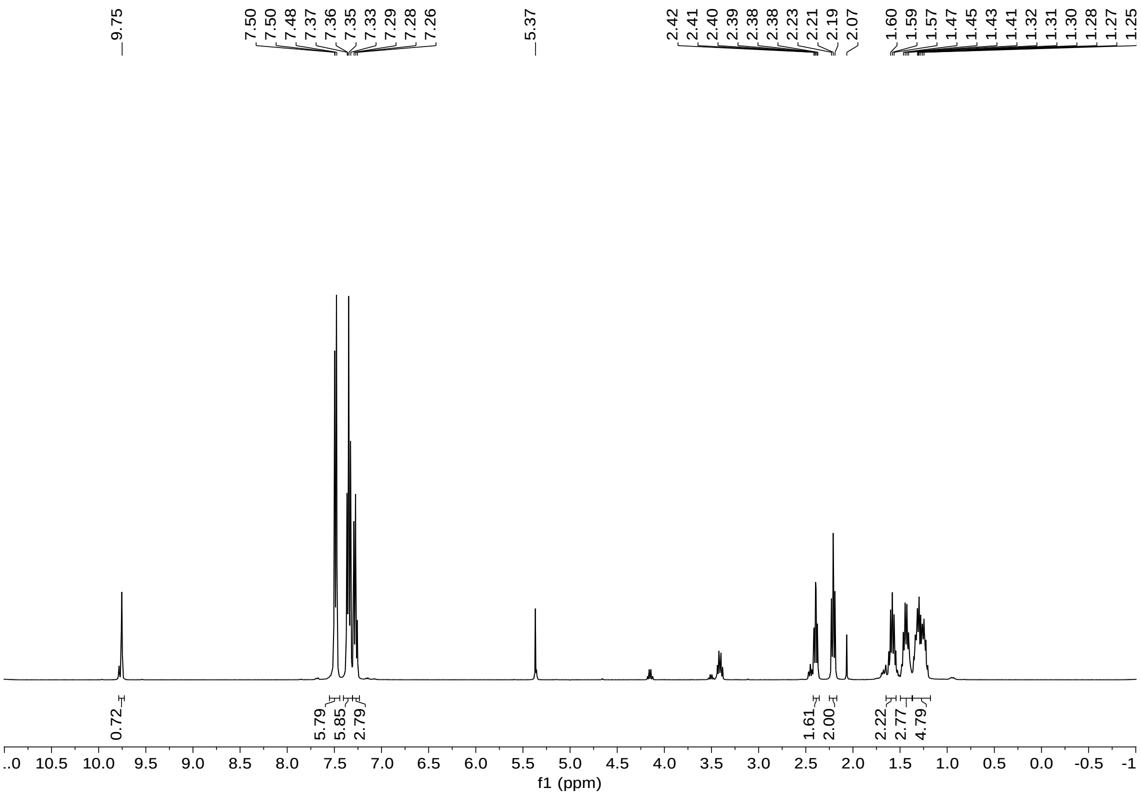
9.75
7.50
7.50
7.48
7.37
7.36
7.35
7.33
7.29
7.28
7.26
5.37
2.42
2.41
2.40
2.39
2.38
2.38
2.23
2.21
2.19
2.07
1.60
1.59
1.57
1.47
1.45
1.43
1.41
1.32
1.31
1.30
1.28
1.27
1.25
0.72
5.79
5.85
2.79
1.61
2.00
2.22
2.77
4.79
f1 (ppm)

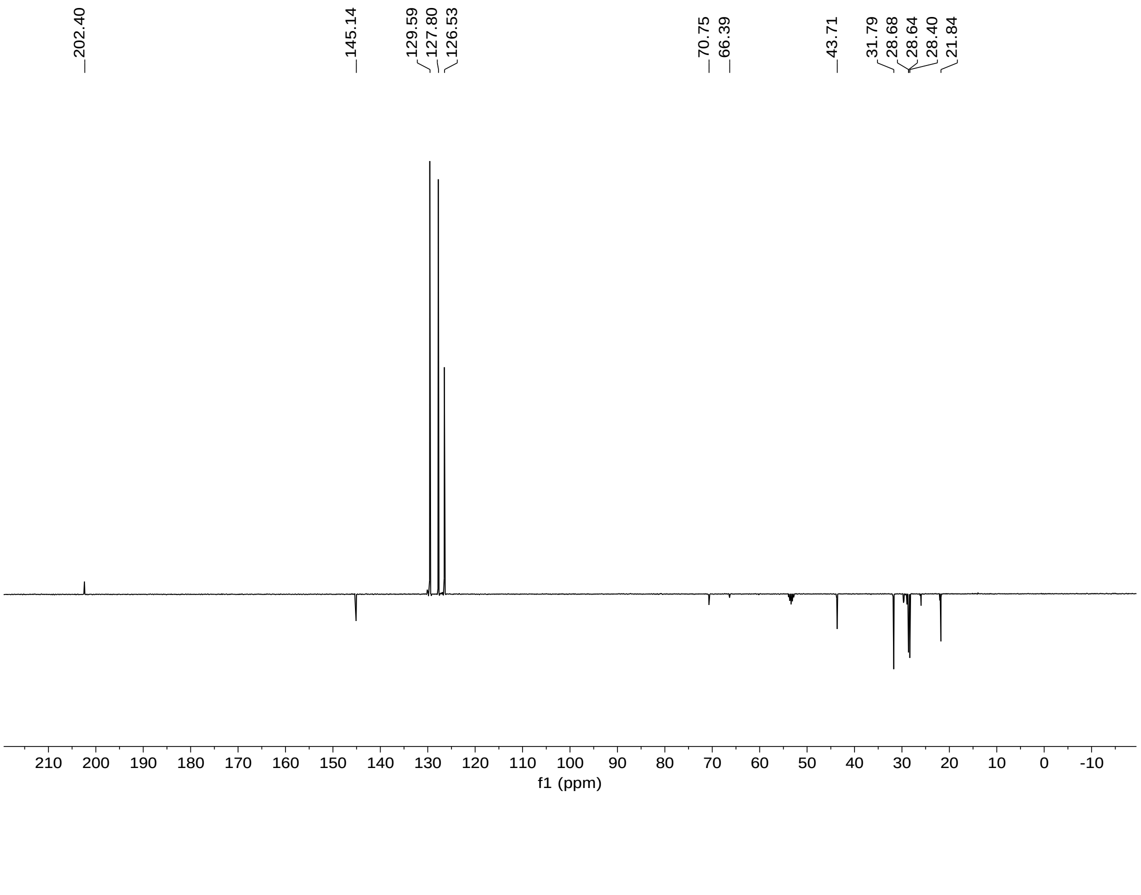
202.40
145.14
129.59
127.80
126.53
70.75
66.39
43.71
31.79
28.68
28.64
28.40
21.84
f1 (ppm)

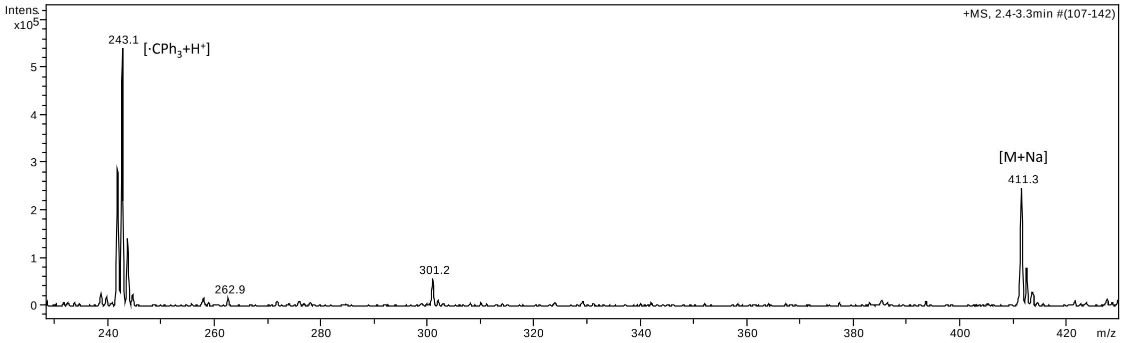

9-(tritylthio)nonanal (5)

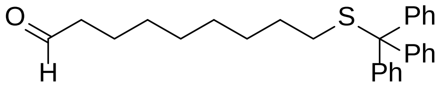

According to the general procedure, from 9-triphenylmercapto-1-ol (**2**) (1.24 mmol, 519 mg), triethylamine (600 µL, 4.34 mmol) and sulfur trioxide pyridine complex (408 mg, 2.57 mmol) in a mixture of DCM (3.5 mL) and DMSO (500 µL), the compound **5** was obtained as a transparent oil (yield, 71%). **^{1}H-NMR** (400 MHz, $CDCl_3$) δ/ppm: 9.84 (t, J = 1.6 Hz, 1H), 7.67 – 7.57 (m, 6H), 7.42 (t, J = 7.5 Hz, 6H), 7.35 (t, J = 7.2 Hz, 3H), 2.49 (td, J = 7.4, 1.6 Hz, 2H), 2.34 (t, J = 7.3 Hz, 2H), 1.72 (q, J = 7.2 Hz, 2H), 1.56 (q, J = 7.1 Hz, 2H), 1.50 – 1.26 (m, 8H); **^{13}C-NMR** (101 MHz, CD_2Cl2) δ/ppm: 202.42, 145.31, 129.74, 127.95, 126.66, 66.52, 43.94, 32.04, 29.29, 29.19, 29.09, 29.06, 28.72, 22.16; **FT-IR** ν/cm^{-1}: 3086.5, 3057.1, 3032.2, 2922.8, 2852.5, 1722.9, 1594.7, 1488.7, 1444.3, 155.5, 10841.1, 1034.1, 952.9, 887.2, 850.2, 164.3, 742.4, 697.8, 676.7; **EM (m/z)** (MALDI-TOF): calculated for $C_{28}H_{32}OS$: 416.22; found: 415.21 (M-H) and 243.08 ($M-CPh_3$).

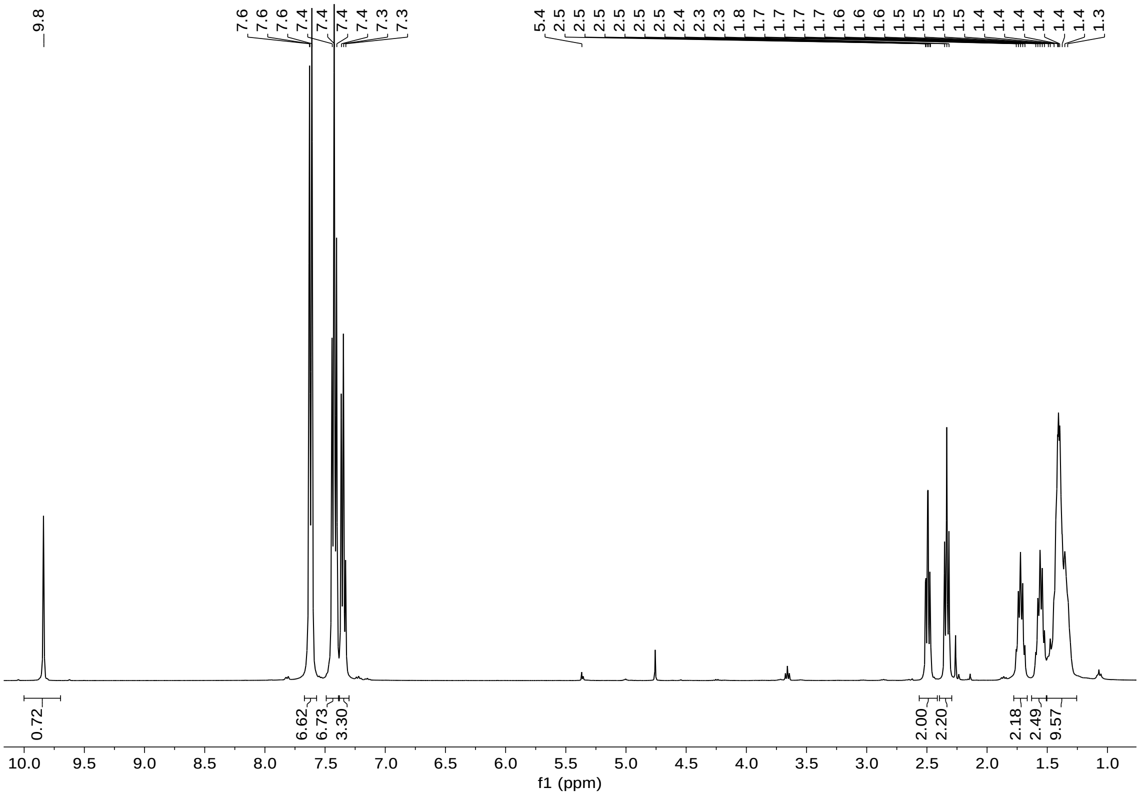
9.8
7.6 7.6 7.6 7.4 7.4 7.4 7.4 7.3 7.3
5.4 2.5 2.5 2.5 2.5 2.5 2.5 2.4 2.3 2.3 1.8 1.7 1.7 1.7 1.6 1.6 1.6 1.5 1.5 1.5 1.5 1.4 1.4 1.4 1.4 1.4 1.4 1.3
0.72
6.62 6.73 3.30
2.00 2.20
2.18 2.49 9.57
10.0 9.5 9.0 8.5 8.0 7.5 7.0 6.5 6.0 5.5 5.0 4.5 4.0 3.5 3.0 2.5 2.0 1.5 1.0
f1 (ppm)

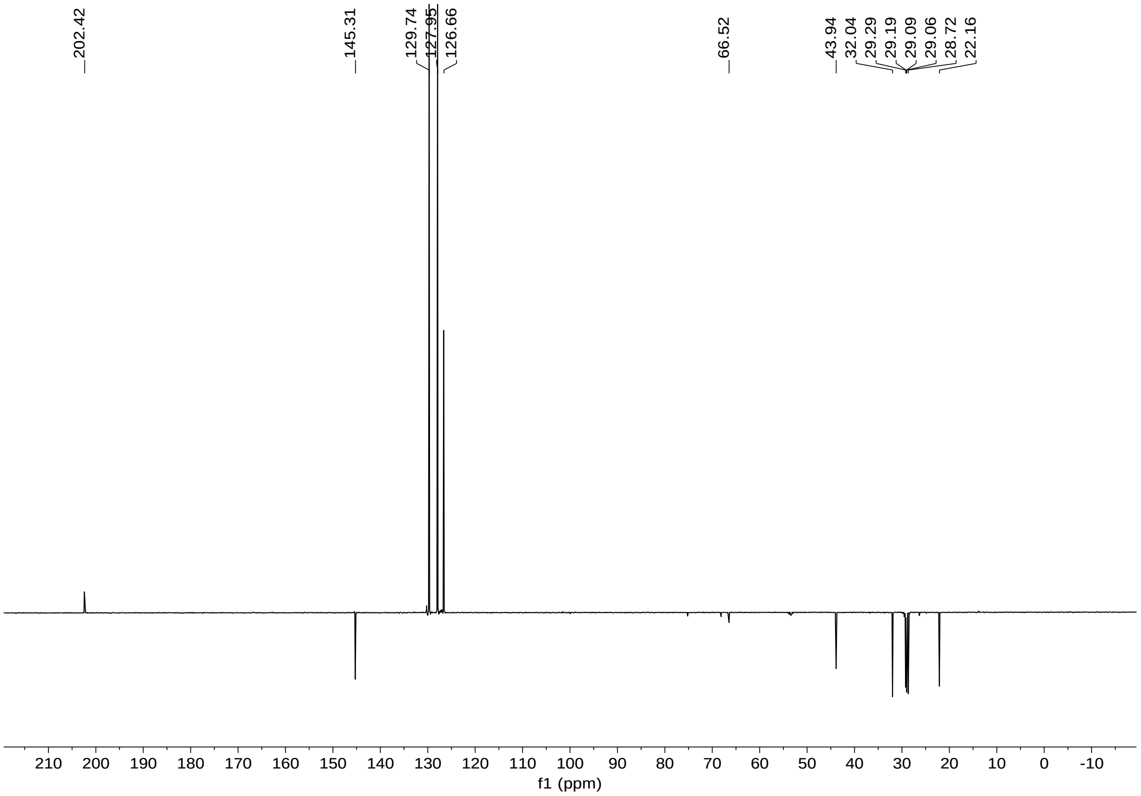
202.42
145.31
129.74 127.95 126.66
66.52
43.94 32.04 29.29 29.19 29.09 29.06 28.72 22.16
210 200 190 180 170 160 150 140 130 120 110 100 90 80 70 60 50 40 30 20 10 0 -10
f1 (ppm)

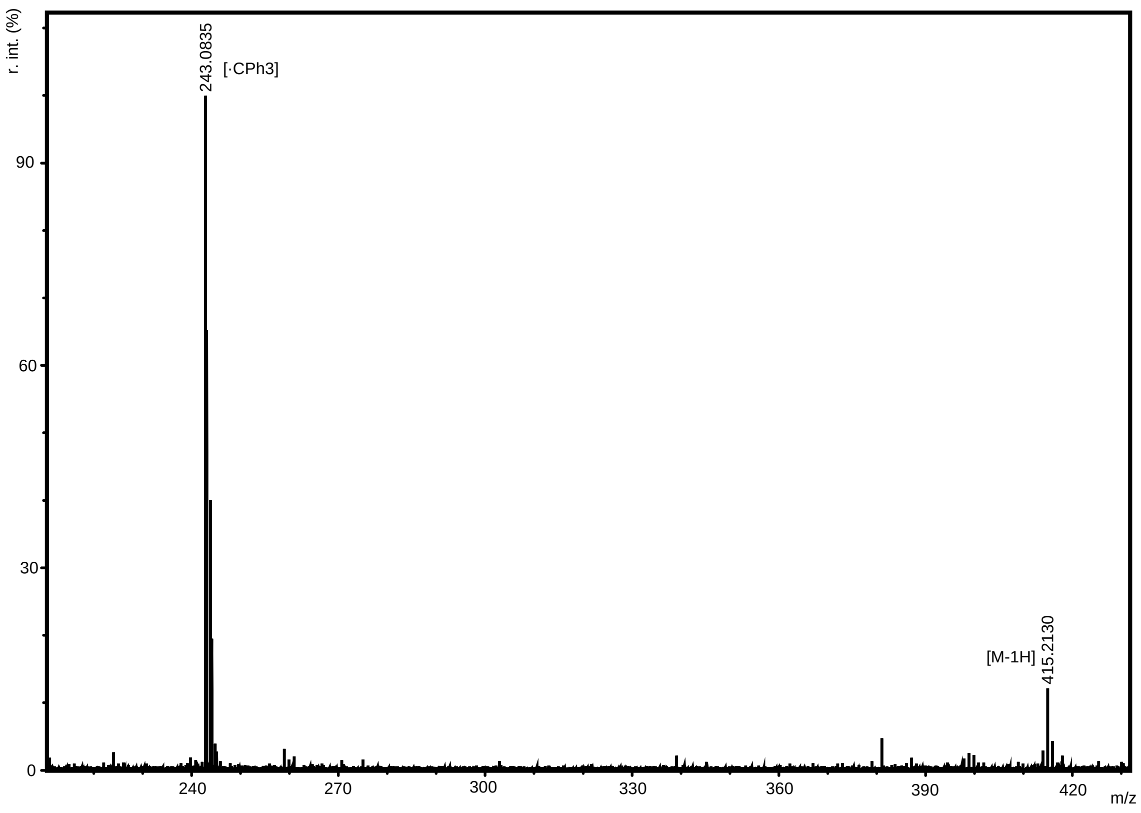

11-(tritylthio)undecanal (6)

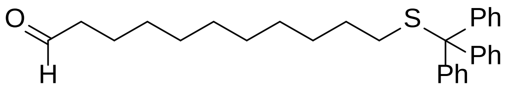

According to the general procedure, from 11-triphenylmercapto-1-ol (**3**) (1.24 mmol, 553 mg), triethylamine (600 µL, 4.34 mmol) and sulfur trioxide pyridine complex (408 mg, 2.57 mmol) in a mixture of DCM (3.5 mL) and DMSO (500 µL), the compound **6** was obtained as a transparent oil (yield, 68%). **^{1}H-NMR** (400 MHz, CD_2Cl_2) *δ*/ppm: 9.77 (t, *J* = 1.8 Hz, 1H), 7.50 – 7.41 (m, 6H), 7.33 (t, *J* = 7.5 Hz, 6H), 7.26 (t, *J* = 7.2 Hz, 3H), 2.43 (td, *J* = 7.4, 1.8 Hz, 2H), 2.18 (t, *J* = 7.4 Hz, 2H), 1.64 (q, *J* = 7.3 Hz, 2H), 1.43 (q, *J* = 7.3 Hz, 2H), 1.38 – 1.12 (m, 12H). **^{13}C-NMR** (101 MHz, CD_2Cl_2) δ/ppm: 202.64, 145.15, 129.57, 127.75, 126.47, 66.30, 53.41, 43.85, 31.89, 29.30, 29.12, 29.09, 28.98, 28.56, 22.06; **FT-IR** *(ν*/cm^{-1}): 3058.1, 2924.9, 2853.2, 1723.2, 1595.5, 1489.3, 1444.1, 1276.2, 1262.1, 1081.4, 1033.4, 908.6, 851.1, 743.3; **EM (m/z)**: (MALDI-TOF): calculated for $C_{30}H_{36}OS$: 444.25; found: 445.43 (M+1H) and 243.21 ($\cdot CPh_3$).

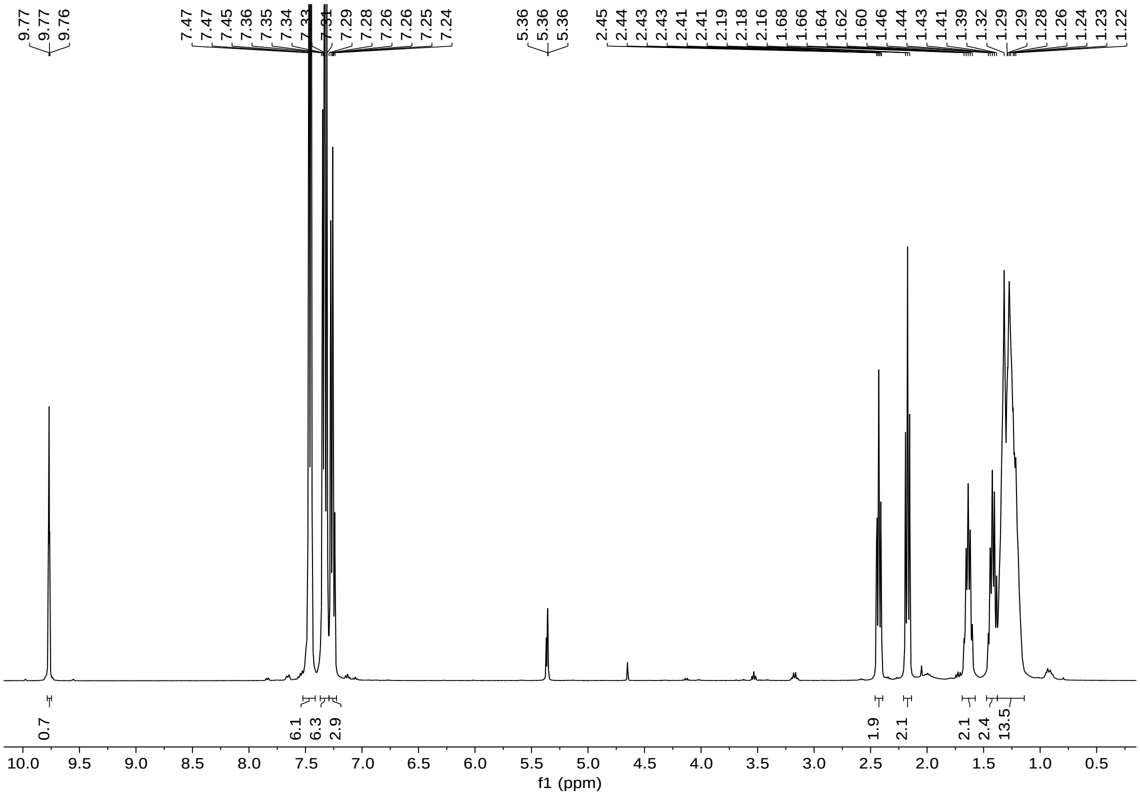
9.77
9.77
9.76
7.47
7.47
7.45
7.36
7.35
7.34
7.33
7.31
7.29
7.28
7.26
7.26
7.25
7.24
5.36
5.36
5.36
2.45
2.44
2.43
2.43
2.41
2.41
2.19
2.18
2.16
1.68
1.66
1.64
1.62
1.60
1.46
1.44
1.43
1.41
1.39
1.32
1.29
1.29
1.28
1.26
1.24
1.23
1.22
0.7
6.1
6.3
2.9
1.9
2.1
2.1
2.4
13.5
10.0 9.5 9.0 8.5 8.0 7.5 7.0 6.5 6.0 5.5 5.0 4.5 4.0 3.5 3.0 2.5 2.0 1.5 1.0 0.5
f1 (ppm)

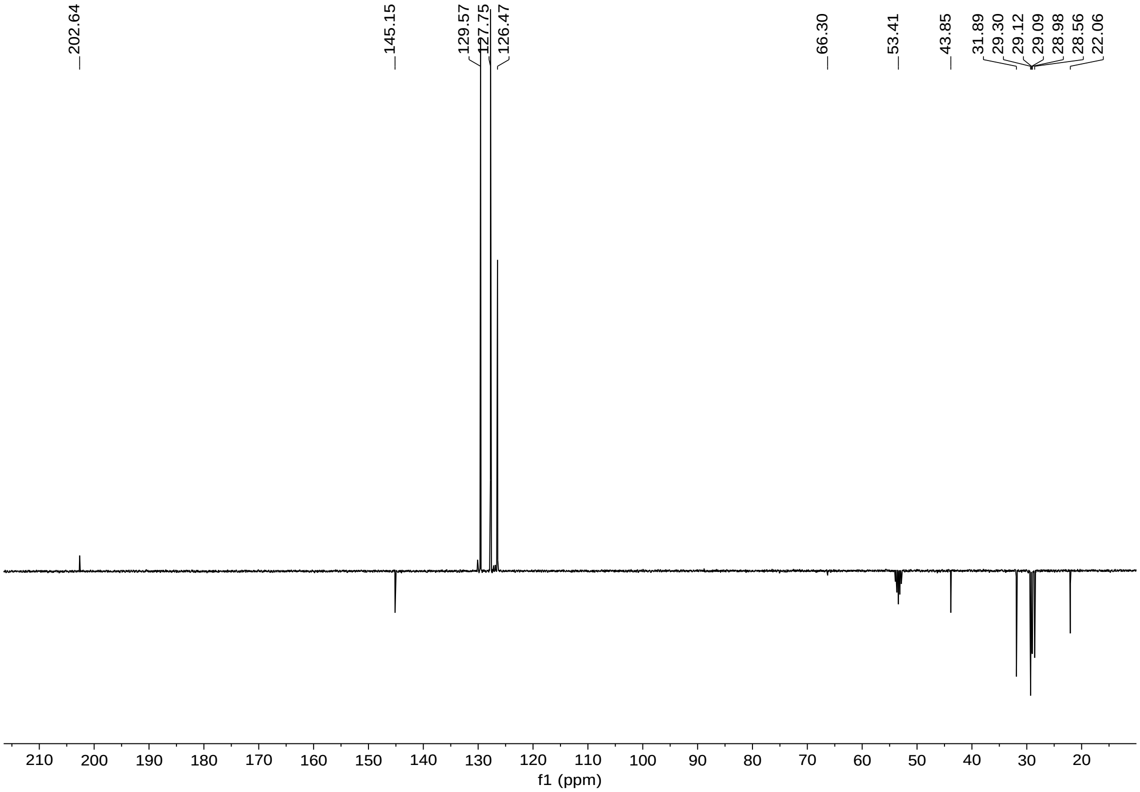
202.64
145.15
129.57
127.75
126.47
66.30
53.41
43.85
31.89
29.30
29.12
29.09
28.98
28.56
22.06
210 200 190 180 170 160 150 140 130 120 110 100 90 80 70 60 50 40 30 20
f1 (ppm)

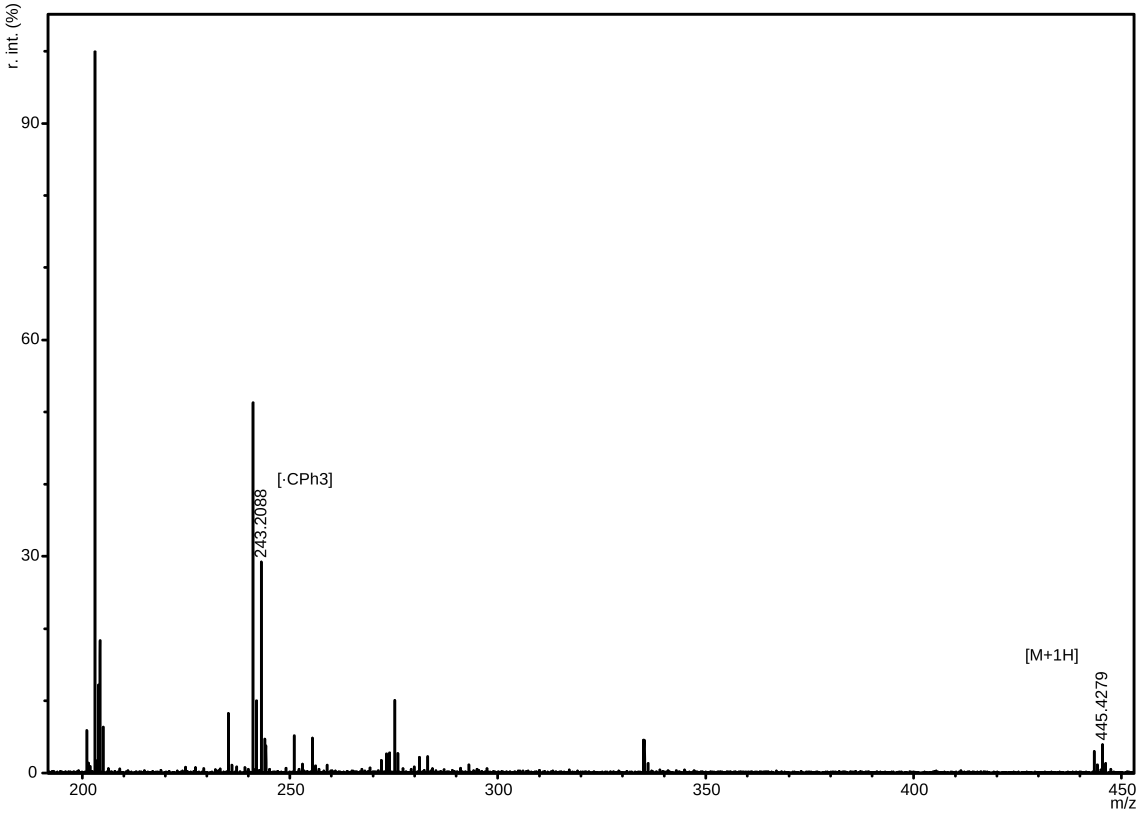

(E)-(8-(4-(bis(perchlorophenyl)methyl)-2,3,5,6-tetrachlorophenyl)oct-7-en-1-yl)(trityl)sulfane (7)

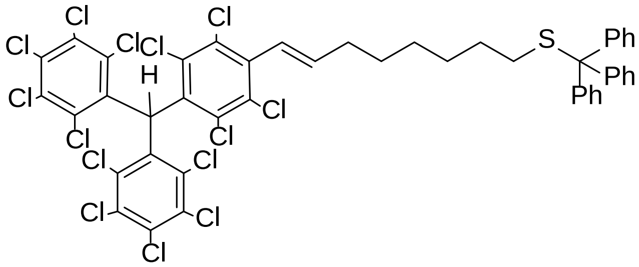

According to the general procedure, from potassium tert-butoxide (58 mg 051 mmol), PTM-P(O)(OEt)$_2$ (300 mg, 0.34 mmol) and 7-(tritylthio)heptanal (**4**) (263 mg, 0.68 mmol) in THF (10 mL), the compound **7** was obtained as a withe powder (yield, 67%). **^{1}H-NMR** (400 MHz, CD_2Cl_2) δ/ppm: 7.49 – 7.42 (m, 6H), 7.32 (t, J = 7.5 Hz, 6H), 7.25 (t, J = 7.2 Hz, 3H), 7.06 (s, 1H), 6.35 (d, J = 16.1 Hz, 1H), 6.16 (dt, J = 16.1, 6.9 Hz, 1H), 2.29 (q, J = 6.8 Hz, 2H), 2.19 (t, J = 7.3 Hz, 2H), 1.53 – 1.20 (m, 8H);); **^{13}C-NMR** (101 MHz, CD_2Cl_2) δ/ppm: 145.05, 141.22, 138.14, 136.67, 136.64, 135.38, 135.07, 134.96, 134.44, 133.95, 133.94, 133.56, 133.41, 133.38, 133.34, 133.23, 132.35, 132.31, 132.14, 129.49, 127.68, 126.40, 124.21, 66.27, 56.53, 53.33, 32.98, 31.77, 28.66, 28.46, 28.41, 28.32; **FT-IR** (ν/cm^{-1}): 3057.4, 2926.1, 2852.6, 1594.5, 1488.4, 1442.7, 1362.6, 1334.9, 1295.2, 1238.3, 1137.8, 1238.3, 1137.8, 1032.6, 966.2, 852.7,

807.2, 741.5, 696.9. **EM (m/z)** (ESi): calculated for $C_{46}H_{30}Cl_{14}S$: 1109.76; found: 1108.8 (M-1H).

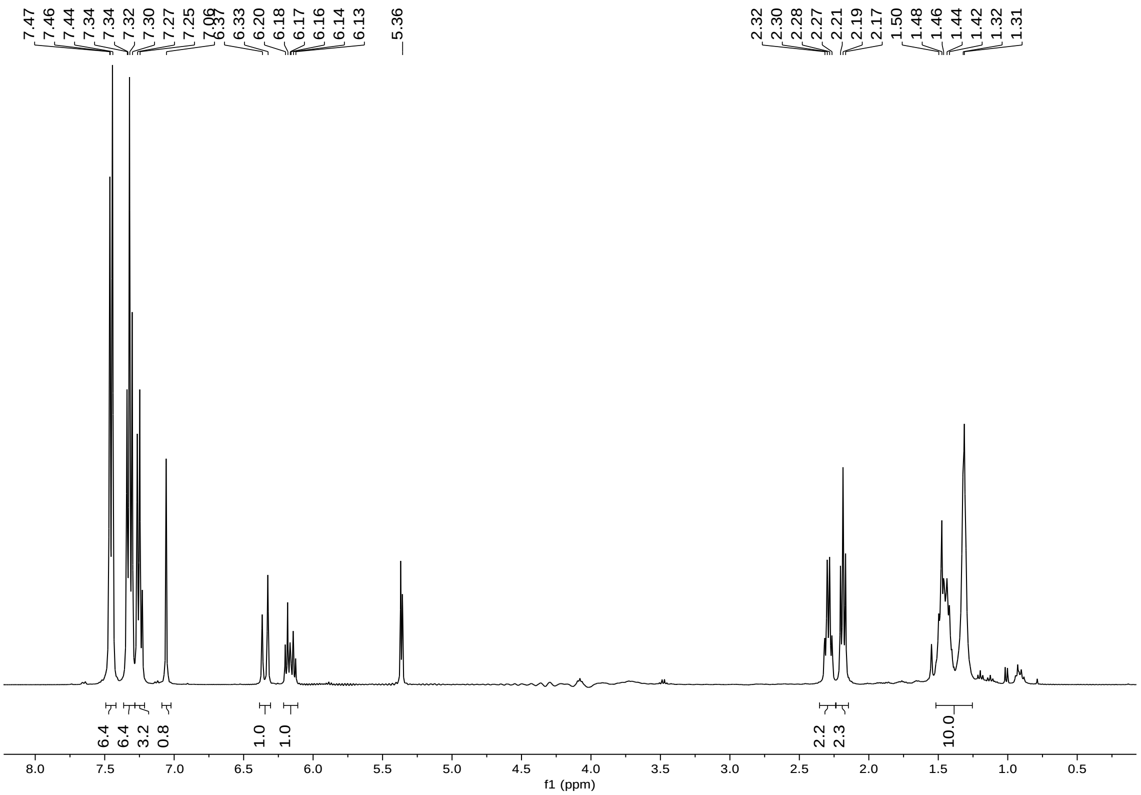

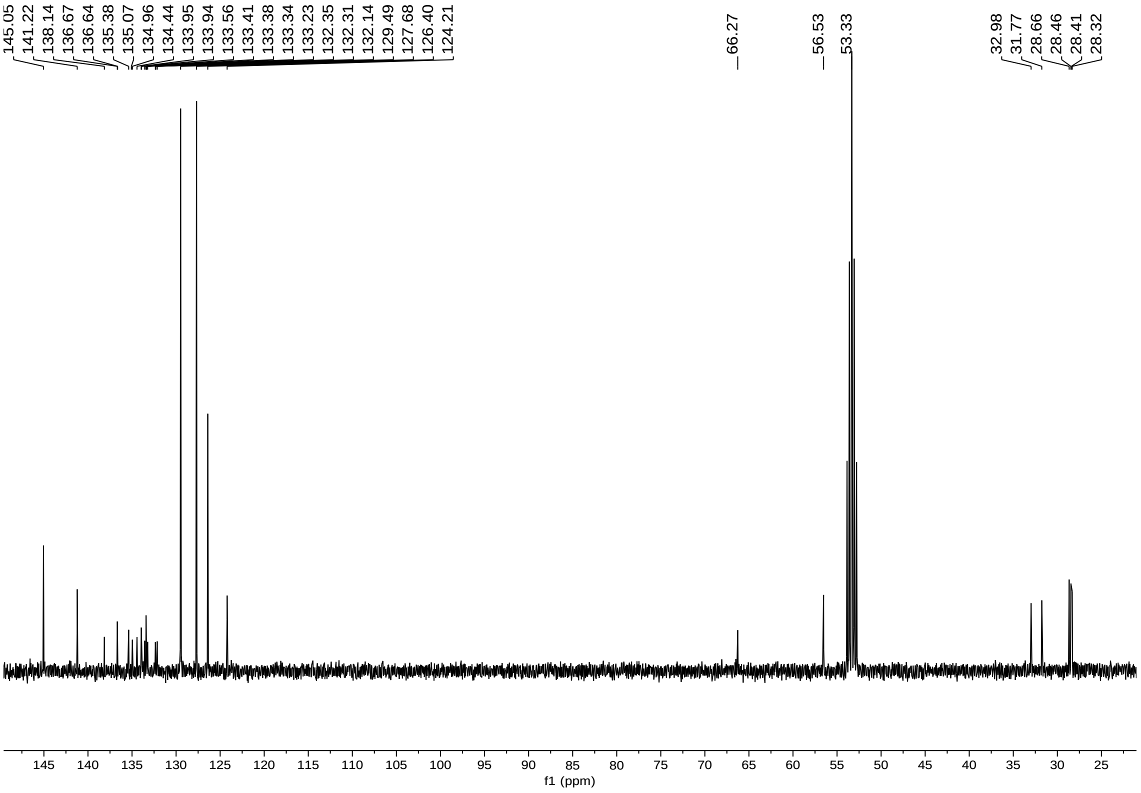

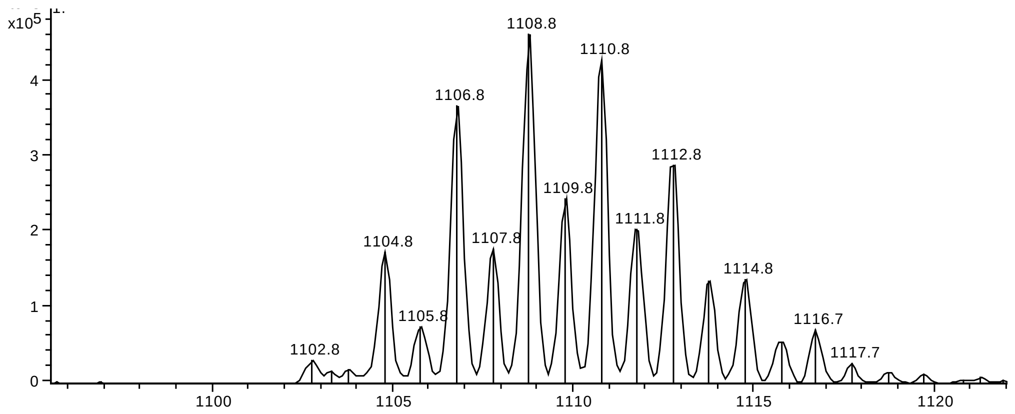

(E)-(10-(4-(bis(perchlorophenyl)methyl)-2,3,5,6-tetrachlorophenyl)dec-9-en-1-yl)(trityl)sulfane (8)

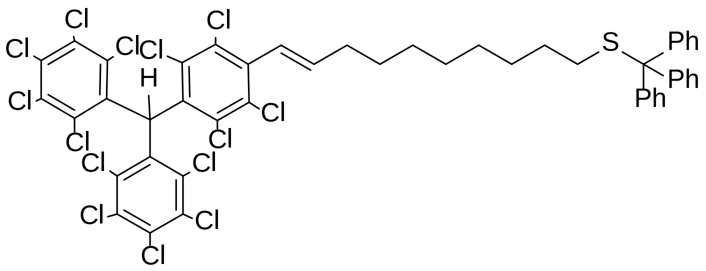

According to the general procedure, from potassium tert-butoxide (58 mg 051 mmol), PTM-P(O)(OEt)$_2$ (300 mg, 0.34 mmol) and 9-(tritylthio)nonanal (**5**) (283 mg, 0.68 mmol) in THF (10 mL), the compound **8** was obtained as a withe powder (yield, 65%).^{1}**H-NMR** (400 MHz, CD_2Cl_2) δ/ppm: 7.48 – 7.41 (m, 6H), 7.32 (t, J = 7.5 Hz, 6H), 7.25 (t, J = 7.2 Hz, 3H), 7.05 (s, 1H), 6.36 (d, J = 16.1 Hz, 1H), 6.19 (dt, J = 16.1, 6.9 Hz, 1H), 2.32 (q, J = 6.8 Hz, 2H), 2.16 (t, J = 7.4 Hz, 2H), 1.52 (p, J = 7.2 Hz, 2H), 1.46 – 1.16 (m, 10H); ^{13}**C-NMR** (101 MHz, CD_2Cl_2) δ/ppm: 145.13, 141.46, 138.25, 136.74, 136.70, 135.42, 135.14, 135.03, 134.53, 134.50, 134.02, 134.00, 133.62, 133.47, 133.44, 133.40, 133.30, 132.41, 132.37, 132.21, 129.56, 127.75, 126.47, 124.21, 66.29, 56.59, 53.42, 33.18, 31.86, 29.17, 29.07, 28.96, 28.92, 28.61, 28.55; **FT-IR** (ν/cm^{-1}): 3061.7, 2923.7, 2851.4, 1593.4, 1488.3, 1443.0, 1365.1, 1334.7, 1295.9, 1238.3, 1137.9, 1033.1, 965.1, 852.9, 807.5, 741.5, 967.7. **EM (m/z)** (ESI): calculated for $C_{48}H_{34}Cl_{14}S$: 1137.79; found: 1136.8 (M-1H).

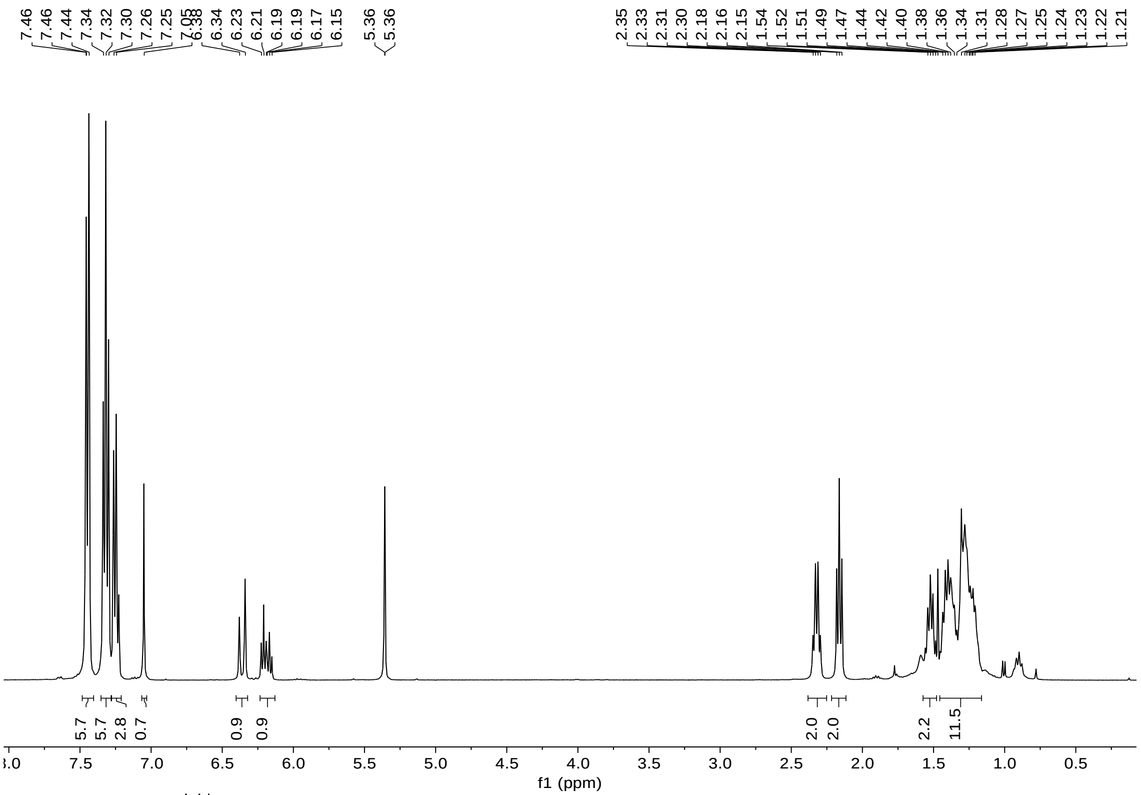
7.46 7.46 7.44 7.34 7.32 7.30 7.26 7.25 7.05 6.38 6.34 6.23 6.21 6.19 6.19 6.17 6.15 5.36 5.36
2.35 2.33 2.31 2.30 2.18 2.16 2.15 1.54 1.52 1.51 1.49 1.47 1.44 1.42 1.40 1.38 1.36 1.34 1.31 1.28 1.27 1.25 1.24 1.23 1.22 1.21
5.7 5.7 2.8 0.7 0.9 0.9 2.0 2.0 2.2 11.5
f1 (ppm)

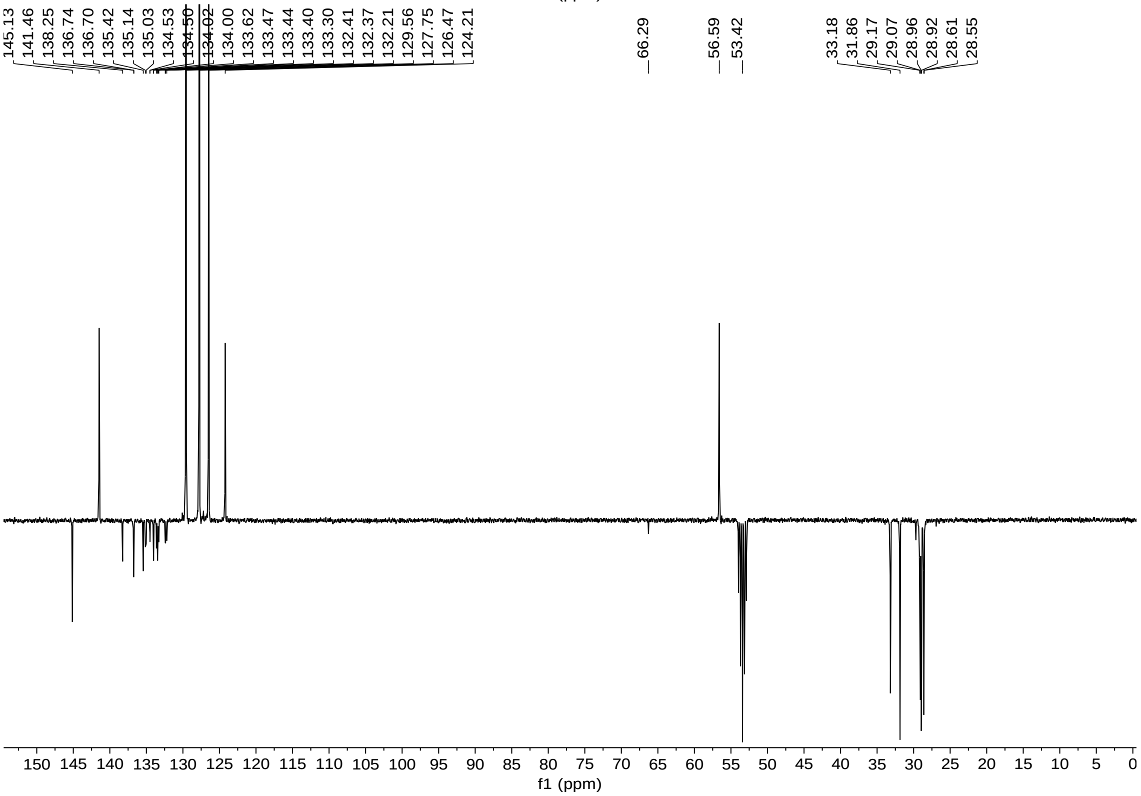
145.13 141.46 138.25 136.74 136.70 135.42 135.14 135.03 134.53 134.50 134.02 134.00 133.62 133.47 133.44 133.40 133.30 132.41 132.37 132.21 129.56 127.75 126.47 124.21
66.29
56.59 53.42
33.18 31.86 29.17 29.07 28.96 28.92 28.61 28.55
f1 (ppm)

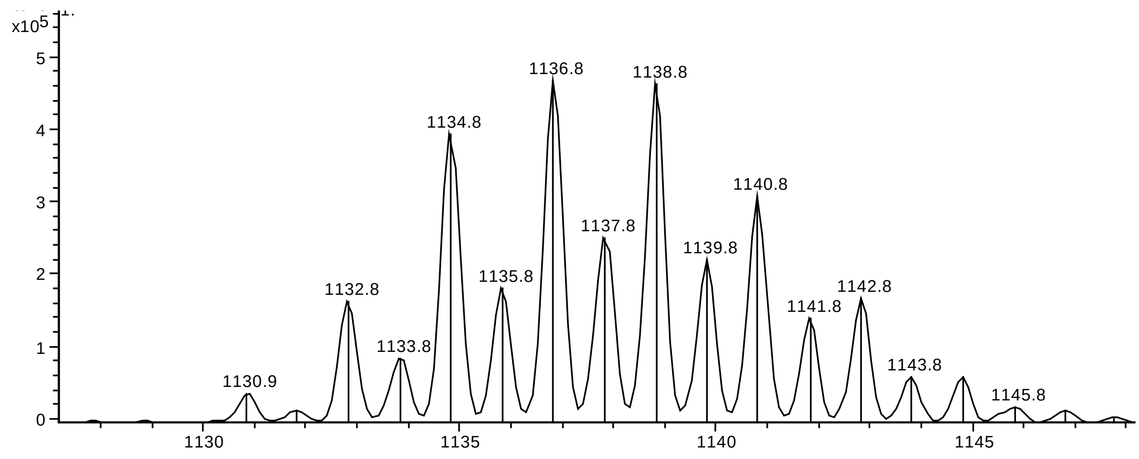

(E)-(12-(4-(bis(perchlorophenyl)methyl)-2,3,5,6-tetrachlorophenyl)dodec-11-en-1-yl)(trityl)sulfane (9)

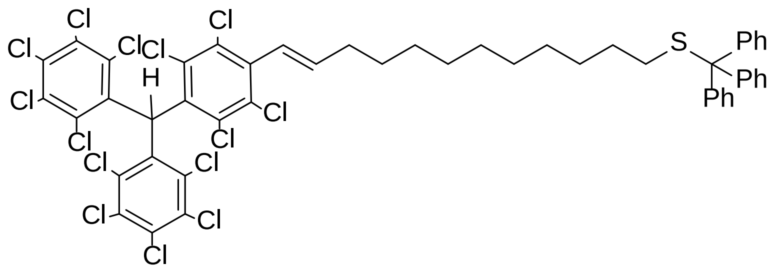

According to the general procedure, from potassium tert-butoxide (58 mg 051 mmol), PTM-P(O)(OEt)$_2$ (300 mg, 0.34 mmol) and 11-(tritylthio)undecanal (**6**) (302mg, 0.68 mmol) in THF (10 mL), the compound **9** was obtained as a withe powder (yield, 71%). **^{1}H-NMR** (400 MHz, CD_2Cl_2) *δ*/ppm: 7.45 (d, *J* = 7.6 Hz, 6H), 7.32 (t, *J* = 7.5 Hz, 6H), 7.25 (t, *J* = 7.2 Hz, 3H), 7.05 (s, 1H), 6.37 (d, *J* = 16.1 Hz, 1H), 6.20 (dt, *J* = 16.1, 6.8 Hz, 1H), 2.33 (q, *J* = 6.8 Hz, 2H), 2.16 (t, *J* = 7.4 Hz, 2H), 1.55 (p, *J* = 7.3 Hz, 2H), 1.48 – 1.11 (m, 14H); **^{13}C-NMR** (101 MHz, CD_2Cl_2) δ/ppm: 145.13, 141.51, 138.26, 136.74, 136.70, 135.41, 135.14, 135.02, 134.50, 134.01, 134.00, 133.62, 133.46, 133.43, 133.40, 133.29, 132.41, 132.37, 132.21, 129.55, 127.74, 126.46, 124.18, 66.27, 53.42, 33.21, 31.87, 29.47, 29.37, 29.11, 29.02, 28.98, 28.67, 28.55. **EM (m/z)** (ESI): calculated for $C_{50}H_{38}Cl_{14}S$: 1165.82; found: 1164.8 (M-1H).

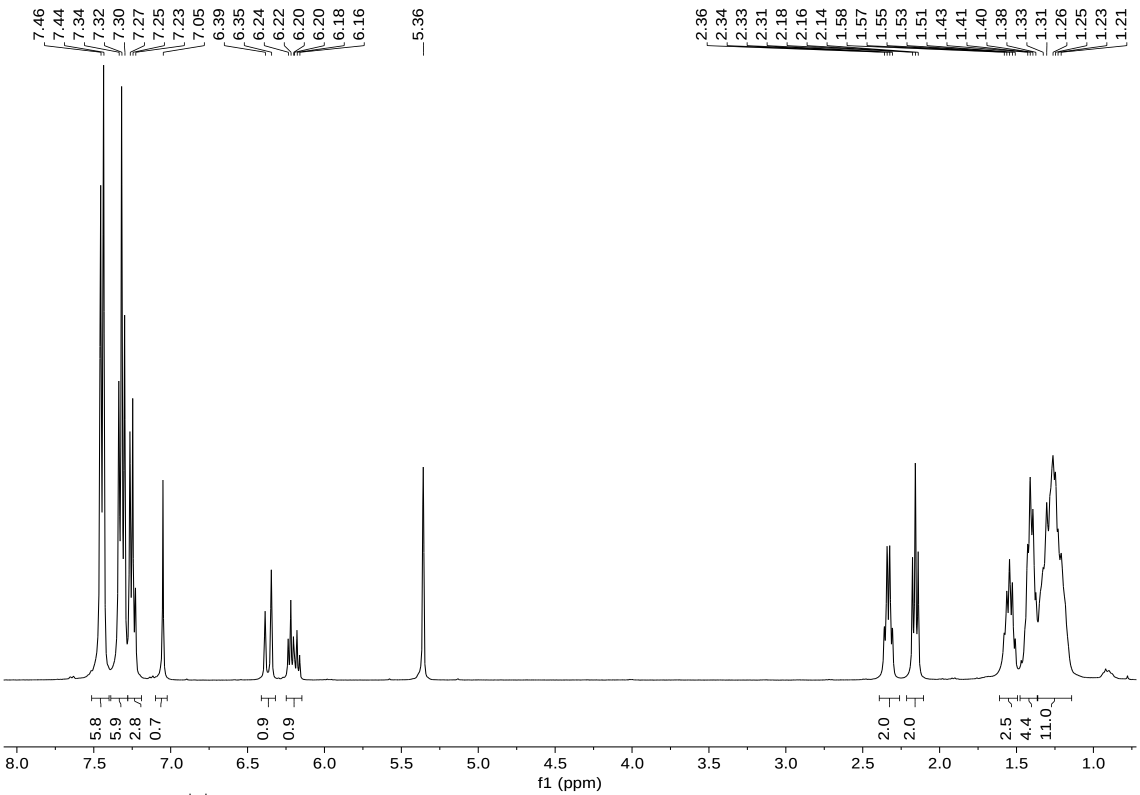

7.46
7.44
7.34
7.32
7.30
7.27
7.25
7.23
7.05
6.39
6.35
6.24
6.22
6.20
6.20
6.18
6.16
5.36
2.36
2.34
2.33
2.31
2.18
2.16
2.14
1.58
1.57
1.55
1.53
1.51
1.43
1.41
1.40
1.38
1.33
1.31
1.26
1.25
1.23
1.21
5.8
5.9
2.8
0.7
0.9
0.9
2.0
2.0
2.5
4.4
11.0
8.0 7.5 7.0 6.5 6.0 5.5 5.0 4.5 4.0 3.5 3.0 2.5 2.0 1.5 1.0
f1 (ppm)

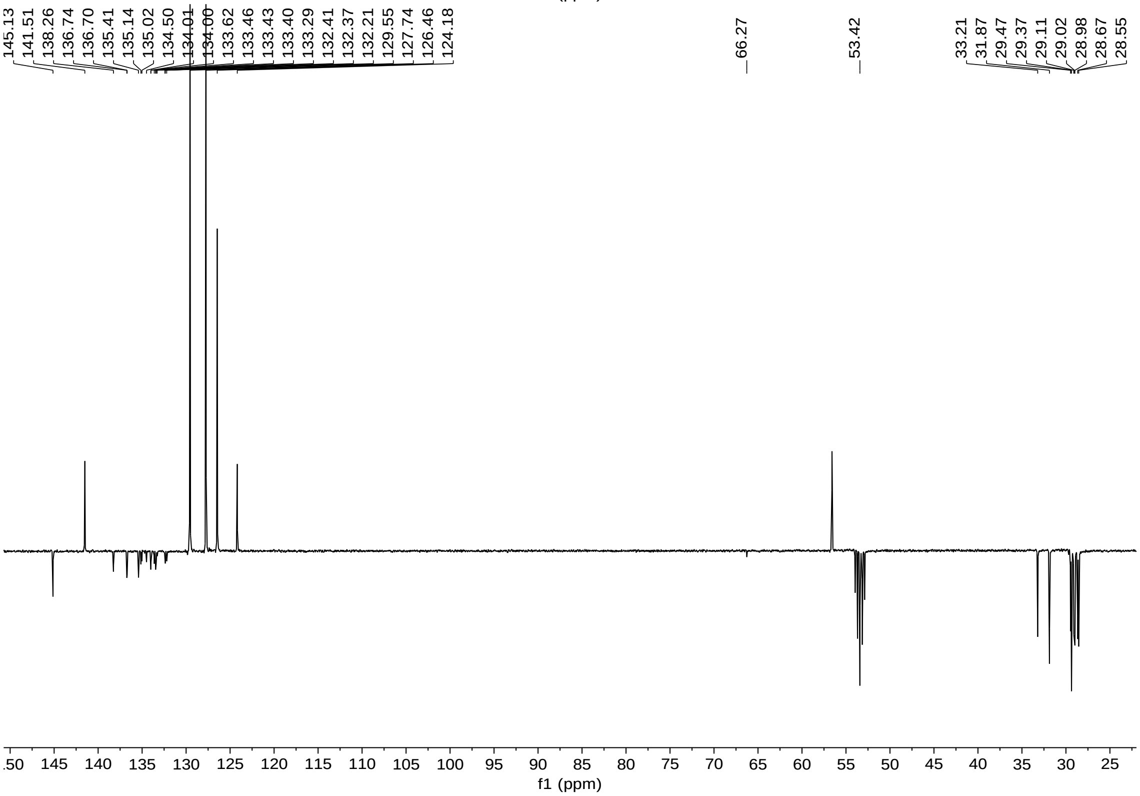

145.13
141.51
138.26
136.74
136.70
135.41
135.14
135.02
134.50
134.01
134.00
133.62
133.46
133.43
133.40
133.29
132.41
132.37
132.21
129.55
127.74
126.46
124.18
66.27
53.42
33.21
31.87
29.47
29.37
29.11
29.02
28.98
28.67
28.55
150 145 140 135 130 125 120 115 110 105 100 95 90 85 80 75 70 65 60 55 50 45 40 35 30 25
f1 (ppm)

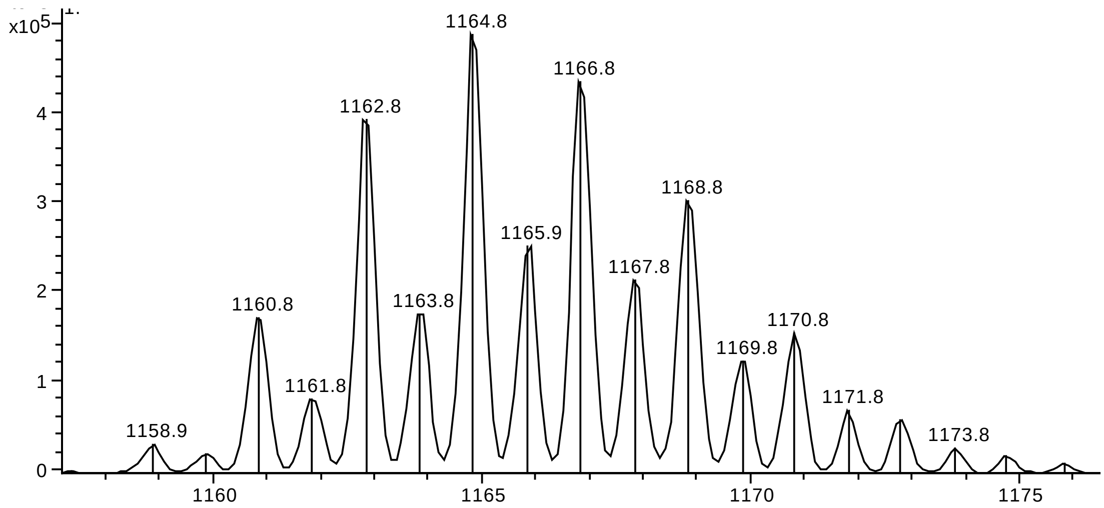

(E)-(8-(4-(bis(perchlorophenyl)methyl)-2,3,5,6-tetrachlorophenyl)oct-7-en-1-yl)(trityl)sulfane (radical) (10)

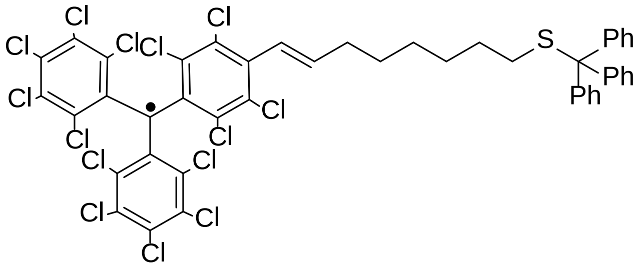

According to the general procedure, the compound **10** was obtained as a red powder in quantitative yield. **HPLC**: retention time: 14.9 (2.5% of cis isomer), 15.5min (95% of trans isomer), 16.9 (2.5% αH); **UV/Vis** (CH_2Cl_2): *λ(nm)* (log*ε*)= 388 (4.46), 515 (3.08), 567 (3.10). **CV**: $E^{1/2}$ = -0.16 V (PTM reduction); **FT-IR** (ν/cm^{-1}): 3057.1, 2924.2, 2853.2, 1653.5, 1596.1, 1489.5, 144.4, 1333.0, 1259.6, 1157.3, 1081.6, 1033.9, 857.7, 816.4, 738.1, 697.9, 652.3, 620.5; **EPR** (CH_2Cl_2, r.t.): g = 2.002804; ΔH_{PP} = 1.3 G, $a_{13C\alpha}$ = 29.6 G; a_{13Co} = 13.2 G; a_{13Cm} = 10.3 G; a_{1H} = 1.8 G; **EM (m/z)** (ESI): calculated for $C_{46}H_{29}Cl_{14}S^{\cdot}$: 1108.75; found: 1108.8 (M*).

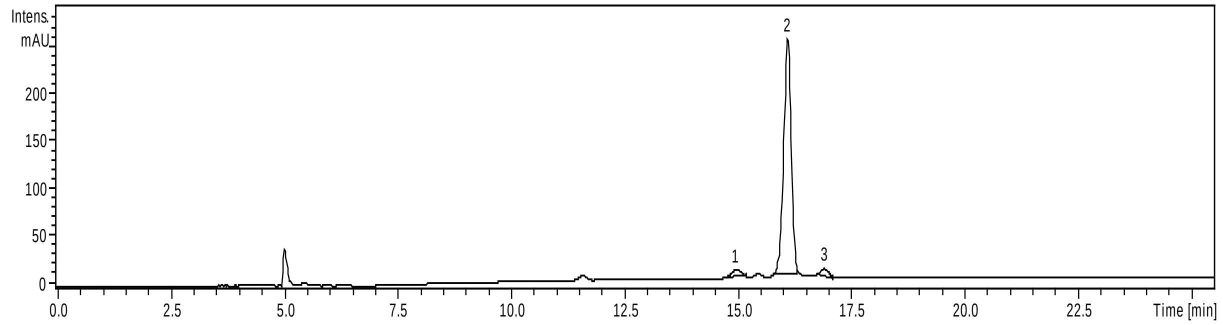

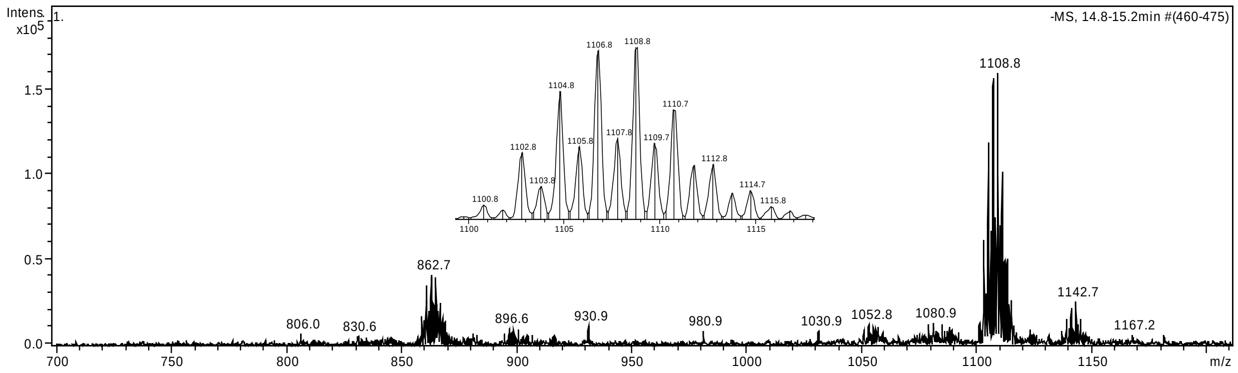

(E)-(10-(4-(bis(perchlorophenyl)methyl)-2,3,5,6-tetrachlorophenyl)dec-9-en-1-yl)(trityl)sulfane (radical) (11)

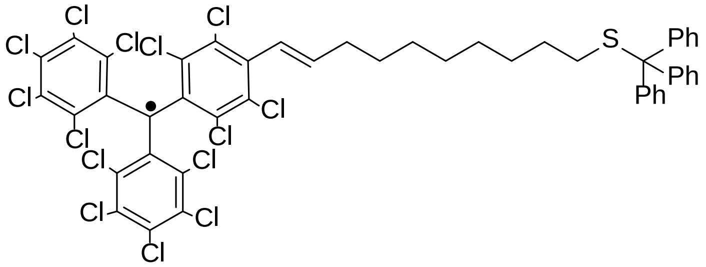

According to the general procedure, the compound **11** was obtained as a red powder in quantitative yield. **HPLC**, retention time: 17.4min (98% of trans isomer); **UV/Vis** (CH_2Cl_2): $\lambda(nm)$ (logε)= 387 (4.49), 515 (3.11), 566 (3.13); **CV**: $E^{1/2}$ = -0.16 V (PTM reduction); **FT-IR** (ν/cm^{-1}): 3056.0, 2924.2, 2852.4, 1649.5, 1595.2, 1489.1, 1443.4, 1332.7, 1259.2, 1157.2, 1033.6, 966.06, 858.7, 815.9, 739.7, 697.8, 651.9, 619.7; **EPR** (CH_2Cl_2, r.t.): g = 2.002840; ΔH_{PP} = 1.1 G, $a_{13C\alpha}$ = 29.6 G; a_{13Co} = 13.2 G; a_{13Cm} = 10.2 G; a_{1H} = 1.8 G; **EM (m/z)** (Electrospray): calculated for $C_{48}H_{33}Cl_{14}S^{\cdot}$: 1136.78; found: 1136.8 (M*).

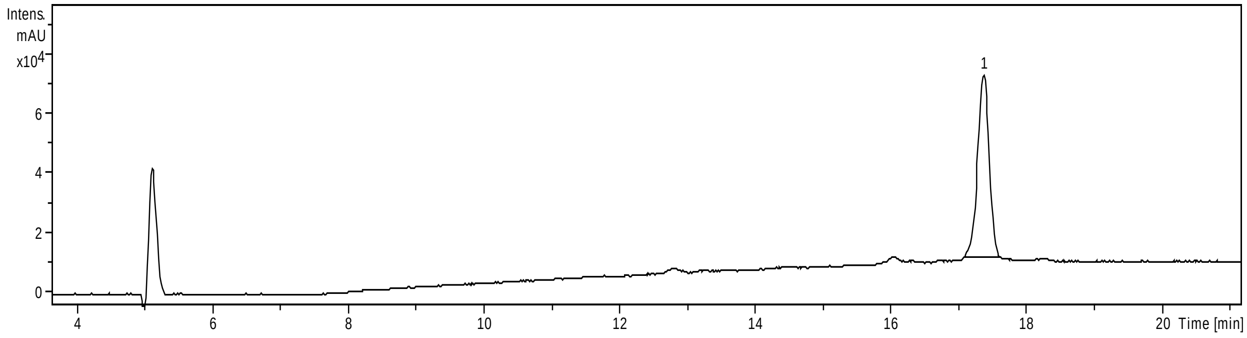

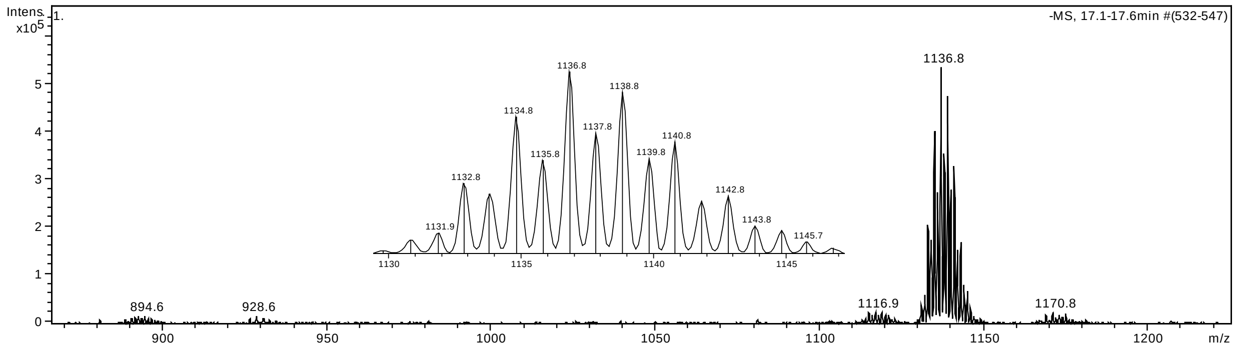

(E)-(12-(4-(bis(perchlorophenyl)methyl)-2,3,5,6-tetrachlorophenyl) dodec-11-en-1-yl)(trityl)sulfane (radical) (12)

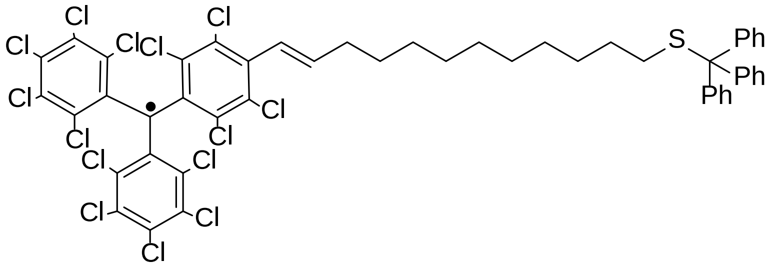

According to the general procedure, the compound **12** was obtained as a red powder in quantitative yield. **HPLC**, retention time: 18.8min (96% of trans isomer); **UV/Vis** (CH_2Cl_2): *λ(nm)* (log*ε*)= 388 (4.49), 515 (3.12), 566 (3.14); **CV**: $E^{1/2}$ = -0.16 V (PTM reduction); **FT-IR** (ν/cm^{-1}):3055.7, 2924.2, 2852.3 1658.5, 1595.6, 1489.5, 1444.0, 1332.8, 1259.4, 1156.9, 1080.9, 1033.4, 967.2, 858.5, 816.0, 738.6, 697.8, 673.2, 651.8, 620.1; **EPR** (CH_2Cl_2, r.t.): g = 2.002945; ΔH_{PP} = 1.4 G, $a_{13C\alpha} = 29.6$ G; $a_{13Co} = 13.2$ G; $a_{13Cm} = 10.1$ G; $a_{1H} = 1.8$ G; **EM (m/z)** (Electrospray): calculated for $C_{48}H_{32}Cl_{14}S^{\cdot}$: 1164.81; found: 1164.8 (M*).

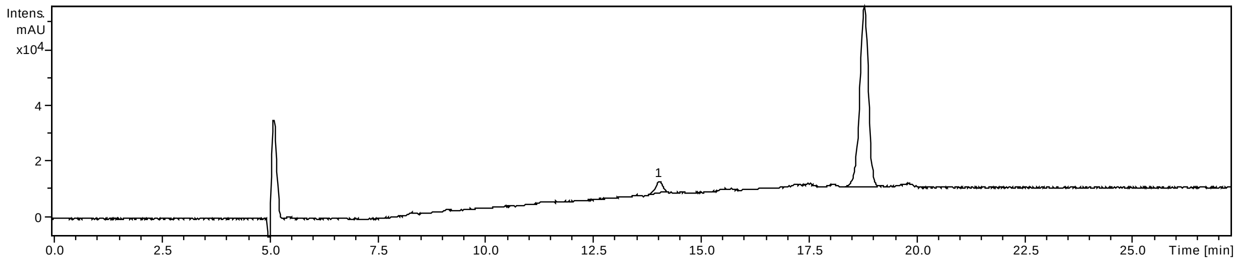

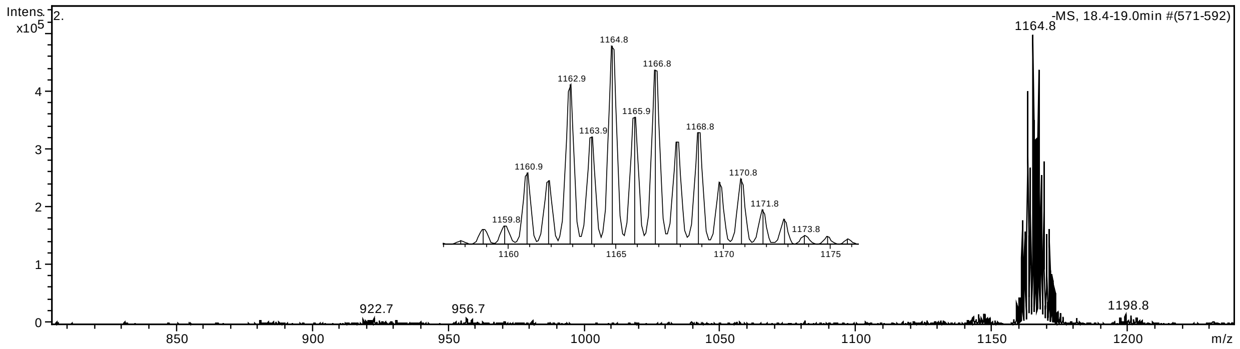

(E)-8-(4-(bis(perchlorophenyl)methyl)-2,3,5,6-tetrachlorophenyl)oct-7-ene-1-thiol (NR8)

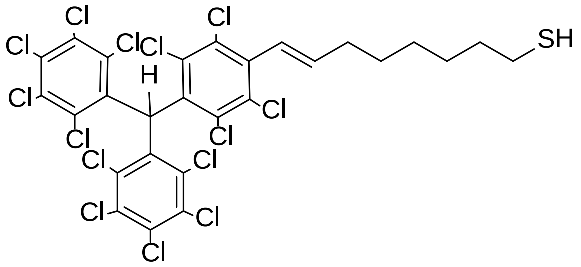

The compound **NR8** was obtained as withe powder in quantitative yield, using the general methodology described for the generation of free thiols. **^{1}H-NM**R (400 MHz, CD_2Cl_2) *δ*/ppm: 7.00 (s, 1H), 6.31 (d, *J* = 16.1 Hz, 1H), 6.14 (dt, *J* = 15.7, 6.7 Hz, 1H), 2.54 (q, J = 7.2 Hz, 2H), 2.31 (q, *J* = 6.3 Hz, 2H), 1.71 – 1.17 (m, 9H); **^{13}C-NMR** (101 MHz, $CDCl_3$) δ/ppm: 141.27, 138.19, 136.79, 136.76, 135.64, 135.28, 135.16, 134.71, 134.10, 134.08, 133.78, 133.70, 133.67, 133.64, 133.58, 133.41, 132.58, 132.54, 132.32, 124.55, 77.16, 56.71, 34.10, 33.33, 28.68, 28.63, 28.30, 24.77. **FT-IR** (*ν*/cm^{-1}): 2927.0, 2853.9, 2343.8, 1654.2, 1533.8, 1461.7, 1363.7, 1335.5, 1298.7, 1241.0, 1139.2, 963.9, 855.3, 807.2, 752.8, 717.0, 675.2, 692.5. **EM (m/z)** (MALDI-TOF): calculated for $C_{27}H_{16}Cl_{14}S$: 867.65; found: 866.90 (M-1H) and 796.95 (M-2Cl).

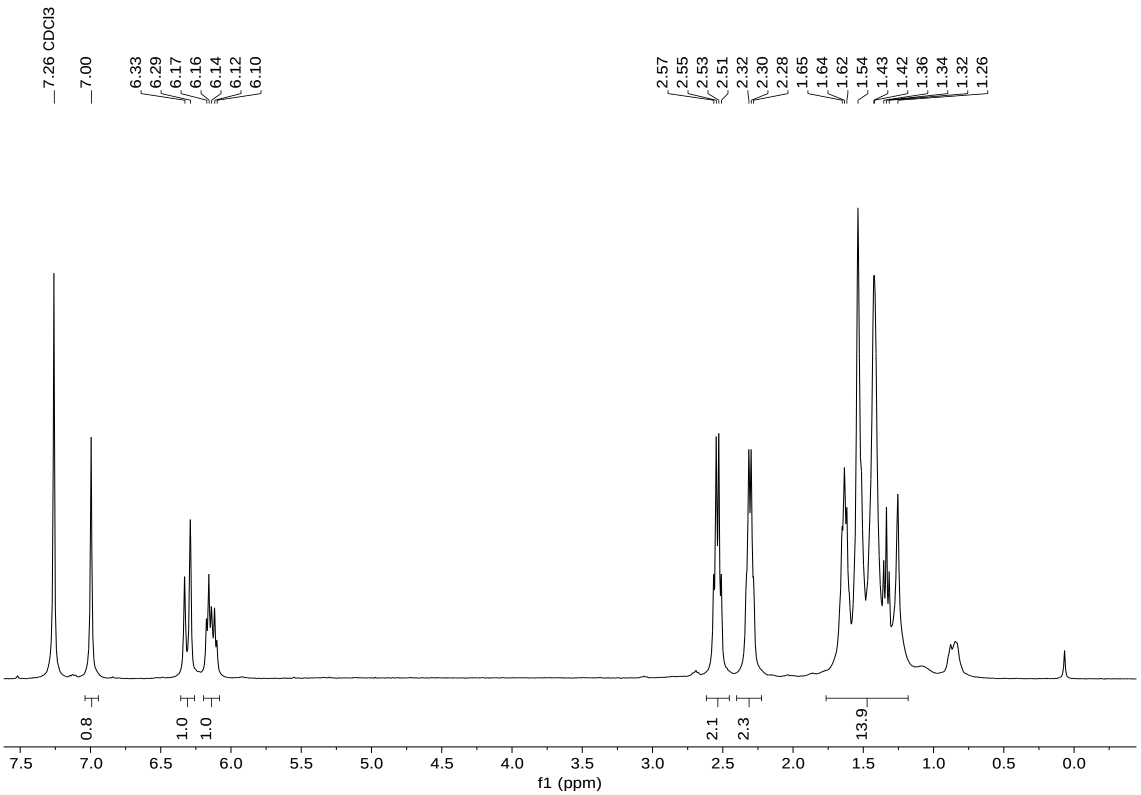
7.26 CDCl3
7.00
6.33
6.29
6.17
6.16
6.14
6.12
6.10
2.57
2.55
2.53
2.51
2.32
2.30
2.28
1.65
1.64
1.62
1.54
1.43
1.42
1.36
1.34
1.32
1.26
0.8
1.0
1.0
2.1
2.3
13.9
f1 (ppm)

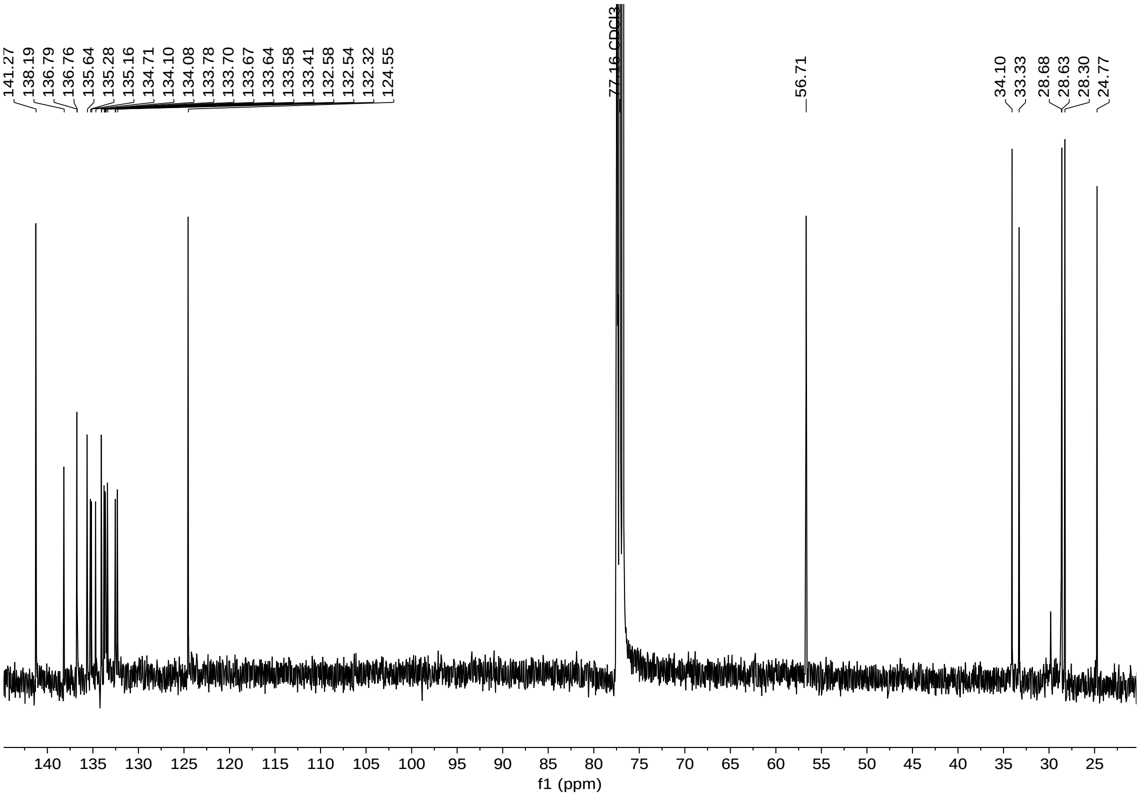
141.27
138.19
136.79
136.76
135.64
135.28
135.16
134.71
134.10
134.08
133.78
133.70
133.67
133.64
133.58
133.41
132.58
132.54
132.32
124.55
77.16 CDCl3
56.71
34.10
33.33
28.68
28.63
28.30
24.77
f1 (ppm)

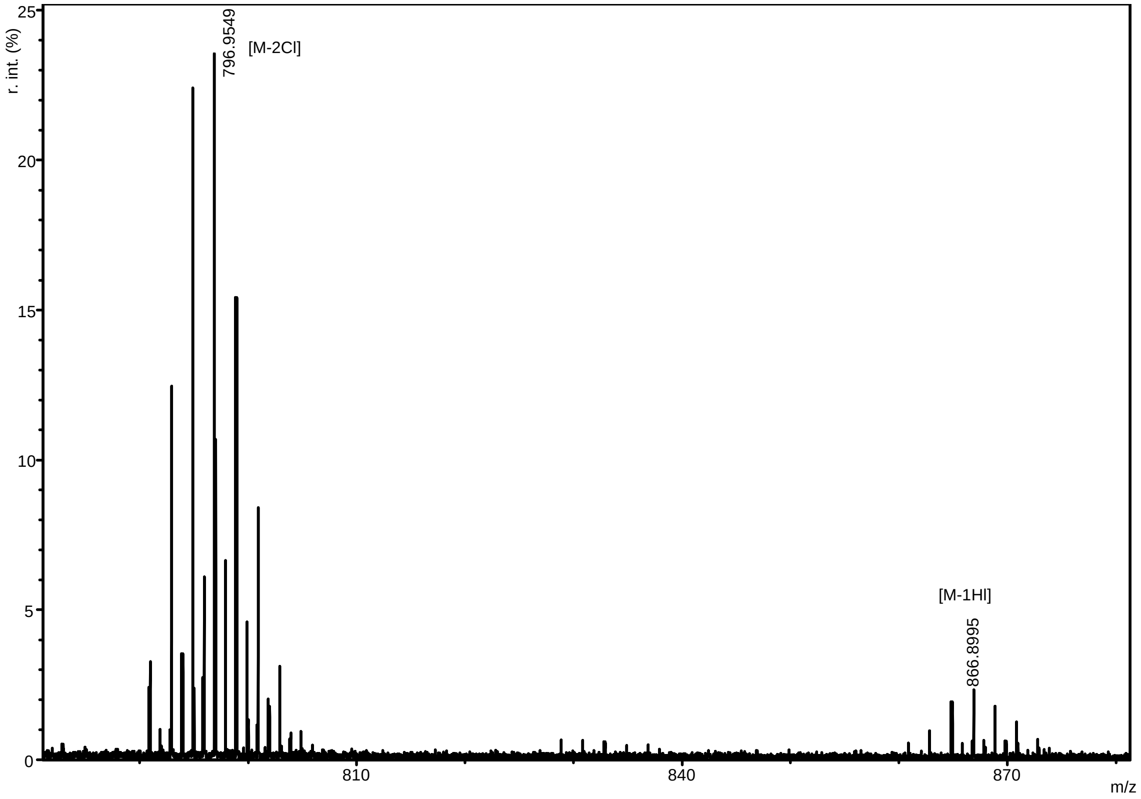

(E)-10-(4-(bis(perchlorophenyl)methyl)-2,3,5,6-tetrachlorophenyl)dec-9-ene-1-thiol (NR10).

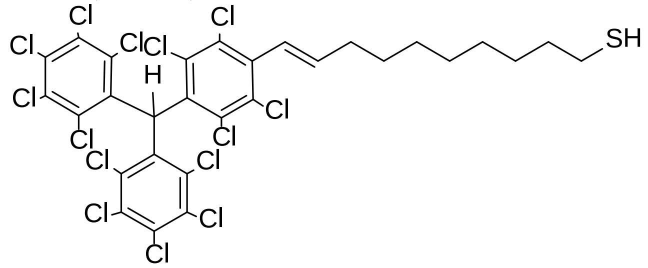

The compound **NR10** was obtained as withe powder in quantitative yield, using the general methodology described for the generation of free thiols. **^{1}H-NM**R (400 MHz, $CDCl_3$) *δ*/ppm: 6.99 (s, 1H), 6.31 (d, *J* = 16.1 Hz, 1H), 6.14 (dt, *J* = 16.1, 6.8 Hz, 1H), 2.52 (q, *J* = 7.4 Hz, 2H), 2.30 (q, *J* = 6.8 Hz, 2H), 1.61 (p, *J* = 7.2 Hz, 2H), 1.51 (p, *J* = 7.2 Hz, 2H), 1.43 – 1.21 (m, 9H); **^{13}C-NMR** (101 MHz, $CDCl_3$) δ/ppm: 141.47, 136.80, 136.77, 135.59, 135.28, 135.16, 134.70, 134.10, 134.08, 133.77, 133.69, 133.67, 133.64, 133.57, 132.58, 132.53, 132.32, 124.43, 77.16, 56.70, 34.17, 33.41, 29.86, 29.45, 29.18, 29.12, 28.79, 28.50, 24.81.; **FT-IR** (*ν*/cm^{-1}): 2926.5, 2854.0, 1710.0, 1653.9, 1461.1, 1365.2, 1337.0, 1296.8, 1239.7, 1193.0, 1137.7, 1042.9, 966.8, 899.9, 855.2, 808.4, 739.0, 690.1, 648.4, 609.9; **EM (m/z)** (MALDI-TOF): calculated for $C_{29}H_{20}Cl_{14}S$: 895.68; found: 894.97 (M-1H) and 825.02 (M-2Cl).

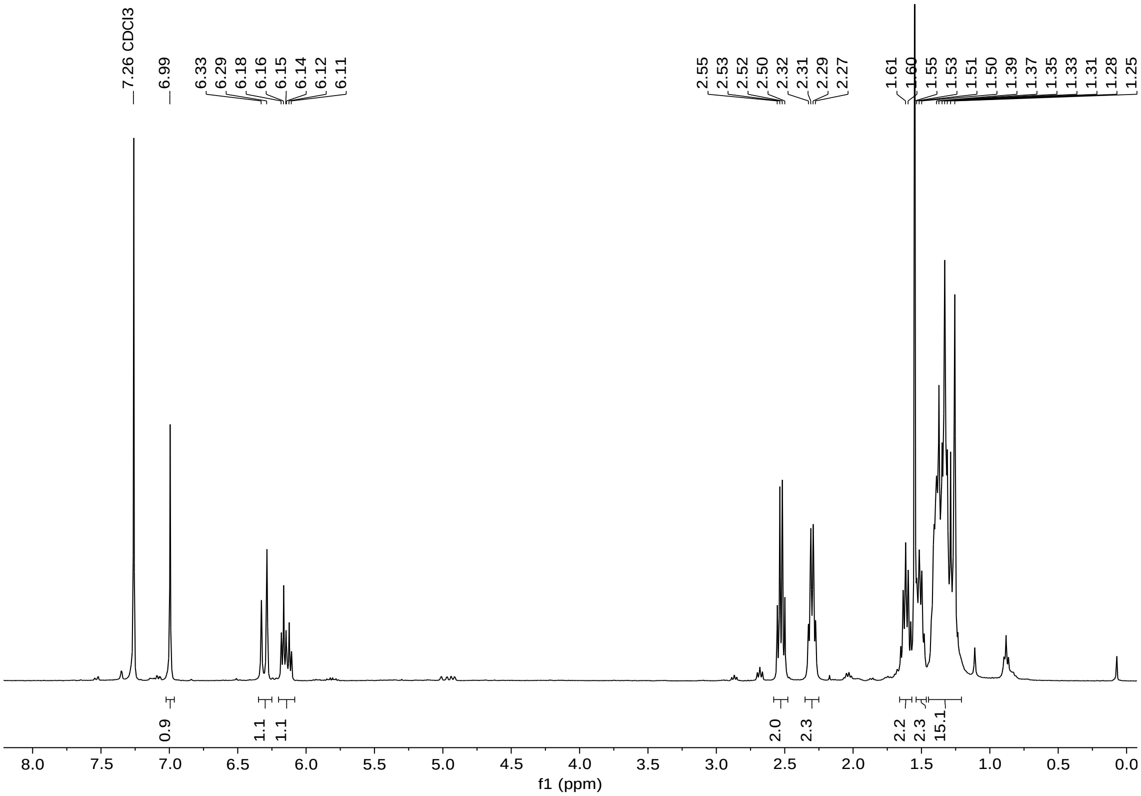
7.26 CDCl3
6.99
6.33
6.29
6.18
6.16
6.15
6.14
6.12
6.11
2.55
2.53
2.52
2.50
2.32
2.31
2.29
2.27
1.61
1.60
1.55
1.53
1.51
1.50
1.39
1.37
1.35
1.33
1.31
1.28
1.25
0.9
1.1
1.1
2.0
2.3
2.2
2.3
15.1
8.0 7.5 7.0 6.5 6.0 5.5 5.0 4.5 4.0 3.5 3.0 2.5 2.0 1.5 1.0 0.5 0.0
f1 (ppm)

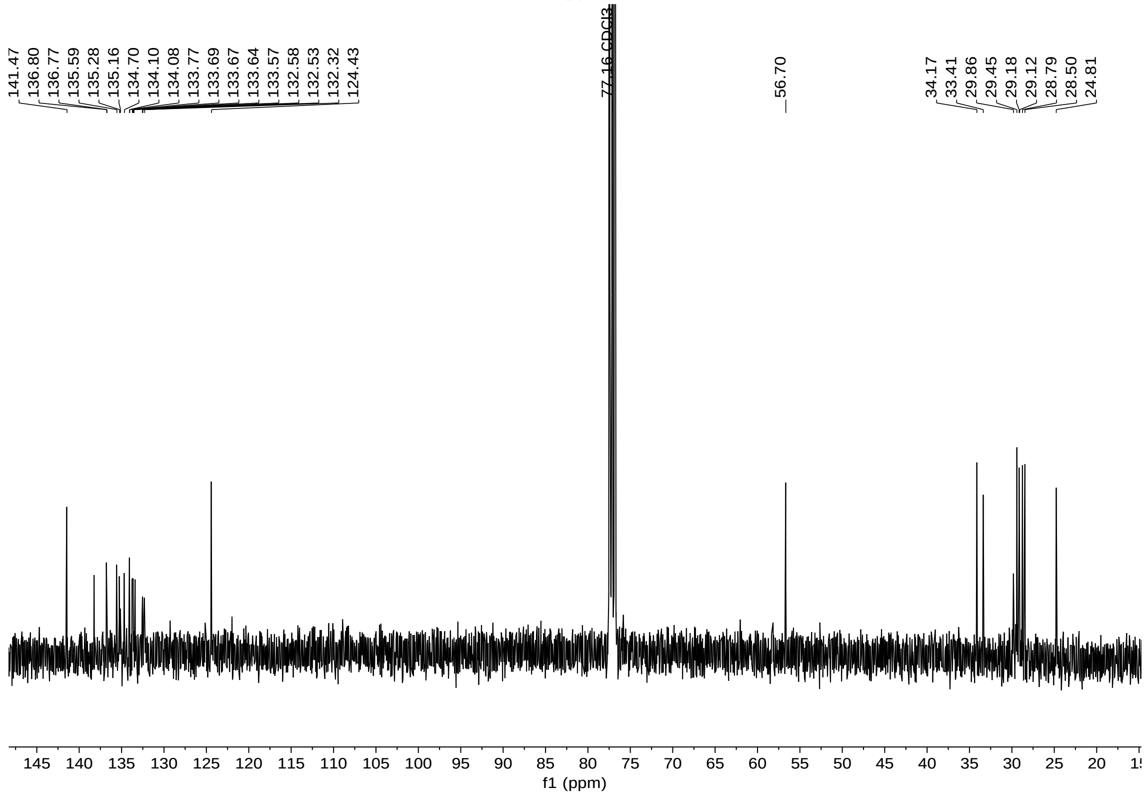
141.47
136.80
136.77
135.59
135.28
135.16
134.70
134.10
134.08
133.77
133.69
133.67
133.64
133.57
132.58
132.53
132.32
124.43
77.16 CDCl3
56.70
34.17
33.41
29.86
29.45
29.18
29.12
28.79
28.50
24.81
145 140 135 130 125 120 115 110 105 100 95 90 85 80 75 70 65 60 55 50 45 40 35 30 25 20
f1 (ppm)

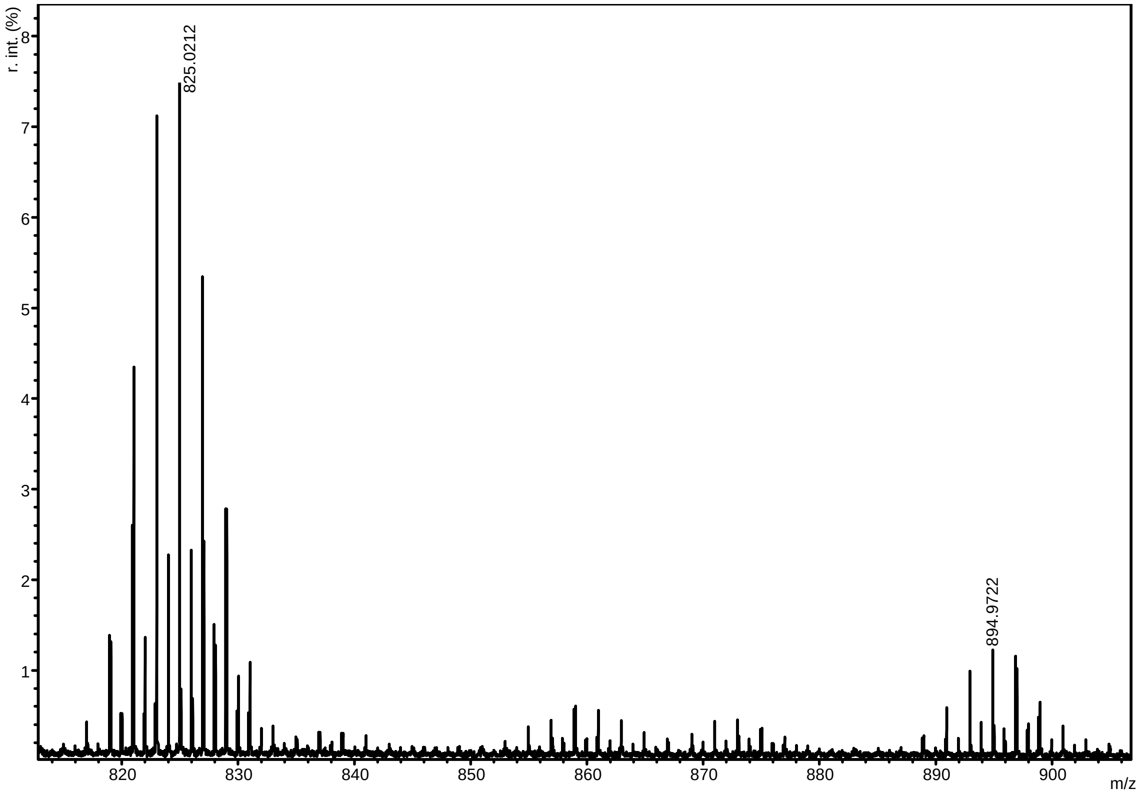

(E)-12-(4-(bis(perchlorophenyl)methyl)-2,3,5,6-tetrachlorophenyl) dodec-11-ene-1-thiol (NR12)

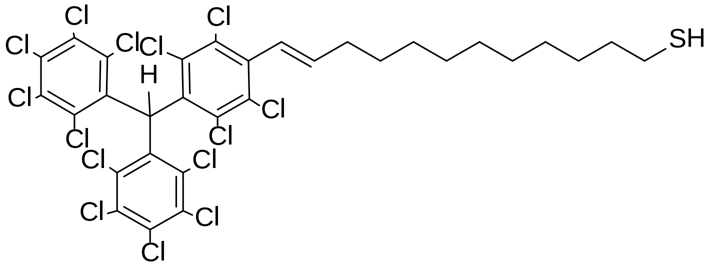

The compound **NR12** was obtained as withe powder in quantitative yield, using the general methodology described for the generation of free thiols. **^{1}H-NMR** (400 MHz, $CDCl_3$) *δ*/ppm: 7.00 (s, 1H), 6.31 (d, *J* = 16.2 Hz, 1H), 6.15 (dt, *J* = 16.1, 6.9 Hz, 1H), 2.52 (q, *J* = 7.4 Hz, 2H), 2.30 (q, *J* = 6.7 Hz, 2H), 1.67 – 1.45 (m, 5H), 1.45 – 1.12 (m, 16H); **^{13}C-NMR** (101 MHz, $CDCl_3$) δ/ppm: 141.54, 138.27, 136.80, 136.77, 135.58, 135.28, 135.17, 134.70, 134.10, 134.08, 133.77, 133.69, 133.67, 133.64, 133.58, 133.42, 132.58, 132.54, 132.33, 124.39, 77.16, 56.70, 34.20, 33.44, 29.86, 29.82, 29.68, 29.65, 29.56, 29.52, 29.22, 29.20, 28.83, 28.53, 24.81, 22.85; **FT-IR** (ν/cm^{-1}): 2924.5, 2852.6, 1717.8, 1653.2, 1532.6, 1461.2, 1365.3, 1336.1, 1296.1, 1263.7, 1238.8, 1137.4, 966.7, 854.8, 807.6, 737.8, 717.6, 700.1, 674.9, 648.0, 606.9; **EM (m/z)** (MALDI-TOF): calculated for $C_{31}H_{24}Cl_{14}S$: 923.71; found: 922.93 (M-1H) and 852.99 (M-2Cl).

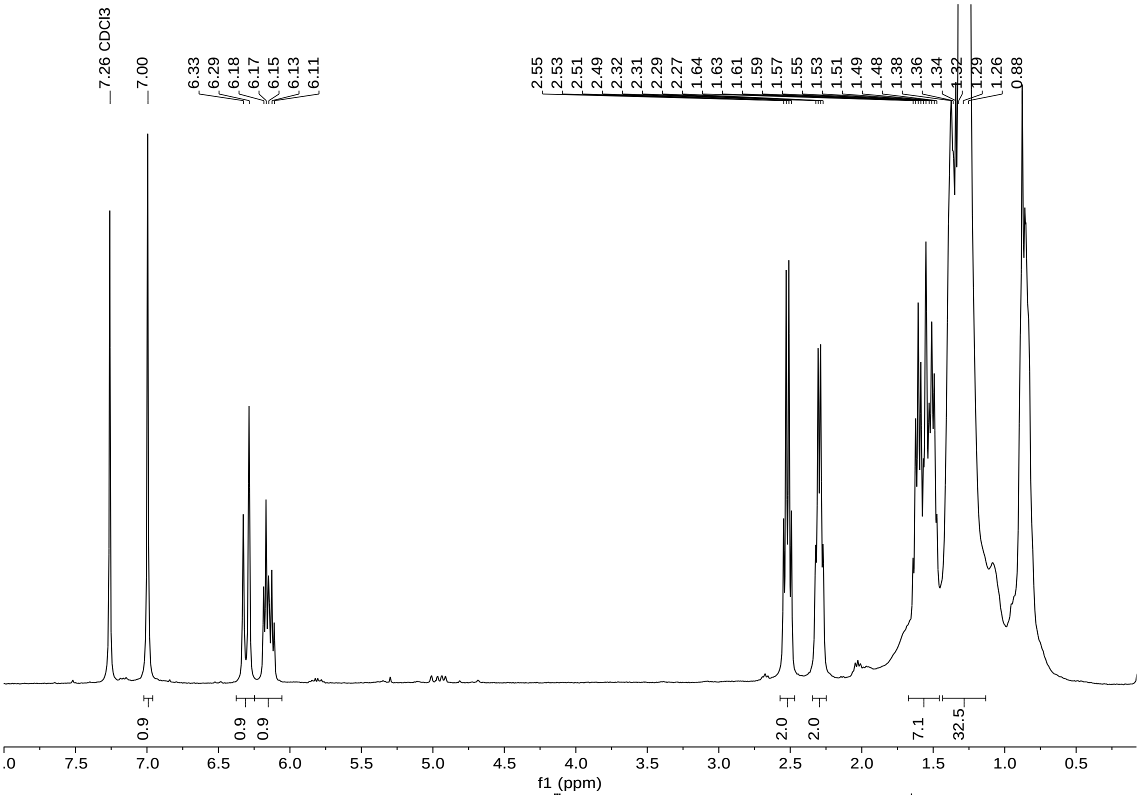
7.26 CDCl3
7.00
6.33 6.29 6.18 6.17 6.15 6.13 6.11
2.55 2.53 2.51 2.49 2.32 2.31 2.29 2.27 1.64 1.63 1.61 1.59 1.57 1.55 1.53 1.51 1.49 1.48 1.38 1.36 1.34 1.32 1.29 1.26 0.88
0.9 0.9 0.9 2.0 2.0 7.1 32.5
f1 (ppm)

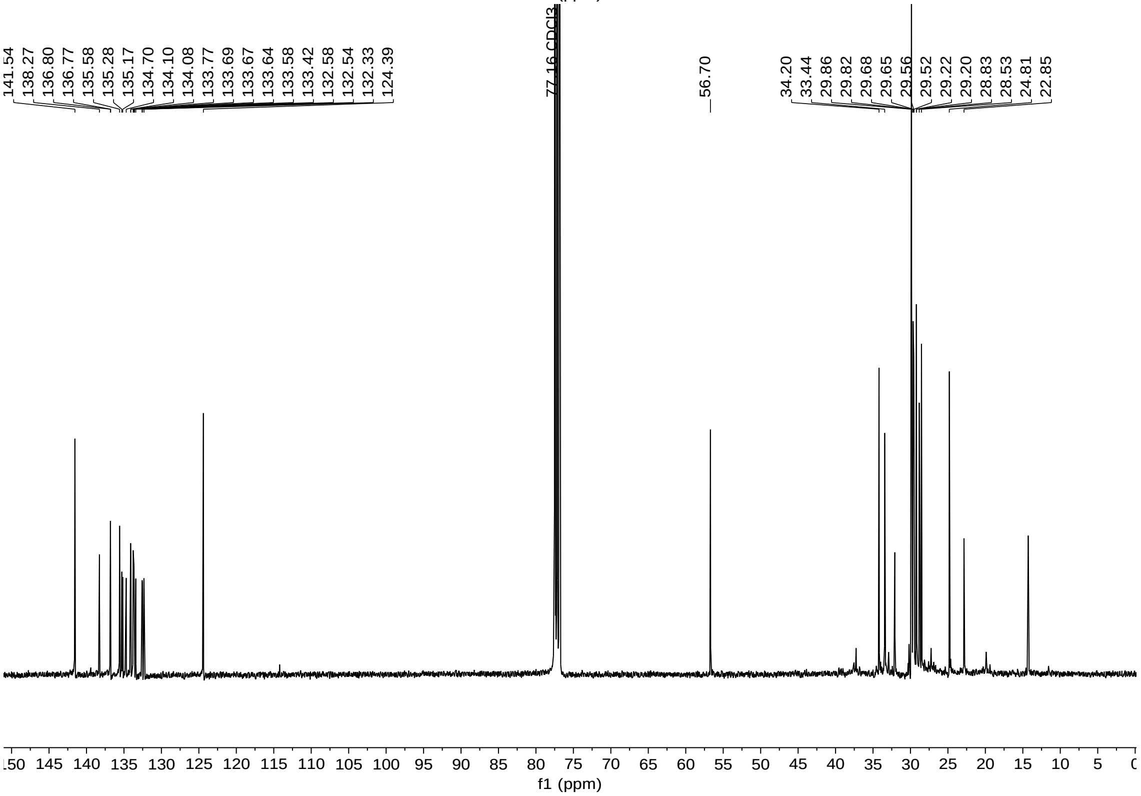
141.54 138.27 136.80 136.77 135.58 135.28 135.17 134.70 134.10 134.08 133.77 133.69 133.67 133.64 133.58 133.42 132.58 132.54 132.33 124.39
77.16 CDCl3
56.70
34.20 33.44 29.86 29.82 29.68 29.65 29.56 29.52 29.22 29.20 28.83 28.53 24.81 22.85
f1 (ppm)

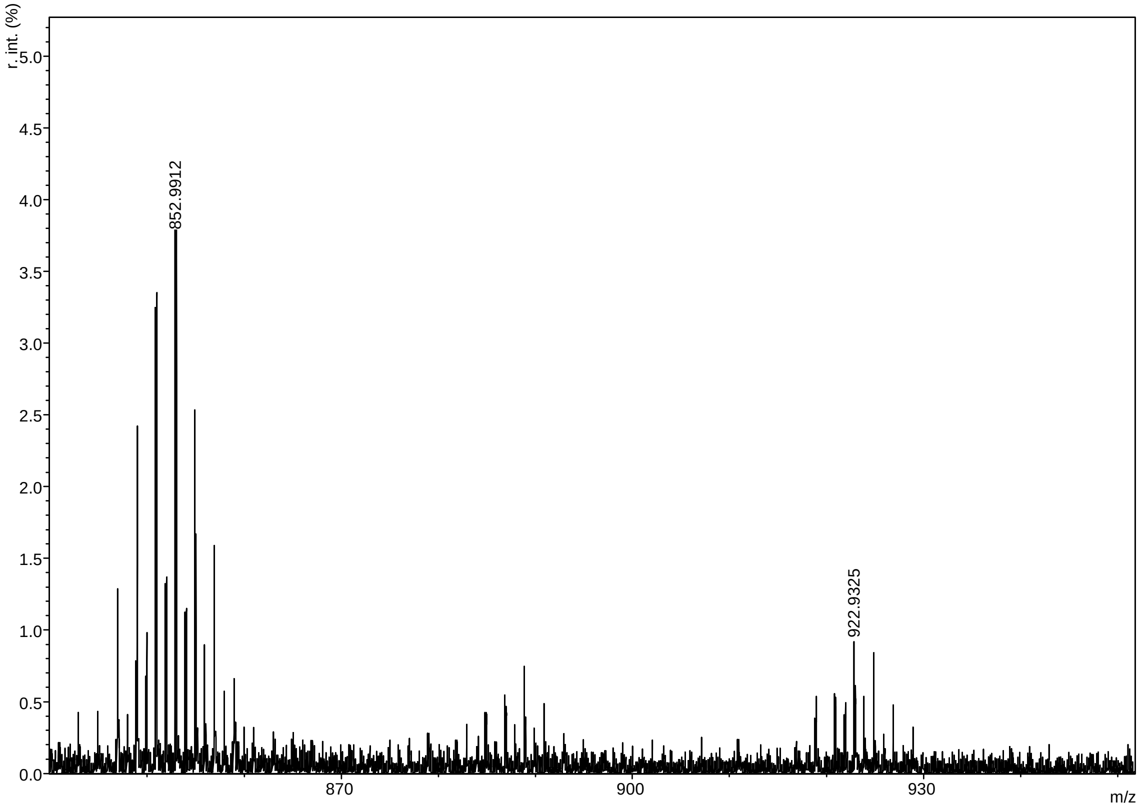

(E)-8-(4-(bis(perchlorophenyl)methyl)-2,3,5,6-tetrachlorophenyl)oct-7-ene-1-thiol radical (R8)

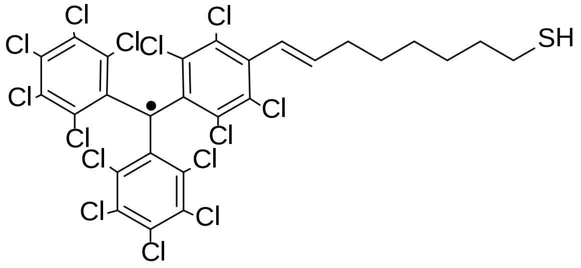

The compound **R8** was obtained as a red powder in quantitative yield, using the general methodology described for the generation of the free thiol groups. **HPLC**, retention time 13.8min: (97% of trans isomer); **UV/Vis** (CH_2Cl_2): λ*(nm)* (logε) = 387 (4.43), 515 (3.10), 566 (3.14); **CV**: $E^{1/2}$ = -0.18 V (PTM reduction); **FT-IR** (ν/cm^{-1}): 3025.5, 2926.1, 2853.6, 1647.8, 1597.7, 1511.2, 1494.2, 1450.8, 1332.3, 12594.4, 1157.4, 1120.1, 1078.6, 1049.1, 1031.4, 966.1, 860.2, 815.8, 752.9, 732.7, 698.1, 651.7, 605.1; **EPR** (CH_2Cl_2, r.t.): g = 2.002552; ΔH_{PP} = 1.4 G, $a_{13C\alpha}$ = 29.8 G; a_{13Co} = 13.1 G; a_{13Cm} = 10.1 G; a_{1H} = 1.8 G; EM (m/z) (ESI): calculated for $C_{27}H_{15}Cl_{14}S^{\cdot}$: 866.6; found: 866.7 (M*).

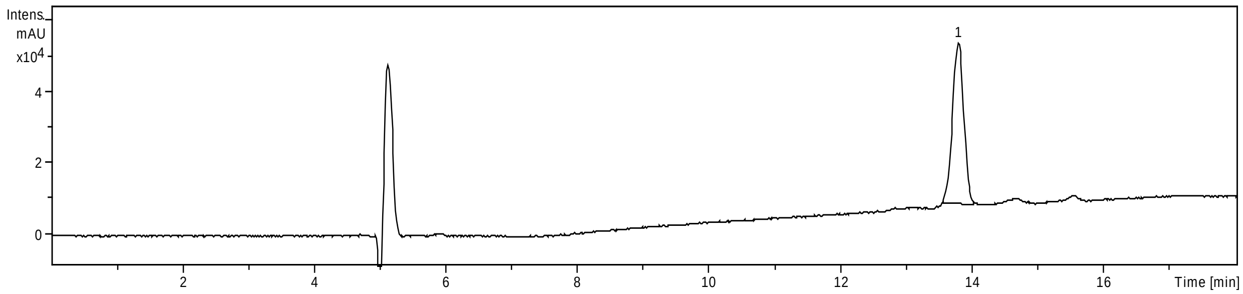

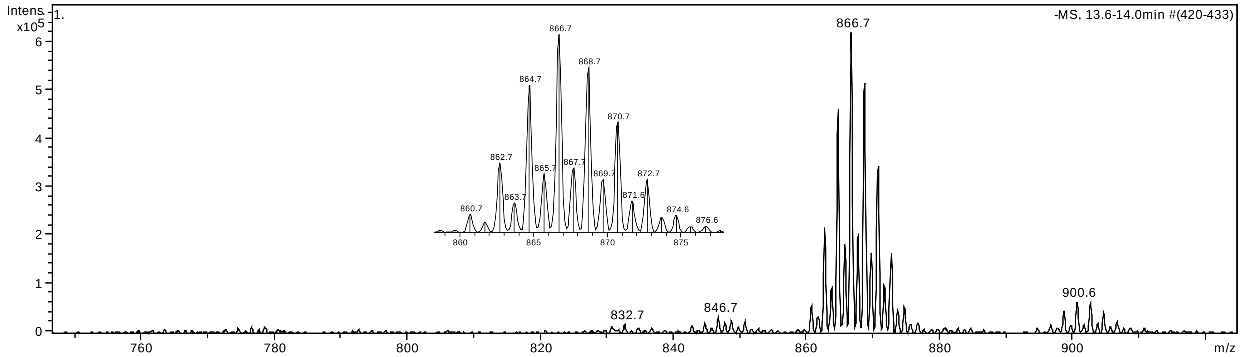

(E)-10-(4-(bis(perchlorophenyl)methyl)-2,3,5,6-tetrachlorophenyl)dec-9-ene-1-thiol radical (R10)

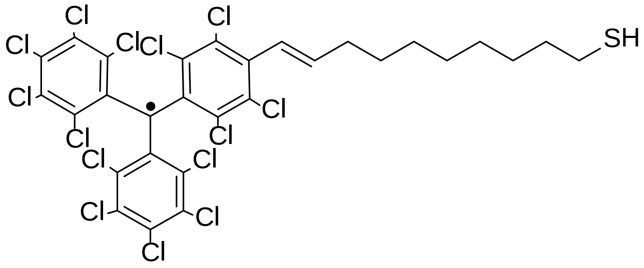

The compound **R10** was obtained as a red powder in quantitative yield, using the general methodology described for the generation of the free thiol groups. **HPLC** retention time: 15.2min (98% of trans isomer); **UV/Vis** (CH_2Cl_2): $\lambda(nm)$ ($\log\varepsilon$) = 388 (4.46), 515 (3.08), 566 (3.12); **CV**: $E^{1/2}$ = -0.18 V (PTM reduction); **FT-IR** (ν/cm^{-1}): 3026.8, 2924.7, 2852.7, 1647.37, 1598.3, 1511.0, 1494.9, 1455.2, 1332.3, 1258.9, 1157.4, 1118.9, 1048.3, 965.7, 860.1, 815.4, 750.7, 732.9, 698.9; **EPR** (CH_2Cl_2, r.t.): g = 2.002431; ΔH_{PP} = 1.4 G, $a_{13C\alpha} = 29.5$ G; a_{13Co} = 13.0 G; a_{13Cm} = 10.0 G; a_{1H} = 1.8 G; **EM (m/z)** (ESI): calculated for $C_{29}H_{19}Cl_{14}S^{\cdot}$: 894.7; found: 894.7 (M*).

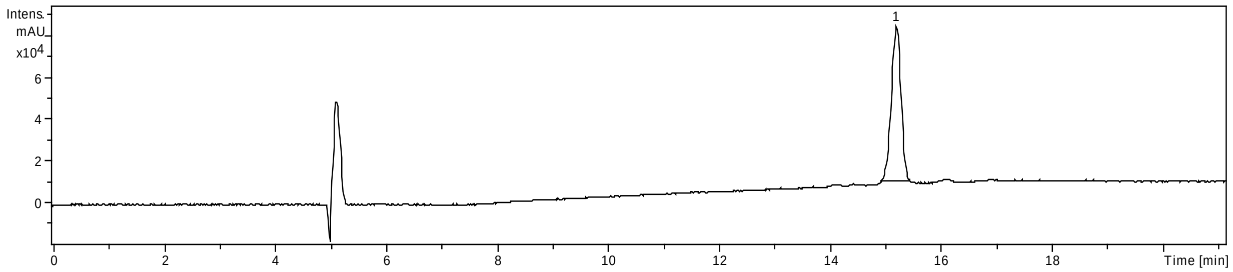

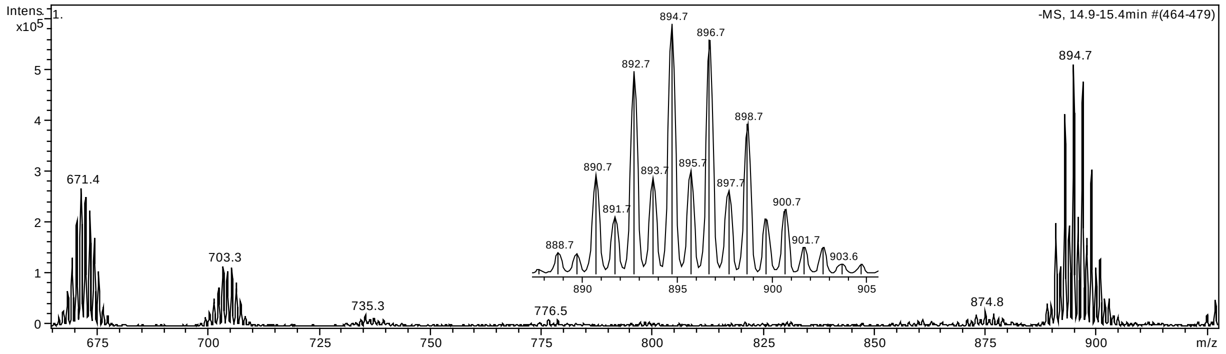

(E)-12-(4-(bis(perchlorophenyl)methyl)-2,3,5,6-tetrachlorophenyl) dodec-11-ene-1-thiol radical (R12)

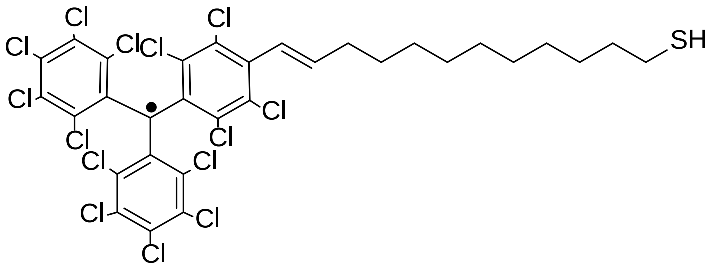

The compound **R12** was obtained as a red powder in quantitative yield, using the general methodology described for the generation of the free thiol groups. **HPLC** retention time: 21.1min (92% of trans isomer), 22.8 (8% of αH derivative); **UV/Vis** (CH_2Cl_2): $\lambda(nm)$ (logε) = 387 (4.50), 515 (3.13), 566 (3.16); **CV**: $E^{1/2}$ = -0.18 V (PTM reduction); **FT-IR** (ν/cm^{-1}):2924.4, 2852.4, 1648.6, 1510.6, 1460.7, 1332.3, 1259.1, 1157.1, 1119.7, 1048.3, 966.4, 860.2, 815.7, 752.1, 733.9, 707.0, 651.4, 606.1; **EPR** (CH_2Cl_2, r.t.): g = 2.002803; ΔH_{PP} = 1.2 G, $a_{13C\alpha}$ = 29.8 G; a_{13Co} = 13.2 G; a_{13Cm} = 10.3 G; a_{1H} = 1.8 G; **EM (m/z)** (ESI): calculated for $C_{31}H_{23}Cl_{14}S^{\cdot}$: 922.7; found: 922.7 (M*).

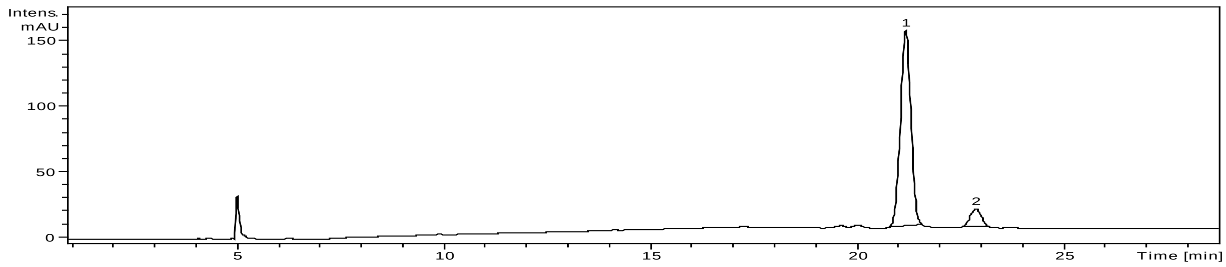

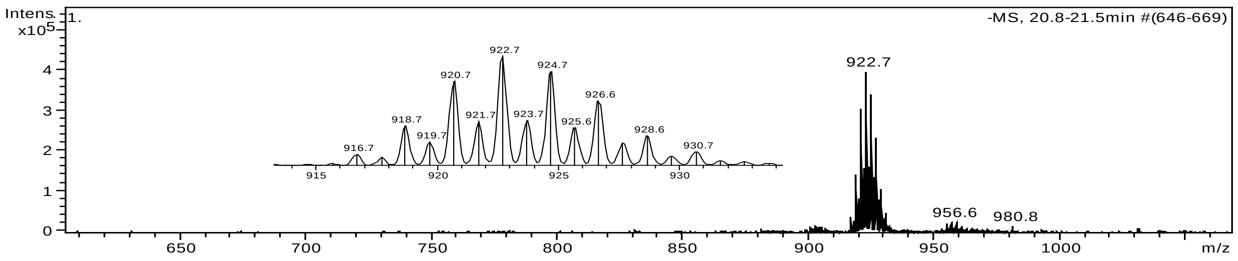

Intens.
x10^5
-MS, 20.8-21.5min #(646-669)
922.7
956.6
980.8
916.7
918.7
919.7
920.7
921.7
922.7
923.7
924.7
925.6
926.6
928.6
930.7
915
920
925
930
650
700
750
800
850
900
950
1000
m/z
